# Novel Glu-based pyrazolo[3,4-d]pyrimidine analogues: design, synthesis and biological evaluation as DHFR and TS dual inhibitors

**DOI:** 10.1080/14756366.2023.2203879

**Published:** 2023-04-20

**Authors:** Mater Mahnashi, Mohammed Merae Alshahrani, Amer Al Ali, Abdulaziz Asiri, Mahrous A. Abou-Salim

**Affiliations:** a Department of Pharmaceutical Chemistry, College of Pharmacy, Najran University, Najran, Saudi Arabia; b Department of Clinical Laboratory Sciences, Faculty of Applied Medical Sciences, Najran University, Najran, Saudi Arabia; c Department of Clinical Laboratory Sciences, Faculty of Applied Medical Sciences, University of Bisha, Bisha, Saudi Arabia; d Pharmaceutical Organic Chemistry, Faculty of Pharmacy, Al-Azhar University, Assiut, Egypt

**Keywords:** DHFR, TS, pyrazolo[34-d]pyrimidine, NCI, Glu

## Abstract

A novel series of multifunctional pyrazolo[3,4-d]pyrimidine-based glutamate analogs (**6a–l** and **7a,b**) have been designed and synthesized as antifolate anticancer agents. Among the tested compounds, **6i** exhibited the most potent anti-proliferative activity towards NSCLC, CNS, Ovarian, Prostate, Colon, Melanoma, Breast, and Renal cancers with good to weak cytostatic activity and non-lethal actions. **6i** demonstrated higher selectivity for cancer than normal cells. **6i** could significantly increase the accumulation of S-phase cells during the cell cycle distribution of cancer cells with high potency in the induction of apoptosis. The results unveiled that **6i** probably acts through dual inhibition of DHFR and TS enzymes (IC_50_ = 2.41 and 8.88 µM, correspondingly). Docking studies of **6i** displayed that N1-*p*-bromophenyl and C3-Methyl groups participate in substantial hydrophobic interactions. The drug-likeness features inferred that **6i** met the acceptance criteria of Pfizer. Taking together, **6i** could be a promising prototype for further optimization as an effective anticancer drug.

## Introduction

**Figure 1.**
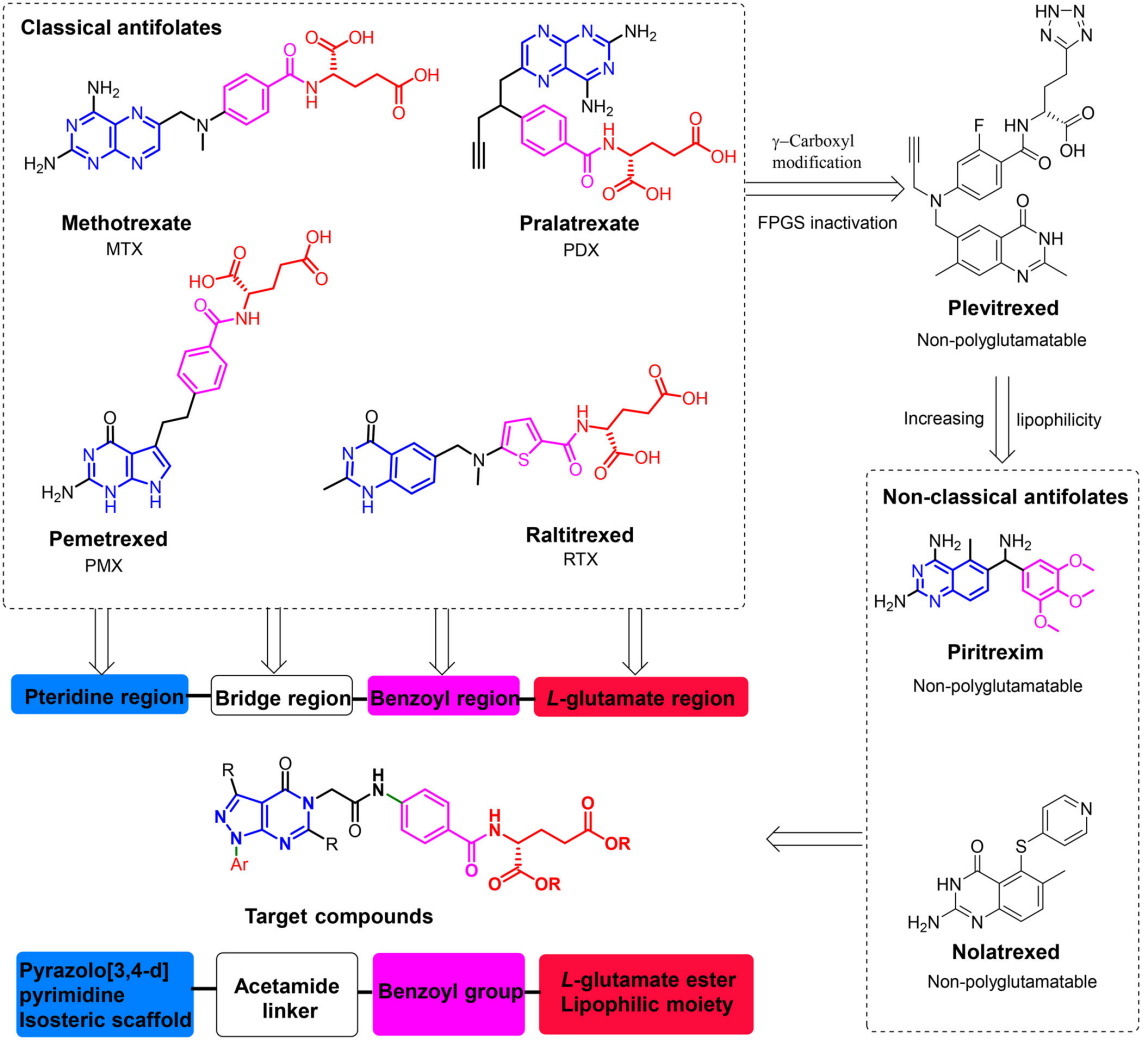
Structures of some antifolates and design strategy of novel Glu-based pyrazolo[3,4-d]pyrimidine analogues.

During the process of DNA synthesis, a significant number of endogenic forces can challenge, and cells harbour a series of pathways to disseminate genome integrity^1^. Dihydrofolate reductase (DHFR) and thymidylate synthetase (TS) are key enzymes in nucleic acid synthesis, and they have long been evidenced as a crucial target of cancer chemotherapy^2^^,^^3^. DHFR reduces dihydrofolate (DHF) to tetrahydrofolate (THF) using NADPH, while TS catalyses the *de novo* synthesis of deoxythymidine monophosphate (dTMP) from deoxyuridine monophosphate (dUMP) using 5,10-Methylene THF as a cofactor, and thus initiates DNA synthesis and cell proliferation^2^^,^^3^. Therefore, DHFR and TS inhibitors cause THF and dTMP deficiency, resulting in RNA and DNA synthesis interference and apoptosis^4^. However, the dose-limiting toxicities, low solubility, short plasma half-life, low specificity, rapid diffusion throughout the body, weak brain permeability unless at high doses (1–8 g/m^2^), low absorption and drug resistance development are the main drawbacks of the first-in-class DHFR and TS inhibitors^3–5^. A major mechanism of drug resistance by target cells to clinically useful classical antifolates (Figure 1) is based on their need for polyglutamylation via the enzyme folylpoly-gamma-glutamate synthetase (FPGS), which catalyses the addition of several equivalents of glutamic acid to the γ-carboxyl group in the side chain and hence prevents drug efflux from the cell^6^. Therefore, developing a novel antifolate class of low toxicity and tumour resistance remains one of the major challenges that incessantly attracts intense interest^2^^,^^3^^,^^5^^,^^7–10^.

The basic pharmacophoric features of classical antifolates such as methotrexate (MTX)^3^, pralatrexate (PDX)^3^, pemetrexed (PMX)^3^, and raltitrexed (RTX)^3^ could be divided into four regions as shown in Figure 1: (1) the fused heterocyclic system (substituted pteridine, pyrrolo[2,3-d]pyrimidine and quinazoline scaffolds) which occupies the pteridine binding domain; (2) the heteroalkyl/alkyl chain (*N*-methyl aminomethyl, ethyl and *sec*-pentyne moieties) which occupies the bridge region and allows a tortuous “L” structure formation to fit tightly into the pocket of DHFR; (3) the benzoyl group which occupies the benzoyl region; and (4) the glutamate tail which occupies the *l*-glutamic acid binding site^3^. The SAR studies of classical antifolates usually vary in these regions.

The next generation of antifolates, as plevitrexed, revealed similar pharmacophoric properties of classical ones in which the free γ-carboxylic group was replaced with bioisosteric tetrazole moiety which, in turn, either improved the drug absorption or did not need to be polyglutamylated by FPGS and hence allows more decrease in drug resistance. In this context, the nonclassical antifolates such as nolatrexed and piritrexim (PTX) were designed as they are more lipophilic and non-polyglutamatable agents^11^^,^^12^. Modifications in the antifolates structures have helped delineate the structural influence on the interaction with DHFR, TS, and FPGS utilisation. Therefore, intense interest has been in developing a new class of antifolate agents with these crucial structural pharmacophoric properties.

On the basis of isosterism and combination principles, as shown in Figure 1, the 6-5 fused system, pyrazolo[3,4-d]pyrimidine, was selected as pteridine core isostere since it has recently been reported to display potent anti-tumour activities against different cancer cells with multitarget properties^13–16^. The pyrazolo[3,4-d]pyrimidine core varied at N1, C3, and C6 and bridged at N5 with an acetamide linker which ended up with pharmacophoric benzoyl glutamate tail^3^. The linker acetamide was used to improve binding affinity through the formation of a tortuous “L” structure to fit tightly inside the active site. In the glutamate region, both α and γ free carboxyl groups were blocked by making the corresponding ester in the form of a prodrug as a chemical approach catalysed by metabolic enzymes to minimise the undesirable physicochemical properties and enhance lipophilicity and obtain non-polyglutamatable agents^4^^,^^17^. Moreover, the fused pyrimidine scaffold was replaced by a pyrimidine core to study the effect of a 6-5 fused system on biological activity. Therefore, we aim to develop novel hDHFR and TS dual inhibitors as a new generation of novel effective multitarget antifolate prodrugs with fewer side effects. Herein, a new lipophilic Glu-based pyrazolo[3,4-d]pyrimidine hybrid prodrug candidates were designed, prepared, and characterised by different spectroscopic techniques. The synthesised analogues were examined for their growth inhibitory (GI) effect on 59 cancer cell lines. The most active analogue was further subjected for its *in vitro* anti-proliferative activity against the most sensitive tumour and normal cell lines to investigate its selectivity towards cancer cells. In addition, cell cycle analysis and apoptosis were also examined. Finally, the *in-silico* molecular modelling and ADMET analysis were reported.

## Results and discussions

### Chemistry

**Scheme 1.**
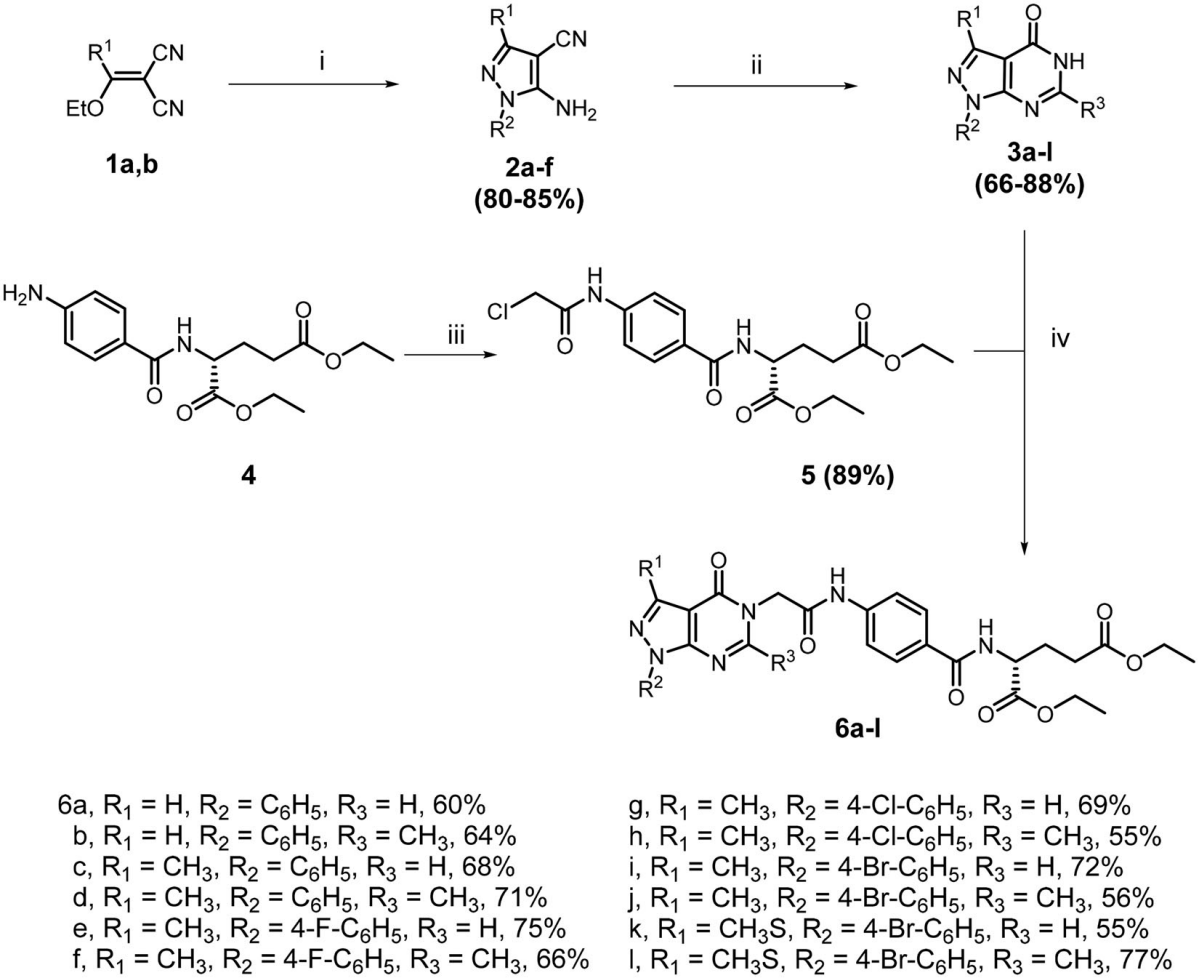
Synthesis of target compounds **6a–l**. (i) R_2_-NHNH_2_, EtOH, reflux, 6 h; (ii) R_3_COOH, POCl_3_, reflux, 2h; (iii) ClCH_2_COCl, THF, reflux, 1 h; (iv) **5**, DMF, K_2_CO_3_, NaI, 80 °C, 1–2 h.

The Glu-based pyrazolo[3,4-d]pyrimidine analogues **6a–l** were prepared as cited in Scheme 1. The reaction cascade began with the cyclisation of alkylidene malononitrile **1a,b** to 5-amino-4-cyano-pyrazole derivatives **2a–f** using arylhydrazine derivatives^18^^,^^19^. The intermolecular cyclisation of 5-amino-4-cyano-pyrazole derivatives **2a–f** using different aliphatic acids with a catalytic amount of phosphorous oxychloride afforded the precursors pyrazolo[3,4-d]pyrimidine-4-ones **3a–l**^20^. On the other hand, the glutamate chloroacetamide intermediate **5** was obtained from the chloroacetylation of glutamate anilinic derivative **4** with chloroacetyl chloride. Finally, the desired analogues **6a–n** were synthesised through N5-alkylation of the precursor pyrazolo[3,4-d]pyrimidine-4-ones **3a–l** with diethyl glutamate chloroacetamide intermediate **5**.

**Scheme 2.**
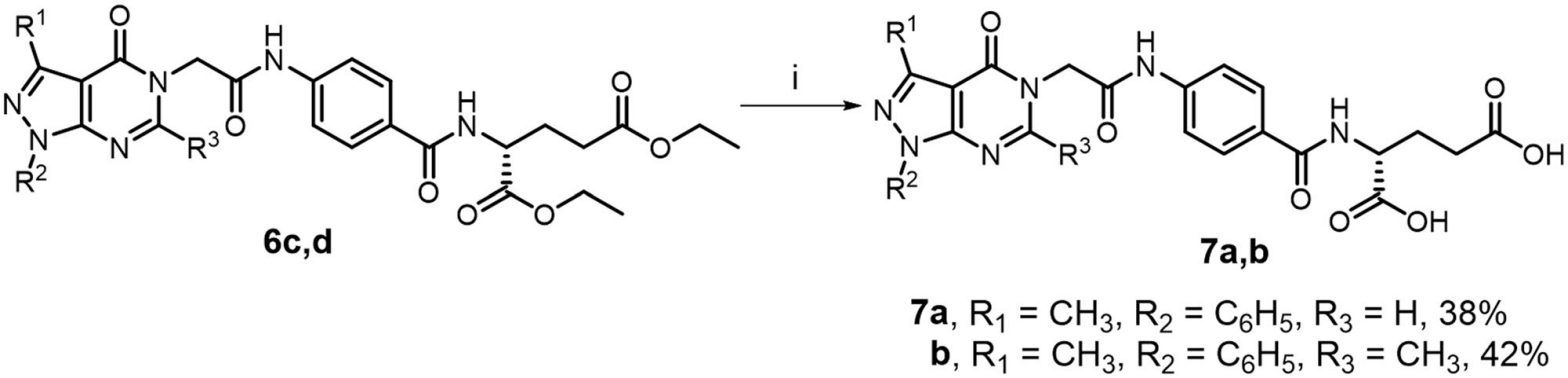
Synthesis of acid derivatives **7a,b**. (i) aq. NaOH, 25 °C, 2h.

**Scheme 3.**
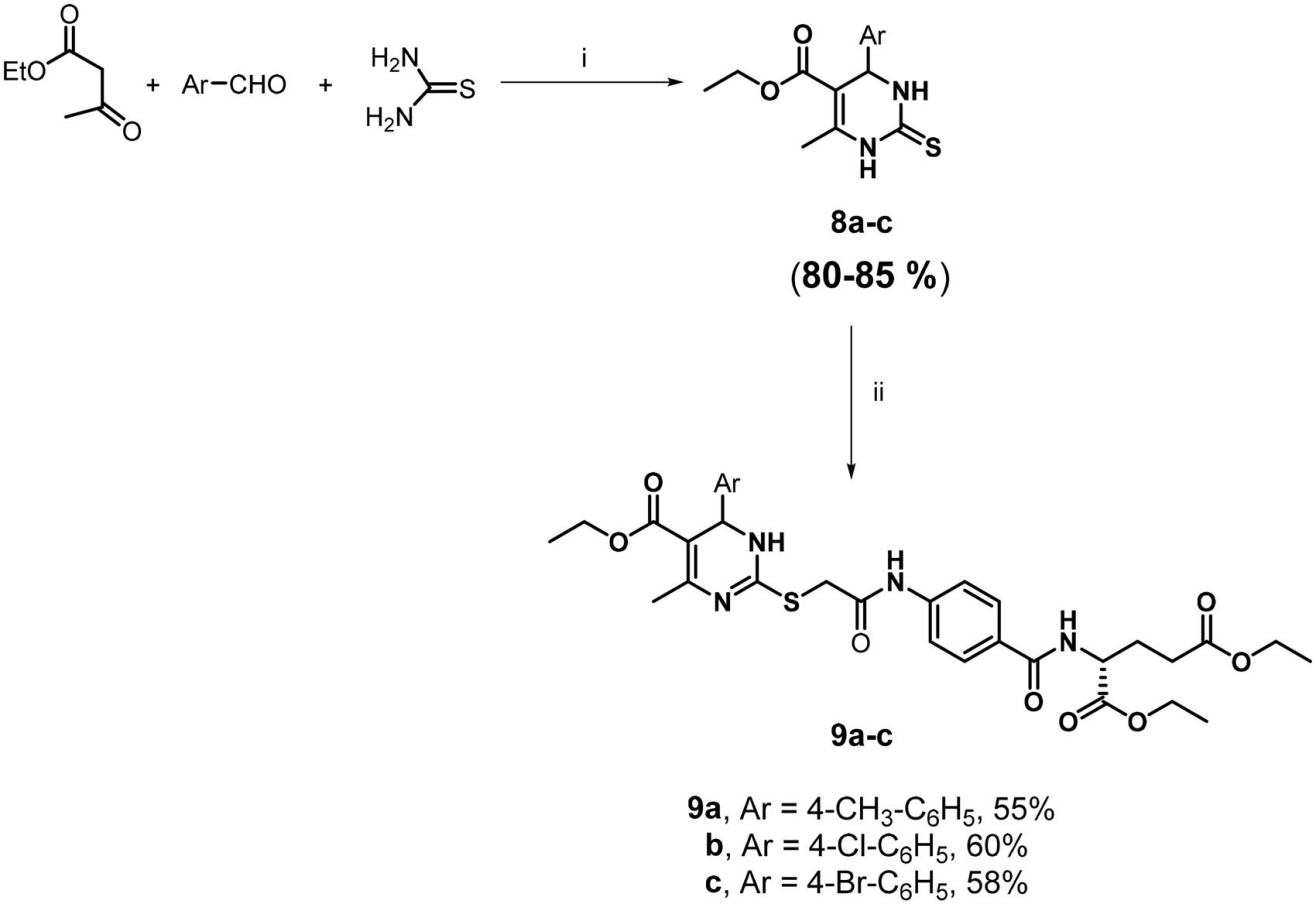
Synthesis of substituted 1,4-dihydropyrimidine derivatives **9a–c. (**i) HCl, EtOH, reflux, 6 h; (ii) **5**, DMF, K_2_CO_3_, NaI, RT, 8 h.

Moreover, as shown in Scheme 2, carboxylic acid derivatives **7a** and **7b** were prepared through alkaline hydrolysis of the corresponding diethyl ester analogues **6c** and **6d**, respectively. On the other hand, the Biginelli reaction was utilised to synthesise the 1,4-dihydropyrimidine derivatives **9a–c** through *S*-alkylation of 2-thioxo-1,4-dihydropyrimidine intermediates **8a–c** with chloroacetamide glutamate derivative **5** as depicted in Scheme 3. Both spectral and elemental analysis data are depicted in the Supporting Information and have been fully consistent with the postulated structures of the prepared compounds.

### Biological evaluation

#### Preliminary NCI-59 cancer cells single-dose screen

The growth percentage (G%) and cell viability of the herein target compounds **6a–l, 7a,b**, and **9a–c** have been screened towards a full panel of 59 cancer cells at NCI’s Developmental Therapeutics Program (NCI-DTP, MD, USA)^21^. The submitted analogues were initially examined for preliminary *in vitro* anticancer activity using sulforhodamine B (SRB) assay at 10 µM as a single dose to evaluate cell growth and viability according to the procedure of Drug Evaluation Branch^22^^,^^23^.

Different types of tumours – colon, NSCLC, leukaemia, CNS, renal, breast, ovarian, melanoma, and prostate cancers – were investigated as a full panel of 59 cell lines, and the attained results are cited in Figures S1–S17 (see Supplementary Material) where the value recorded is the growth relative to that of both the negative control and zero-time number of cells in which both growth inhibition (GI%; 0–100%) and lethality (L%; 0–100%) percentages can be identified. The growth inhibition means percentages (MGI%) for the examined target analogues towards the different treated 59 cancer cells were presented in Figure 2.

**Figure 2.**
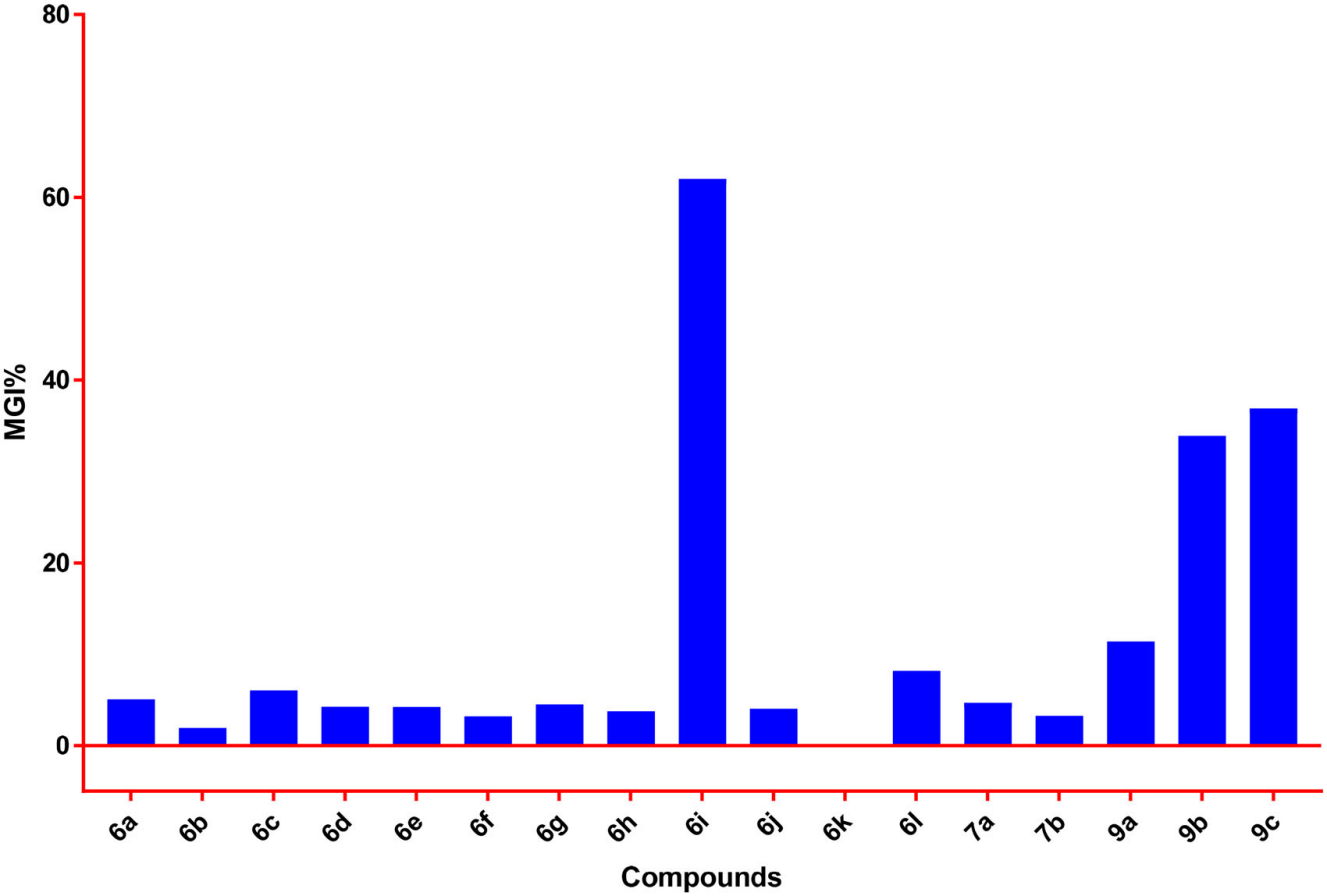
MGI% for compounds **6a–l** and **9a–c** towards a full panel of 59 cell lines.

The attained results disclosed that compound **6k** failed to demonstrate MGI%, while compounds **6a–h, 6j, 6 l**, **7a,b**, and **9a** showed weak anticancer activity with MGI% ranging from 1.93% to 11.40%. Compounds **9b** and **9c** demonstrated moderate anticancer activity, with MGI% equal to 33.89% and 36.88%, respectively, while compound **6i** exerted the significant mean growth inhibition percentage of 62.03% among the herein examined series. Superiorly, compound **6i** emerged as the most efficient anticancer analogue eliciting high potency with broad-spectrum anticancer activity against all the herein subsets of cell panels, except against the A498 (Renal cancer) cell line (Figure 2).

**Figure 3.**
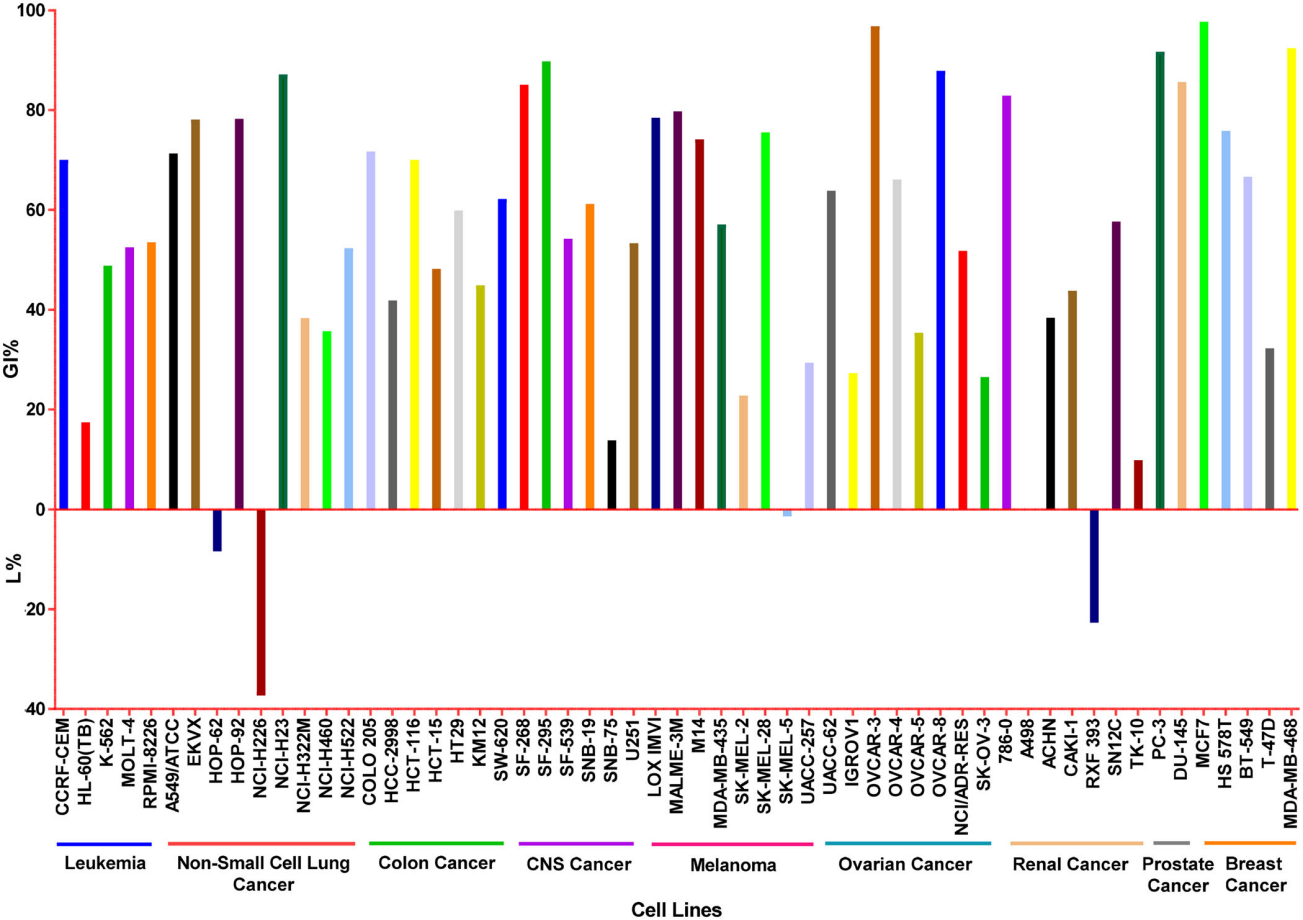
GI% of the most active derivative **6i** against NCI-59 cancer cells.

The inhibitory growth percentages (GI%) of compound **6i** against the herein examined 59 cell panel are presented in Figure 3. Surprisingly, exploring the results hinted that compound **6i** exhibited excellent anti-proliferative activities towards Breast (MDA-MB-468 and MCF7), CNS (SF-295 and SF-268), NSCLC (NCI-H23), Ovarian (OVCAR-3 and OVCAR-8), Prostate (DU-145 and PC-3) and Renal (786-0) cancer cell lines with GI% of 92.44, 97.72, 89.78, 85.11, 87.19, 96.83, 87.86, 85.62, 91.71, and 82.93%, respectively. In addition, compound **6i** displayed good inhibitory activity against Breast cancer (HS 578 T and BT-549), CNS cancer (SNB-19), Colon cancer (HCT-116, COLO 205, and SW-620), Leukaemia (CCRF-CEM), Melanoma (CCRF-CEM, LOX IMVI, SK-MEL-28, M14, and UACC-62), NSCLC (HOP-92, EKVX, and A549/ATCC) and Ovarian cancer (OVCAR-4) cell lines with GI% equals to 75.80, 66.64, 61.23, 70.04, 71.69, 62.17, 70.05, 79.78, 78.49, 75.54, 74.15, 63.82, 78.29, 78.12, 71.36, and 66.09, correspondingly. Furthermore, compound **6i** demonstrated weak inhibitory activity against CNS cancer (SF-539 and U251), Colon cancer (HT29), Leukaemia (RPMI-8226 and MOLT-4), Melanoma (MDA-MB-435), NSCLC (NCI-H522), Ovarian cancer (NCI/ADR-RES) and Renal cancer (SN12C) cell lines with GI% of 54.23, 53.30, 59.90, 53.52, 52.51, 57.10, 52.34, 51.78, and 57.69, respectively. Moreover, the growth inhibitory percentages of **6i** towards the other cell lines ranged from 9.83 to 48.82%. It is worth underlining that the lethality (L) of compound **6i** was detected against some cell lines such as NSCLC (NCI-H226 and HOP-62), Renal (RXF 393), and Melanoma (SK-MEL-5) cancer cell lines with L% of 37.32, 8.41, 22.73, and 1.38%, respectively. Therefore, compound **6i** exerted a remarkable anticancer profile in the One-Dose assay towards the herein tumour screening panel.

#### NCI 59 cancer cell five-dose screen

Remarkably, compound **6i** (NSC: 838397) exhibited significant growth inhibition properties in the One-Dose (10 µM) screening assay; hence it was chosen for further evaluation at five-dose levels (0.01–100 µM) towards the herein examined tumour cell panels^21^ (Figures S53–S56 in Supporting Information). This assay evaluates GI_50_, TGI, and LC_50_ parameters, as cited in Table 1. Their values correspondingly represent the GI level, cytostatic impact, and cytotoxicity parameter. Furthermore, based on the GI_50_ values, the mean graph midpoints (MG-MID) for both full NCI 59 cell panel and subpanel cancer cell lines were evaluated, affording a median potency parameter for the examined compound **6i**, as depicted in Table 2.

**Table 1.** NCI-59 cancer cell five-dose screening results of compound **6i** (NSC: 838397).

Subpanel/cancer cell lines		Compound 6i	
	GI_50_ (µM)	TGI (µM)	LC_50_ (µM)
Leukaemia			
HL-60(TB)	>100	>100	>100
CCRF-CEM	14.5	>100	>100
MOLT-4	>100	>100	>100
K-562	>100	>100	>100
SR	71.0	>100	>100
RPMI-8226	22.7	>100	>100
Non-small cell lung cancer			
EKVX	10.4	27.3	71.7
A549/ATCC	9.81	57.3	>100
HOP-62	5.11	19.8	57.5
HOP-92	6.01	23.7	66.2
NCI-H226	4.54	27.8	>100
NCI-H23	7.64	26.7	82.1
NCI-H322M	21.3	66.6	>100
NCI-H460	13.7	34.4	86.9
NCI-H522	17.1	38.2	85.0
Colon cancer			
COLO 205	14.0	36.7	96.0
HCC-2998	35.1	>100	>100
HCT-116	9.85	>100	>100
SW-620	8.98	44.4	>100
HCT-15	26.0	>100	>100
KM12	16.1	>100	>100
HT29	18.4	71.7	>100
CNS cancer			
SF-268	7.01	32.8	>100
SF-295	14.2	31.9	71.6
SF-539	14.8	33.0	73.6
SNB-19	12.1	27.3	61.3
SNB-75	14.9	40.9	>100
U251	13.2	>100	>100
Melanoma			
M14	>100	>100	>100
LOX IMVI	5.3	20.6	58.4
MALME-3M	15.0	29.9	59.4
SK-MEL-2	27.6	89.1	>100
MDA-MB-435	12.2	34.3	96.1
SK-MEL-5	19.4	43.1	95.8
SK-MEL-28	10.1	22.5	50.2
UACC-62	13.3	32.1	77.8
UACC-257	31.5	>100	>100
Ovarian cancer			
OVCAR-3	11.7	61.3	>100
IGROV1	23.9	74.2	>100
OVCAR-5	20.2	56.3	>100
OVCAR-4	13.4	32.1	76.7
SK-OV-3	21.8	67.3	>100
OVCAR-8	8.17	>100	>100
Renal cancer			
A498	24.9	48.1	93.0
786-0	8.66	>100	>100
CAKI-1	10.4	22.1	47.0
ACHN	15.1	34.3	78.3
SN12C	29.2	>100	>100
RXF 393	15.9	50.9	>100
UO-31	18.1	55.9	>100
TK-10	31.3	86.2	>100
Prostate cancer			
DU-145	7.47	28.8	>100
PC-3	5.79	19.4	47.7
Breast cancer			
MDA-MB-231/ATCC	11.5	26.8	62.5
MCF7	3.93	16.5	59.4
BT-549	4.54	>100	>100
HS 578 T	12.7	45.7	>100
MDA-MB-468	13.5	51.3	>100
T-47D	45.6	>100	>100

**Table 2.** MG-MID values and selectivity index for compound **6i** towards the herein examined subpanels.

Subpanel cancer cell line	Compound	
	6i	
	MG-MID	Selectivity index
Leukaemia	68.03	0.30
Colon cancer	18.34	1.11
Non-small cell lung cancer	10.62	1.92
Melanoma	16.8	1.21
CNS cancer	12.70	1.61
Renal cancer	19.19	1.06
Ovarian cancer	16.52	1.23
Breast cancer	15.29	1.33
Prostate cancer	6.63	3.08
Full panel MG-MID	20.45	

Interestingly, the target compound **6i** displayed potent anticancer activity at a single-digit micromolar concentration towards NSCLC (HOP-62, A549/ATCC, HOP-92, NCI-H23, and NCI-H226), CNS cancer (SF-268), Colon cancer (HCT-116), Ovarian cancer (OVCAR-8), Melanoma (LOX IMVI), Prostate cancer (DU-145 and PC-3) Renal cancer (786-0), and Breast cancer (BT-549 and MCF7) cells with GI_50_ spanning in the range: 3.93–9.85 µM as shown in Table 1. In addition, compound **6i** showed good to weak anti-proliferative action against the residual herein examined cell lines with GI_50_ spanning in the interval from 10.10 to 71.00 µM, except for Leukaemia (HL-60(TB), K-562, MOLT-4, and CCRF-CEM) and Melanoma (M14) cancer cell lines which had GI_50_ more than 100 µM. While compound **6i** revealed good to weak cytostatic action towards 41 of the herein examined tumour cell lines with TGI spanning in the range: 16.50–89.10 µM, it had no impact (TGI more than 100 µM) towards Leukaemia (HL-60(TB), CCRF-CEM, MOLT-4, K-562, SR, and RPMI-8226), Colon (HCC-2998, HCT-15, HCT-116, and KM12), CNS (U250), Melanoma (M14 and UACC-257), Ovarian (OVCAR-8), Renal (SN12C and 786-0), and Breast (T-47D and BT-549) cancer cells (Table 1**)**.

Furthermore, the target analogue **6i** was found to be a non-lethal compound (LC_50_ > 100 µM) towards most of the herein examined cancer cells, except for NSCLC (EKVX, HOP-62, HOP-92, NCI-H460, NCI-H522, and NCI-H23), Colon (COLO 205), CNS (SF-539, SNB-19, and SF-295), Melanoma (MALME-3M, LOX IMVI, SK-MEL-28, MDA-MB-435, UACC-62, and SK-MEL-5), Renal (ACHN, CAKI-1, and A498), Ovarian (OVCAR-4), Breast (MDA-MB-231/ATCC and MCF7) and Prostate (PC-3) cancer cell lines with LC_50_ interval: 47.0–96.1 µM (Table 1).

On the other hand, compound **6i** had relatively homologous GI activity towards the cancer screening panel exploring the diverse cancer cells sensitivity, with efficient individual subpanel MG-MID values spanning from 6.63 to 68.03 µM and promising full panel MG-MID equals 20.45 µM. Interestingly, the most vulnerable subpanels to the impact of compound **6i** were Non-Small Cell Lung and Prostate cancers with MG-MID equal 10.62 and 6.63 µM, correspondingly, Table 2.

Moreover, the selectivity index (SI) of the most efficient analogue **6i** was calculated from the division of full-panel MG-MID by subpanel MG-MID. A value greater than 6 implies high selectivity, whereas a ratio from 3 to 6 denotes moderate selectivity. Otherwise, the compound is referred to as non-selective.^24^ Hence, the examined compound **6i** has emerged as a potent non-selective broad-spectrum anti-proliferative agent against all the herein examined subpanels with SI spanning in the interval: 0.3–1.9, except for Prostate cancer cell lines which stood out as a moderately selective agent with SI of 3.08, as depicted in Table 2. Therefore, the outstanding anti-cancer profile of compound **6i** against the herein examined NCI-59 cell panels inspired us to explore its mechanism of action further.

#### Preliminary SAR study of the designed compounds

**Figure 4.**
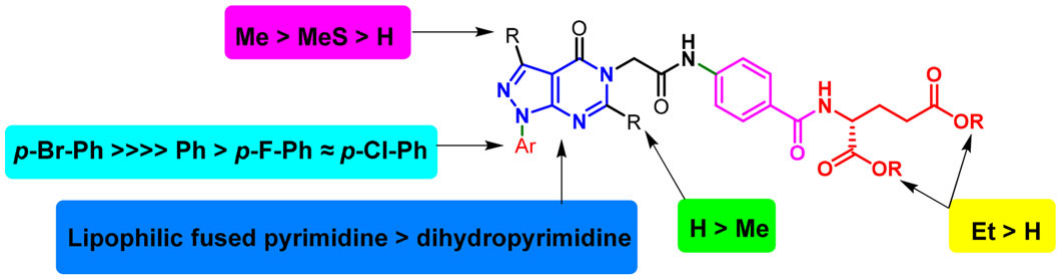
preliminary SAR study of target compounds.

As the tested compounds demonstrated, variable anti-proliferative potencies towards cancer cell lines and structure activity relationships (SAR) about these compounds could be summarised as follows. Firstly, the introduction of the methyl group at C6 of **6a,c,e,g,i** and **7a** (afforded **6b,d,f,h,j** and **7b**) would decrease their anti-proliferative effects while in compound **6k** (afforded **6 l),** the inhibitory activity was increased by 8.18%. Secondly, the inhibitory activity of **6a,b**, **6i,** and **6l** indicated that introducing substitution to the C3, especially methyl group, could significantly increase the inhibitory effects against the cancer cell lines. Thirdly, increasing lipophilicity at N1 by installing a *p-*bromophenyl substituent would significantly increase the inhibitory activity as in **6i**. Fourthly, the inhibitory activity of **7a** and **7b** showed that hydrolysis of carboxylate ester of benzoyl glutamate tail of **6c** and **6d** would not improve the inhibitory effects. In turn, they could moderately decrease the inhibitory activities against most cancer cell lines evidencing that the ester prodrug form is more potent. Fifthly, replacing the pteridine isosteric 6-5 fused system, pyrazolo[3,4-d]pyrimidine, with 1,4-dihydropyrimidine core would significantly increase the inhibitory effect in compounds **9b** and **9c**. Also, the inhibitory effect of **9a, 9b,** and **9c** indicated that installing *p-*bromophenyl substituent at C4 in a pyrimidine ring could significantly increase the inhibitory effects against most of the cancer cells as in **6i**, demonstrating the importance of this moiety on the anti-proliferative activity. Finally, **6i** established better anti-proliferative effects among the tested analogues against NCI-59 cancer cells. In brief, we could conclude that bridging of pyrazolo[3,4-d]pyrimidine core at C5 with diethyl glutamate tail through acetamide linker and installation of a more lipophilic group at N1 and a hydrophobic group at C3 along with no substitution at C6 could potentially impact the anti-proliferative activity (Figure 4).

#### Anti-proliferative activity towards human cancer MCF-7, PC-3, and OVCAR-3 cell lines

The future growth-inhibiting effects of the most active derivative **6i** were investigated towards PC-3 (Prostate cancer), MCF-7 (Breast cancer), OVCAR-3 (Ovarian Cancer), as well as HSF (Normal human skin fibroblast) cells, using MTT assay^25–31^. The cell viability was measured after incubation with the test compound for 48 h, and MTX was co-assayed as a reference antitumor drug. As shown in Table 3, the investigation hinted that compound **6i** exhibited potent growth inhibitory activity, with IC_50_ values of 4.40 ± 0.26, 12.42 ± 0.62, and 11.52 ± 0.58 µM against MCF-7, PC-3, and OVCAR-3, correspondingly. In addition, compound **6i** had about seven-fold, four-fold, and two-fold dwindled efficiency compared to MTX towards MCF-7, PC-3, and OVCAR-3 cancer cells, respectively. Furthermore, the cytotoxicity of **6a** and MTX in normal fibroblast cells (HSF) was also studied, and the selectivity index (SI) was calculated. The results hinted that **6i** elicited stronger cytotoxicity and higher selectivity for cancer than normal cells.

#### DHFR and TS inhibitory activity

To identify the effect on the targeted enzymes, hDHFR and TS inhibition assay was performed on the most potent anticancer analogue **6i**. The assay was performed as reported elsewhere^32–35^ using MTX and 5-FU as reference drugs, respectively. As shown in Table 4, the DHFR inhibition results established that **6i** showed inhibitory potency of an IC_50_ of 2.41 µM compared to MTX (IC_50_ = 0.11 µM). In contrast, the TS inhibition activity was comparable to that of 5-FU (IC_50_ 8.88 and 3.60 μM, respectively).

#### Cell cycle analysis in MCF-7 cells

**Figure 5.**
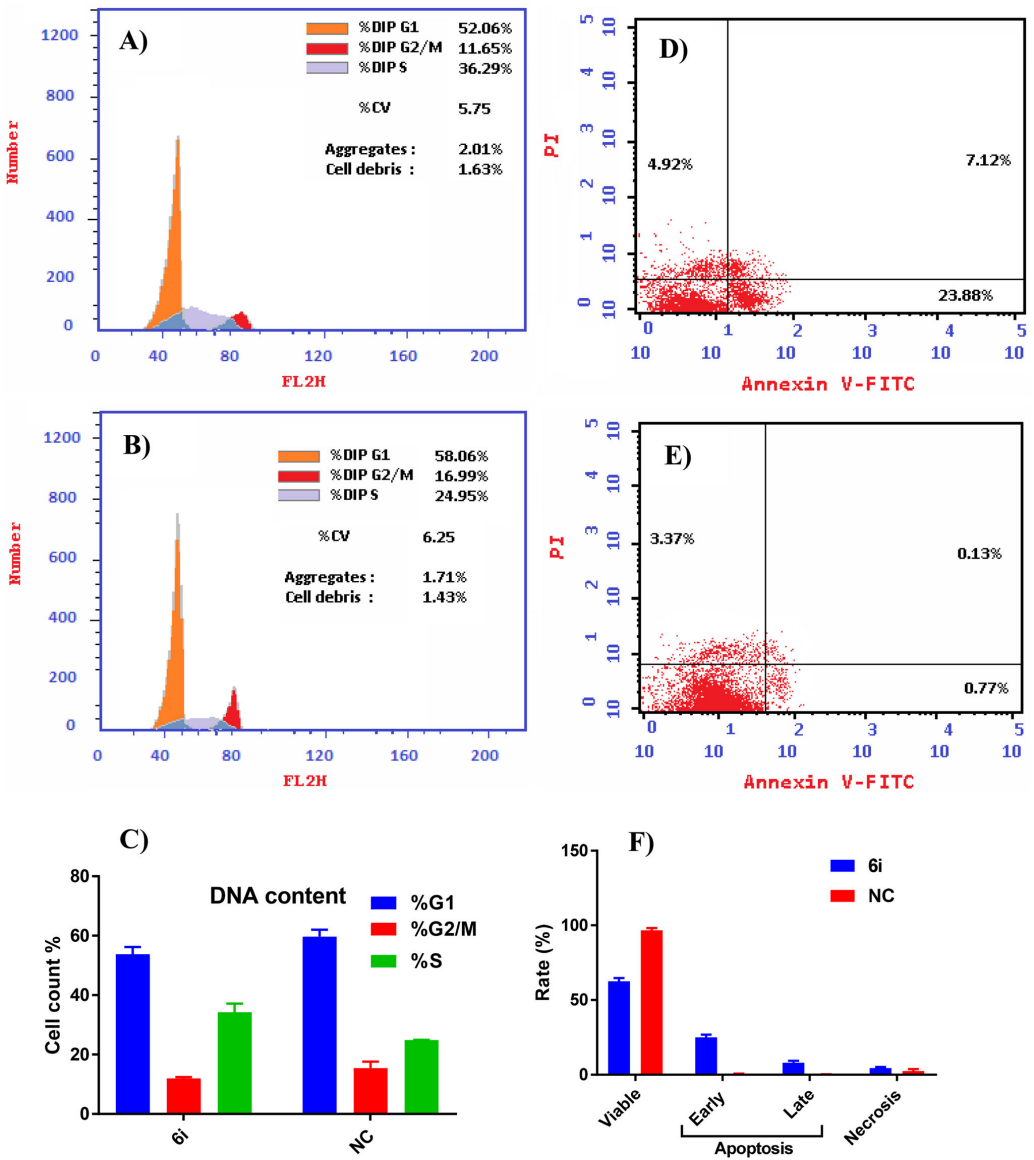
Flow cytometric analyses: cell cycle distribution analysis using PI staining method in which MCF-7 cells were treated by **6i** (10 µM) and DMSO (negative control; NC) for 48 h; (A) **6i**, (B) DMSO, (C) cells percentage in different phases (G0-G1, S, G2/M); apoptotic induction using annexin-V-FITC/PI staining method in which cancer cells were treated by **6i** (10 µM) and DMSO (negative control; NC) for 24 h; (D) **6i**, (E) DMSO, (F) histogram for induction of apoptosis.

The cell cycle distribution modulation and apoptosis induction were investigated to verify the mechanism of action of the most potent compound, **6i**, in MCF-7 cells which were treated with **6i** at 10 µM for 48 h along with an untreated DMSO control, washed with PBS, then fixed with 70% ice-cold ethanol for 12 h followed by staining with propidium iodide (PI), and analysed by flow cytometry^36^^,^^37^. The results showed that the percentage of S-phase cells increased to 36.29% for **6i** from 24.95% (DMSO control) (Figure 5(A–C)). These results indicated that **6i** could effectively induce S-phase arrest.

#### Analysis of apoptosis

Again, MCF-7 cells were treated with **6i** (10 µM) to assess its effect on cell apoptosis. As shown in Figure 5(D,E), the fraction of cells in early apoptosis were 26.27% and 0.43%, while in late apoptosis, they were 8.90% and 0.29% for compound **6i** and DMSO control, respectively. The obtained results hinted that compound **6i** could induce apoptotic pathways. However, the ratio of apoptosis inferred that the anti-proliferative effect of compound **6i** was largely dependent on apoptosis.

## *In silico* molecular modelling studies

Molecular modelling analyses were accomplished by OpenEye® Scientific software (ver. 2021.2.1) to investigate the docking mode of the most active analogue **6i** inside the active site of both hDHFR (PDB ID: 1U72) and TS (PDB ID: 1JU6)^19^^,^^38–43^. The reference drugs, MTX and PMX, were re-docked using the same procedure as compound **6i** to validate the docking results and rationalise the predictive protein-ligand interactions within the active site^18^^,^^44–46^. As shown in Figures S36 and S37 (in Supplementary Material), the docking pose of the co-crystallised ligand showing interactions similar to those existing in the crystal structure, and the hydrophobic interactions were roughly the same.

**Figure 6.**
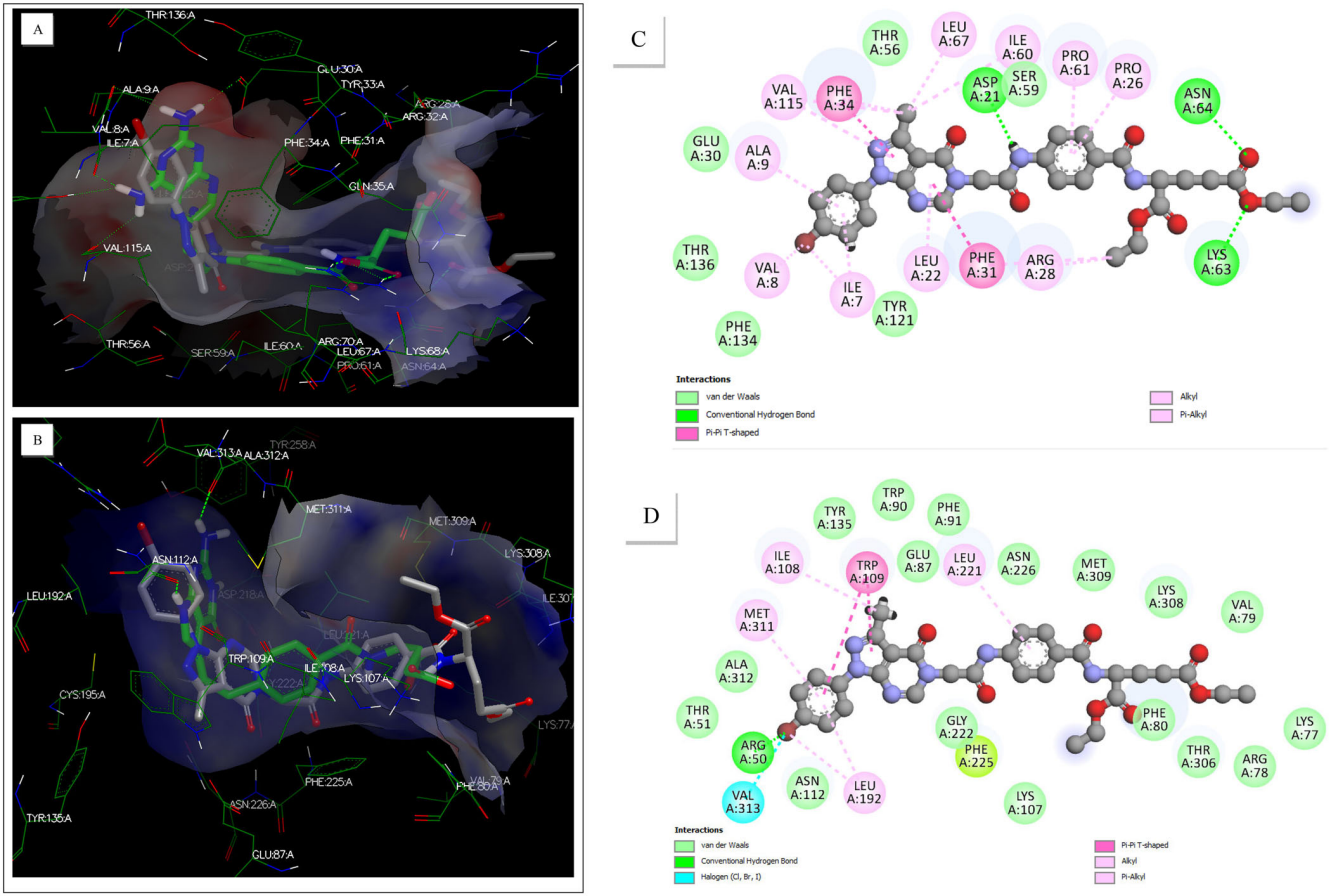
Binding modes to the active sites of DHFR and TS enzymes: (A) 3D representation of **6i** displaying an overlay with the crystal structure of MTX in the active site of DHFR; (B) 3D representation of **6i** demonstrating an overlay with the crystal structure of PMX in the active site of TS; (C) 2D diagram of **6i** in DHFR active site; (D) 2D diagram of **6i** in TS active site.

As seen in Figure 6(A,B), the docked pose of compound **6i** (grey) showed an overlay with the cocrystallised ligand MTX (green) in the hDHFR active site^47^. The results display that the pyrazolo[3,4-d]pyrimidine core of **6i** displayed only hydrophobic interactions: alkyl, pi-pi T-shaped, and pi-alkyl. In contrast, there are three hydrogen bonds for MTX in the corresponding binding site. The NH of the acetamide bridge showed a hydrogen bond with Asp21, while the benzoyl moiety demonstrated pi-alkyl hydrophobic interactions with Pro61:A and Pro26:A residues. The benzoylglutamate tail of **6i** formed two hydrogen bonds from the γ-carboxylate group with Asn64:A and Lys63:A residues, which differ from MTX′s. Also, compound **6i** showed a FRED chemgauss4 score of −5.06, while MTX had a value of −13.38. The overlay of the docked pose of **6i** with the crystal structure of MTX indicated that **6i** established, to some extent, a similar binding mode with that of MTX, especially the formation of tortuous “L” structure, which allows tightly fitting in the active site of DHFR. This offered a plausible rationale for why **6i** showed the best anti-cancer profile among the series tested, as established by NCI screening and molecular mechanisms. Furthermore, the molecular docking results infer that the hydrophobic interactions or hydrogen bonds of **6i** were weaker than MTX′s. Consequently, **6i** could be an inhibitor of DHFR but should be less potent than MTX. Overall, the Fred docking results proposed modifications or extensions on the N7 and benzoyl moiety as they projected towards the protein cavity.

Figure 6(C,D) also reveals an overlay of the docked pose of compound **6i** (grey) with the crystal structure of PMX (green) in the human TS active site^48^. Like that of human DHFR, the TS active site consists of four regions: the pteridine, the bridge, the benzoyl, and the glutamate regions. The results display that the pyrazolo[3,4-d]pyrimidine core forms a hydrogen bond between bromine and Arg50:A and hydrophobic interactions: pi-pi T-shaped, alkyl, and pi-alkyl. The acetamide linker allows an overlay with PMX crystal structure through tortuous “L” structure formation. The benzoyl moiety formed mixed pi-alkyl hydrophobic interactions with Leu221:A. Finally, the glutamate tail of **6i** forms van der Waals attractions in the benzoylglutamate region. The binding pose of **6i** indicates that the pyrazolo[3,4-d]pyrimidine motif and benzoylglutamate tail form a tortuous “L” structure through the acetamide bridge region and spatially stacked well with the docked pose of PMX. Furthermore, the modelling study implies that **6i** could be an inhibitor of TS but should be less potent than PMX, which is aligned with the results of inhibitory enzyme assays. Overall, the Fred docking results proposed modifications or extensions on the N1, N7, benzoyl moiety, and γ-carboxylic ester group as they project towards the protein cavity.

## Drug-likeness and ADMET prediction

Drug-likeness and pharmacokinetics ADMET properties for the most active compound **6i** were calculated using ADMETlab 2.0 predictor^49^^,^^50^. As illustrated in Table 5, Only one violation from Lipinski’s rule of five is permitted for a compound to obey the rule. It can be observed that compound **6i** revealed two violations from Lipinski’s rule while Pfizer accepts **6i** criteria as a good prodrug candidate. The ADMET prediction data of compound **6i** are cited in Table 6. The results revealed that **6i** has a good synthetic accessibility score (SA score 3.14), a high GIT absorption rate since the human intestinal absorption (HIA) is 90% or more, and moderate solubility in water that helps in solubilisation in GIT fluids before absorption process as indicated by the estimated log S value (−5.07). In addition, the tested compound **6i** demonstrated more than 20% bioavailability, as indicated by the F_20%_ result, while the plasma protein binding was 91.72%. Also, it is expected to be a non-inhibitor of CYP1A2, CYP2C19, and CYP2D6 enzymes, suggesting a low probability of causing drug-drug interaction. Furthermore, carcinogenicity and acute toxicity tests showed that compound **6i** is neither carcinogenic nor toxic, evidencing the good ADMET profile.

**Table 3.** Growth inhibitory activity of compound **6i** against MCF-7, PC-3, OVCAR-3, and HSF cells.

Comp.	IC_50_ (µM)^a^				Selectivity index		
	MCF-7	PC-3	OVCAR-3	HSF	MCF-7	PC-3	OVCAR-3
**6i** MTX	4.40 ± 0.26	12.42 ± 0.62	11.52 ± 0.58	48.52 ± 2.43	11.02	3.91	4.21
	0.58 ± 0.03	2.72 ± 0.14	6.31 ± 0.32	13.68 ± 0.69	23.71	5.03	2.16

^a^Values are the mean ± SD from triplicates.

**Table 4.** DHFR and TS inhibitory activity of compound **6i** and reference drugs.

	IC_50_ (µM)	
	DHFR	TS
**6i**	2.41 ± 0.13	8.88 ± 0.46
MTX	0.11 ± 0.01	–
5-FU	–	3.60 ± 0.19

*Note:* ± represents the standard deviation from triplicates.

**Table 5.** Lipinski’s rule of five calculated parameters for the most active anti-cancer compound **6i**.

Parameter							Pfizer rule
Log P^a^	TPSA^b^	MW^c^	*n*HBD^d^	*n*HBA^e^	*n*RB^f^	Log S^g^	
3.32	163.51	666.14	2	13	16	−5.07	Accepted

^a^Calculated lipophilicity.

^b^Total polar surface area.

^c^Molecular weight.

^d^Hydrogen bond donors.

^e^Hydrogen bond acceptors.

^f^Rotatable bonds.

^g^Log of the aqueous solubility.

**Table 6.** Predicted ADMET properties for the most active anti-cancer compound **6i**.

SAscore	HIA%	F_20%_	PPB	CYP1A2 CYP2C19 CYP2D6 inhibition	CL	Carcinogenicity	Acute toxicity rule
3.14	≥90%	≥20%	91.72%	Non-inhibitor	2.47	Noncarcinogenic	Non-toxic

## Experimental part

### Chemistry

Bruker Avance III 400 MHz HD FT-high resolution-NMR spectrometer was used for recording the spectra of ^1^H and ^13^C NMR in deuterated solvents DMSO-*d_6_* and CDCl_3_ using the residual peaks at δH = 2.50 ppm and δH = 7.27 ppm as an internal standard for ^1^H NMR spectra. In contrast, δC = 39.51 ppm and δC = 77.23 ppm were used for ^13^C NMR spectra, respectively. The multiplicity of signals was verified as singlet (s), doublet (d), doublet of doublets (dd), triplet (t), quartette (q), multiplet (m), or broad (br). The *J*-coupling constants (in Hz) were recorded. For reaction monitoring, silica gel 60 F_254_ pre-coated plates (Merck) were used for TLC using UV light (254 nm) for visualisation. The purification processes of the prepared analogues were accomplished by either recrystallisation from an appropriate solvent or chromatographic separation utilising silica gel for column (150–250 μm). All melting points were determined on an electro thermal melting point apparatus (Stuart Scientific, Model SMP1, UK) and were uncorrected. Mass spectrum was carried out on Direct Inlet part to mass analyser in Thermo Scientific GC-MS model ISQ (Waltham, MA, USA). Vario elemental analyser III (Vario, Germany) was used for analysing C, H, and N for all designed analogues. The findings agreed with the proposed structures within ±0.4% of the theoretical values. Dry solvents and chemicals were purchased from commercial suppliers and used as received. Compounds **2a–f**^18^^,^^19^^,^^51–53^, **3a–e**^18^^,^^19^^,^^51^^,^^54–58^, **3g–l**^18^^,^^19^^,^^51^^,^^54–58^, **5**^59^ and **8a–c**^60–64^ were synthesised as described.

#### General procedure for synthesis of compounds 3a–l

To a suspension of 1-aryl-5-amino-4-cyanopyrazoles **2a–f** (0.006 mol) in aliphatic acid (18 ml), POCl_3_ (1.2 ml) was added instantaneously. The mixture was refluxed for 2 h^20^ and then cooled to 0 °C, poured slowly to crushed ice, filtered, and recrystallised from formic acid (85%) to provide the intermediates **3a–l** in good yields.

#### General procedure for the synthesis of N5 appended analogues 6a–l

To a solution of pyrazolo[3,4-d]pyrimidinone derivatives **3a–l** (0.001 mol) in DMF (2 ml), diethyl (4-(2-chloroacetamido)benzoyl)glutamate **5** (0.3988 g, 0.001 mol), anhydrous K_2_CO_3_ (0.1382 g, 0.001 mol), and sodium iodide (0.015 g, 0.0005 mol) were added. The reaction was heated at 65° for 1–2 h as monitored by TLC^65^. The reaction mixture was cooled to 25 °C and then poured dropwise onto crushed ice containing brine (5 ml). Finally, the formed product was filtered and crystallised from the appropriate solvent to provide the N5-derived analogues **6a–l**.

#### General procedure for the synthesis of acid derivatives (7a,b)

A suspension of **6c,d** (0.002 mol) in 5 ml aqueous sodium hydroxide (1 mol/l) was stirred at 25 °C for 2 h (TLC monitoring)^3^. The reaction mixture was then filtered and acidified carefully with 0.5 mol/l HCl. The formed product was filtered and crystallised from the appropriate solvent to give **7a,b**.

#### General procedure for preparation of 1,4-dihydropyrimidine derivatives (9a–c)

To a stirred solution of 2-thioxo-1,4-dihydropyrimidine intermediates **8a–c** (0.001 mol) in DMF (2 ml), diethyl (4-(2-chloroacetamido)benzoyl)glutamate **5** (0.3988 g, 0.001 mol), anhydrous potassium carbonate (0.138 g, 0.001 mol), and sodium iodide (0.015 g, 0.0005 mol) were added. The reaction mixture was stirred at room temperature for 8 h, as indicated by TLC^65^. The reaction mixture was then poured dropwise onto crushed ice containing 5 ml of brine. The formed precipitate was filtered and crystallised from the appropriate solvent to yield the desired analogues **9a–c**.

The key intermediate (**3f**) and target compounds (**6a–l, 7a,b,** and **9a–c**) were fully characterised in the Supplementary Material.

### Biological activity

#### NCI-60 screening methodology

The designed analogues were submitted to NCI-Chemotherapeutic Agents Repository (MD, USA) for screening on a panel of 59 cancer cell lines. All compounds were initially examined at 10 µM concentration (Single-Dose Screen), and then the most efficient analogue **6i** was screened further at 0.01–100 µM (Five-Dose Screen). The screening procedures were performed according to NCI-Drug Evaluation Branch protocol using SRB protein assay, as described previously^21^^,^^66^. The experimental procedures were detailed in the Supporting Information.

#### MTT cytotoxicity assay

The MTT assay was carried out according to the procedure cited earlier^25–31^. Briefly, in a 96-well plate, either cancer or normal cells were seeded (10^6^ cells/cm^2^; 100 µL/well) and incubated for 12 h at 37 °C and CO_2_ (5%), then serial dilutions of compound **6i** were added and incubated for 48 h using DMSO control (0.5% V/V). MTT (3-(4,5-dimethylthiazoyl)-2,5-diphenyl-tetrazolium bromide; Cat. No. M-5655) 5 mg/mL was then added. After 4 h incubation, the formed MTT formazan crystals were solubilised by 10% Triton X-100 plus 0.1 N HCl in anhydrous isopropanol. The absorbance was read using a Bioline plate reader at λ_570–630 _nm^25–29^. The selectivity of **6i** towards HSF cells was also determined as follows^30^^,^^31^:

$$SI=\frac{{\text{IC}}_{50}\text{HSF}\;\text{cells}}{{\text{IC}}_{50}\text{of}\;\text{cancer}\;\text{cells}}$$

#### Cell cycle analysis

The cell cycle assay was carried out according to the previously reported^36^^,^^37^. Briefly, in a six-well plate, 2 ml of MCF-7 cells were seeded per well and incubated overnight at 37 °C and CO_2_ (5%). After 12 h incubation with serum-free medium, they were treated with 10 μM of **6i**, incubated for 48 h, then washed twice with 1× PBS, trypsinised and centrifuged for 5 min (500 xg) at 4 °C. And then the cell pellet, in ice-cold 1× PBS, was fixed with absolute ethanol at −20 °C for 2 h, washed with 1× PBS ice-cold solution and finally stained with propidium iodide (PI)/RNase (Abcam, ab139418) for 0.5 h at 25 °C in the dark. flow cytometry determined the DNA content in cell phases using BD FACS Calibur^36^^,^^37^.

#### Apoptosis analysis

The MCF-7 cells (10^6^ cells/ml) were treated with either 10 µM of **6i** or DMSO (NC) for 24 h. After that, the cells were trypsinised, collected, washed, and stained with PE-Annexin-V (BioVision, K101-25) in the presence of PI^67^. The induction of apoptosis was analysed by flow cytometry using BD FACS Calibur^67^.

#### DHFR inhibitory assay

DHFR inhibition assay kit (BioVision, K247–100) supplied with recombinant hDHFR was utilised in this assay. The assay was performed as instructed in assay kit^32^. Briefly, in a 96-well clear plate, serial dilutions of the test compound (2 μL) were loaded. DHFR was diluted in assay buffer (400-fold), and 98 μL was added to the assigned wells for compound **6i** and enzyme control. An assay buffer (100 µl) was used as a Background Control. Then, 40 μL of diluted NADPH in assay buffer (40-fold dilution) was added and mixed well. At 25 °C in the dark, the plate was incubated for 15 min. After that, the diluted substrate in assay buffer at 15-fold dilution (60 μL) was added to assigned wells. Instantaneously, the absorbance was read at 340 nm for 10–20 min at 25 °C in kinetic mode by Robonik ELISA plate reader. Finally, the activity and relative inhibition percentages were measured using the equations indicated in the assay kit^33–35^.

#### TS inhibitory assay

The assay was performed as instructed in the assay kit (ProFoldin, TMK100KE, MA, USA**)**. Briefly, in a standard black well plate (Matrix 4318), water (12 µl), 10 × Buffer (3 µl), 10 × dTMP (3 µl), 10 × ATP (3 µl) and 10 × kinase (3 µl) were mixed well. After 2 min, 10 × MUK Reagent A (3 µl) and 10 × MUK Reagent B (3 µl). The mixture was then incubated at 37 °C for 1 h, and 1 × fluorescence dye (30 µl) was added. Finally, the fluorescence intensity was read at 535 nm with excitation at 485 nm utilising a Robonik ELISA plate reader.

#### Drug-likeness and ADMET analyses

Drug-likeness, physicochemical and pharmacokinetic characters of the most potent analogue **6i** were predicted using an accurate, comprehensive, and efficient web-based tool ADMETlab 2.0. They were utilising a high-quality database and a framework for multi-task graph attention^49^^,^^50^.

#### Molecular modelling studies

The straightforward OpenEye® scientific software (2021.2.1) was used for molecular docking studies^19^^,^^38–42^. A library of the most potent compound **6i**, MTX, and PMX was built, and MMFF94 force field was used for energy minimisation then generation of multi-conformers was offered by OMEGA^®^ module^68^. Both hDHFR (PDB ID 1U72) and TS (PDB ID 1JU6) receptors were prepared by the Spruce^®^ application, and then FRED^®^ module was utilised for molecular modelling, and hence FRED Chemgauss4 score generation^69^. The Vida^®^ module was used to visualise the tested compounds′ poses to both DHFR and TS active sites^70^. Also, a 2D diagram of binding interactions was generated by the Discovery Studio Visualisation tool (DS)^71^^,^^72^. The Docking-Report module generated the docking report through the command lines stated in OpenEye documentation^73^.

## Conclusion

In this research, a novel series of multifunctional pyrazolo[3,4-d]pyrimidine and 1,4-dihydropyrimidine Glu-based derivatives **6a–l**, **7a,b,** and **9a–c** were designed and synthesised as potential antifolate agents. The NCI-59 Screening assay displayed that compounds **6i** and **9a–c** showed the best MGI% among the series investigated. At the NCI five-dose assay, compound **6i** exhibited potent anti-proliferative activity against the herein tumour screening panel with GI_50_ spanning in the range: 3.93–9.85 µM. Compound **6i** revealed good to weak cytostatic activity with TGI spanning in the range: 16.50–89.10 µM, and it was found to be a non-lethal compound (LC_50_ > 100 µM) towards most of the herein examined cancer cell lines. Moreover, compound **6i** elicited broad-spectrum anticancer activity with effective subpanel MG-MID interval: 6.63–68.03 µM and promising full panel MG-MID of 20.45 µM with moderate selectivity towards Prostate cancer cell lines. The target analogue **6i**, as the most potent derivative, demonstrated high selectivity towards cancer cell lines with IC_50_ values equal to 4.40, 12.42, and 11.50 against MCF-7, PC-3, and OVCAR-3 cells, respectively. Furthermore, compound **6i** showed potent hDHFR inhibitory activity with an IC_50_ equal to 2.41 µM compared to MTX (IC_50_ = 0.11 µM), while the inhibitory activity towards TS was 8.88 µM compared to 5-FU (IC_50_ = 3.6 µM). The target compound **6i** probably acts through dual inhibition of DHFR and TS enzymes. Compound **6i** could significantly arrest the cells at the S-phase of the cell cycle and potentially induce apoptosis. The *in silico* molecular docking studies implied that compound **6i** had an overlay with the crystal structure of MTX and PMX in their active sites of DHFR and TS, respectively, forming a tortuous “L” structure through the acetamide bridge region. The drug-likeness and ADMET analysis revealed that **6i** met the Pfizer acceptance criteria. These results thoroughly delineate **6i** as a good lead compound and multi-targeted antifolate agent for further study.

## Supplementary Material

Supplemental Material
