# Supplementary material for: Novel Glu-based pyrazolo[3,4-d]pyrimidine analogues: design, synthesis and biological evaluation as DHFR and TS dual inhibitors
Source: J Enzyme Inhib Med Chem. 2023 Apr 20;38(1):2203879. doi: 10.1080/14756366.2023.2203879 (PMC10120551; doi:10.1080/14756366.2023.2203879)
Supplement: Supplemental Material [file IENZ_A_2203879_SM1422.pdf]

Contents

1 NMR spectra 1

1.1 3f 1

1.2 6a 2

1.3 6b 3

1.4 6c 4

1.5 6d 5

1.6 6e 6

1.7 6f 7

1.8 6g 9

1.9 6h 10

1.10 6i 11

1.11 6j 12

1.12 6k 13

1.13 6l 14

1.14 7a 15

1.15 7b 16

1.16 9a 17

1.17 9b 18

1.18 9c 19

2 3D representation of MTX and PMX inside the active sites 20

3 NCI-60 Cell line screening; One-Dose Screen 22

3.1 6a 22

3.2 6b 23

3.3 6c 24

3.4 6d 25

3.5 6e 26

3.6 6f 27

3.7 6g 28

3.8 6h 29

3.9 6i 30

3.10 6j 31

3.11 6k 32

3.12 6l 32

3.13 7a 34

3.14 7b 35

3.15 9a 36

3.16 9b 37

3.17 9c 38

4 NCI-60 cell line screening; Five-Dose Screen 38
5 DHFR assay graphs for both 6i and MTX 42
6 TS assay graph for both 6i and 5-FU 43
7 Experimental Chemistry 43
8 NCI-60 Screening Methodology 48
8.1 NCI 60 Cell One-Dose Screen 48
8.2 NCI 60 Cell Five-Dose Screen 48

1 NMR spectra

1.1 3f

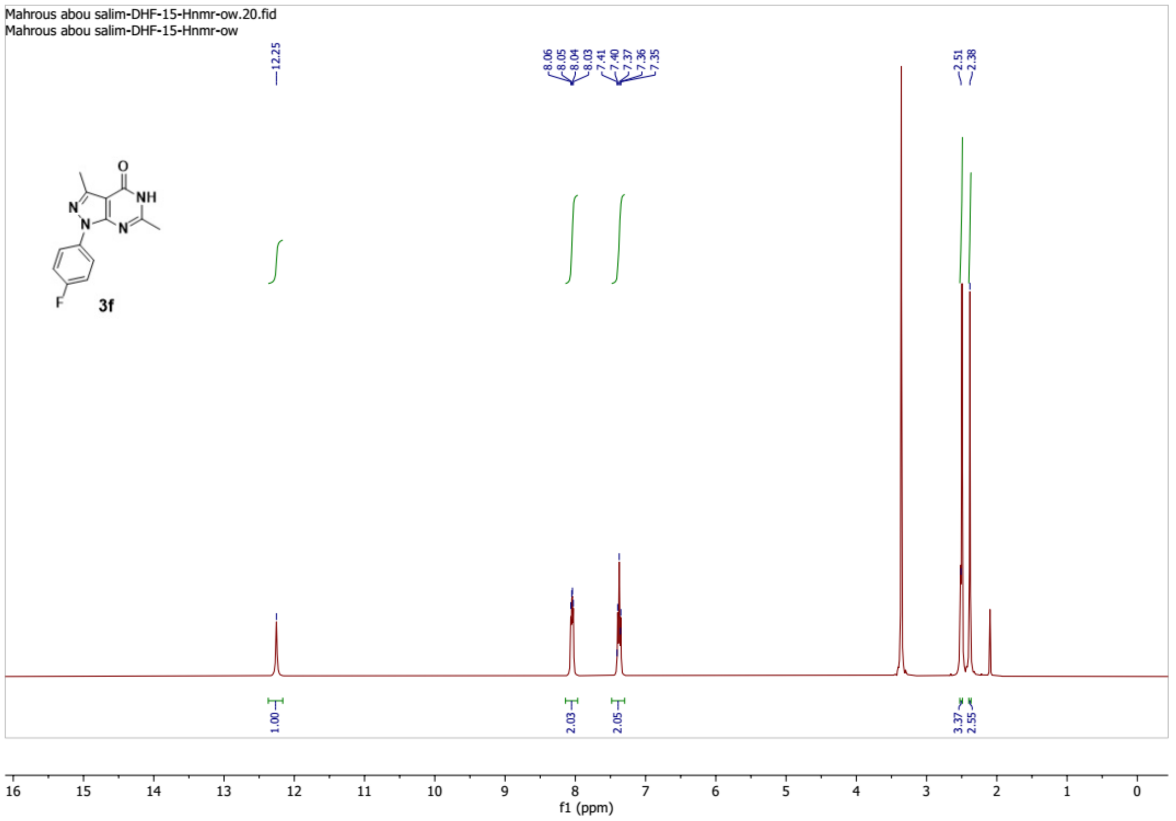

Figure S 1. ^{1}H NMR of **3f**.

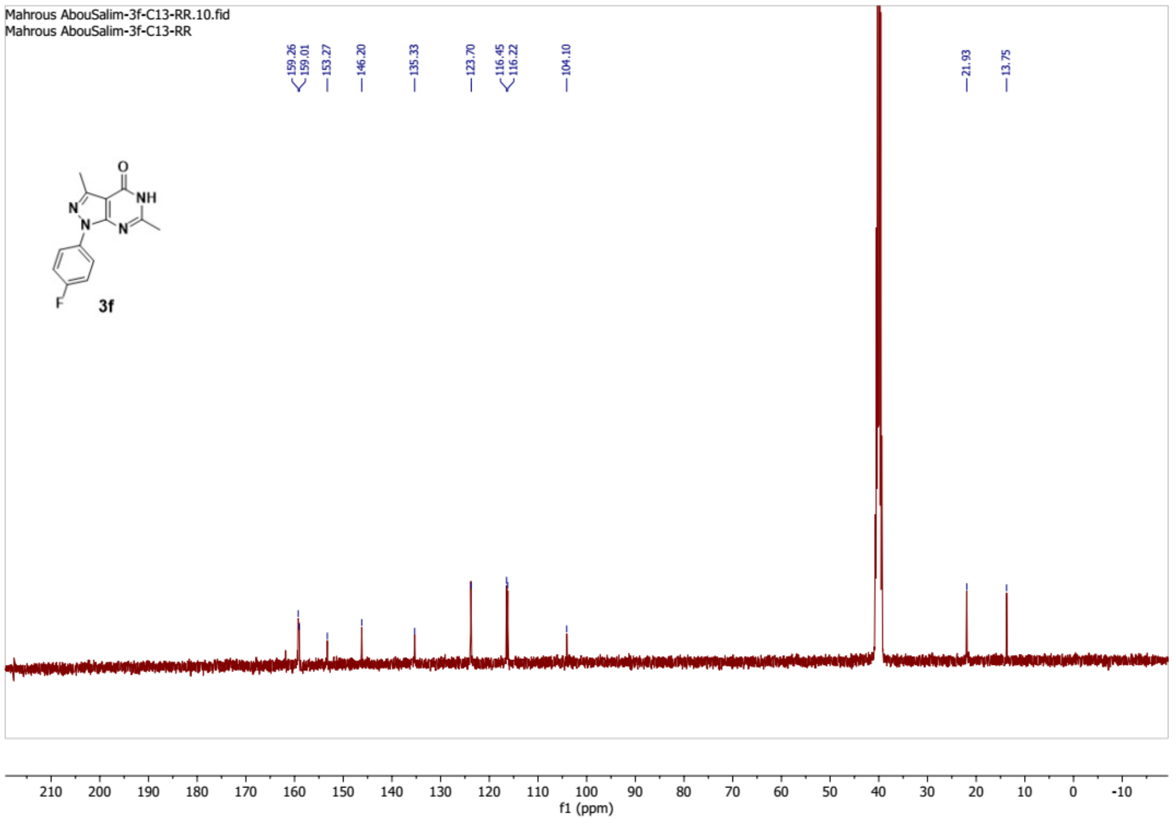

Figure S 2. ^{13}C NMR of **3f**.

1.2 6a

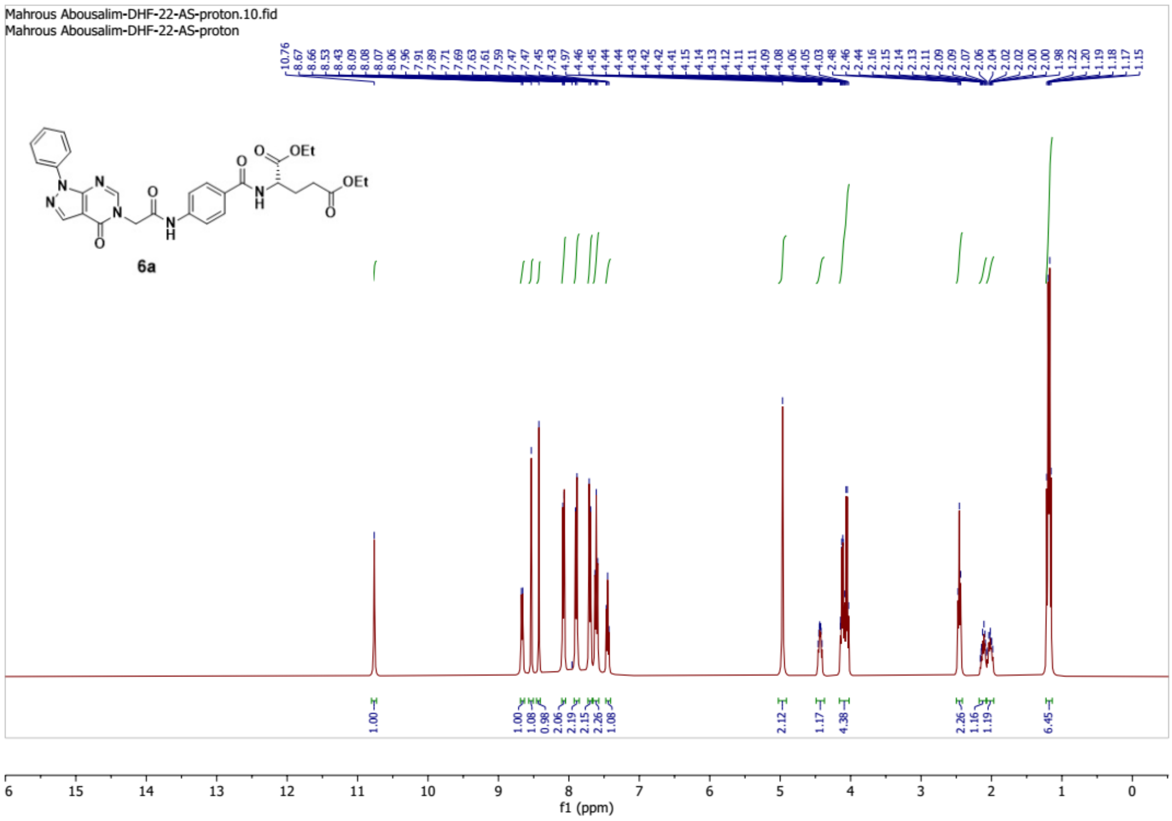

Figure S 3. ^{1}H NMR of **6a**.

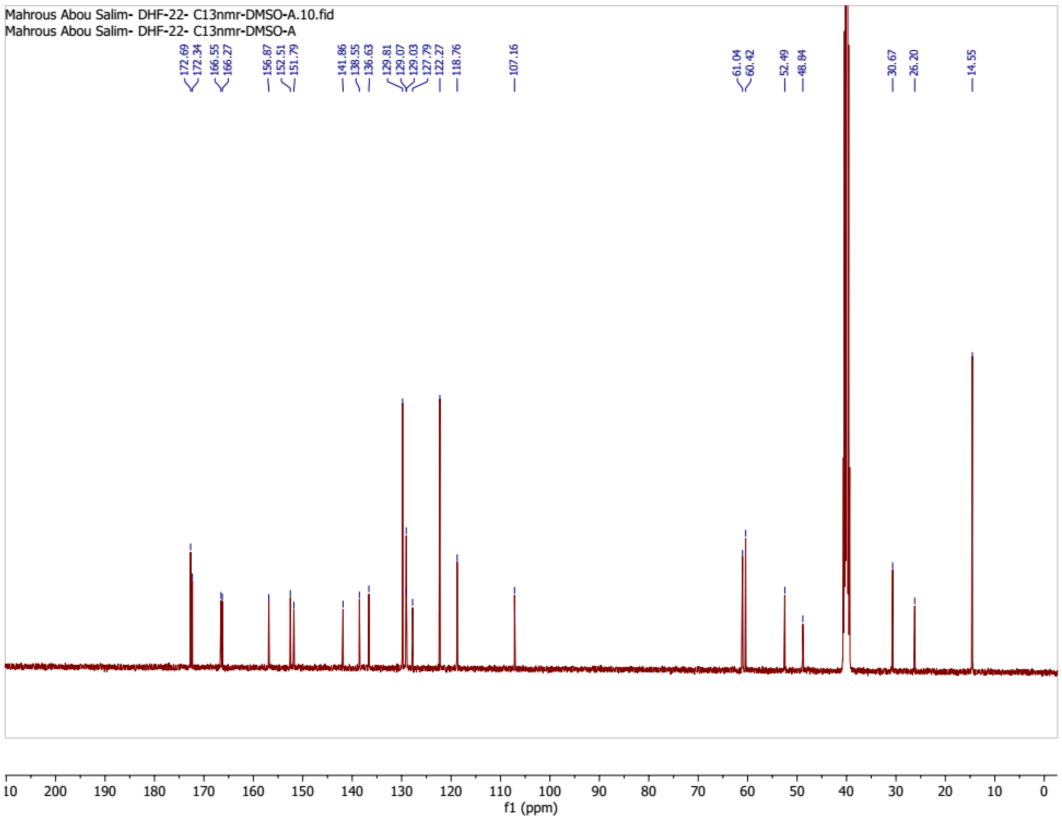

Figure S 4. ^{13}C NMR of **6a**.

1.3 6b

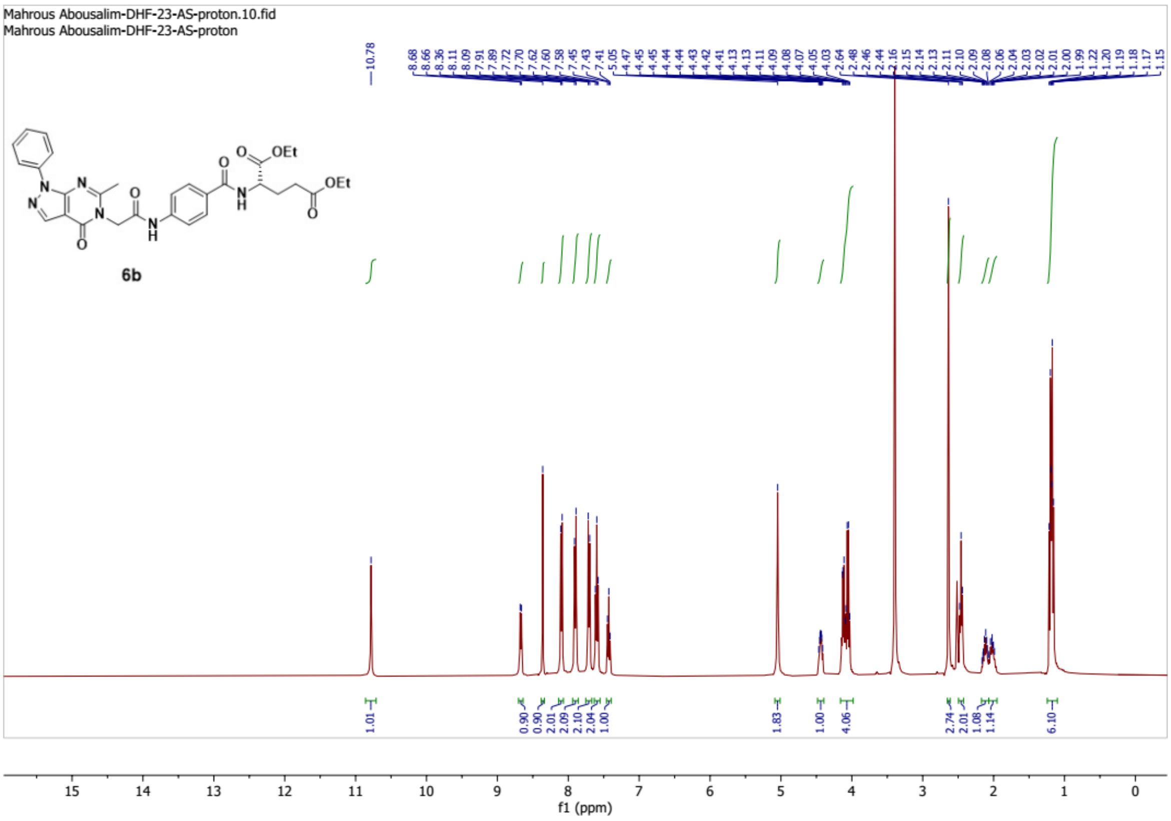

Figure S 5. ^{1}H NMR of **6b.**

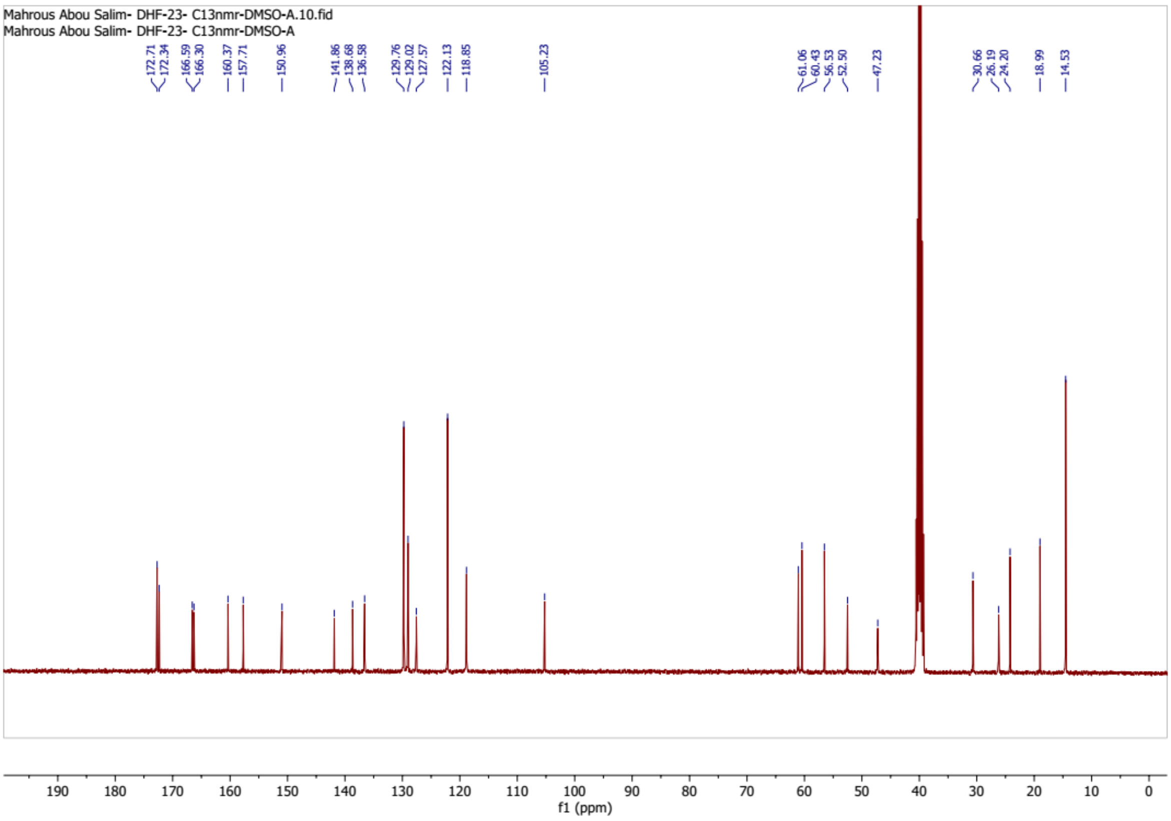

Figure S 6. ^{13}C NMR of **6b**

1.4 6c

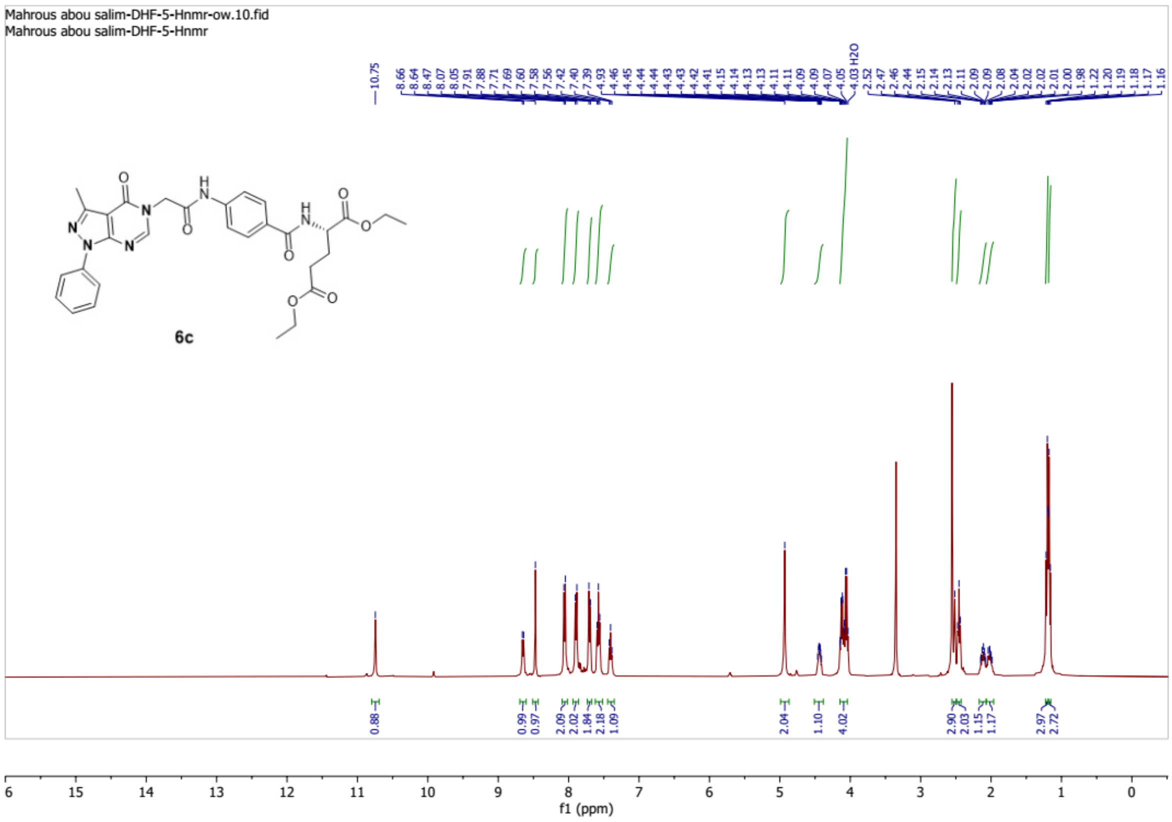

Figure S 7. ^{1}H NMR of **6c.**

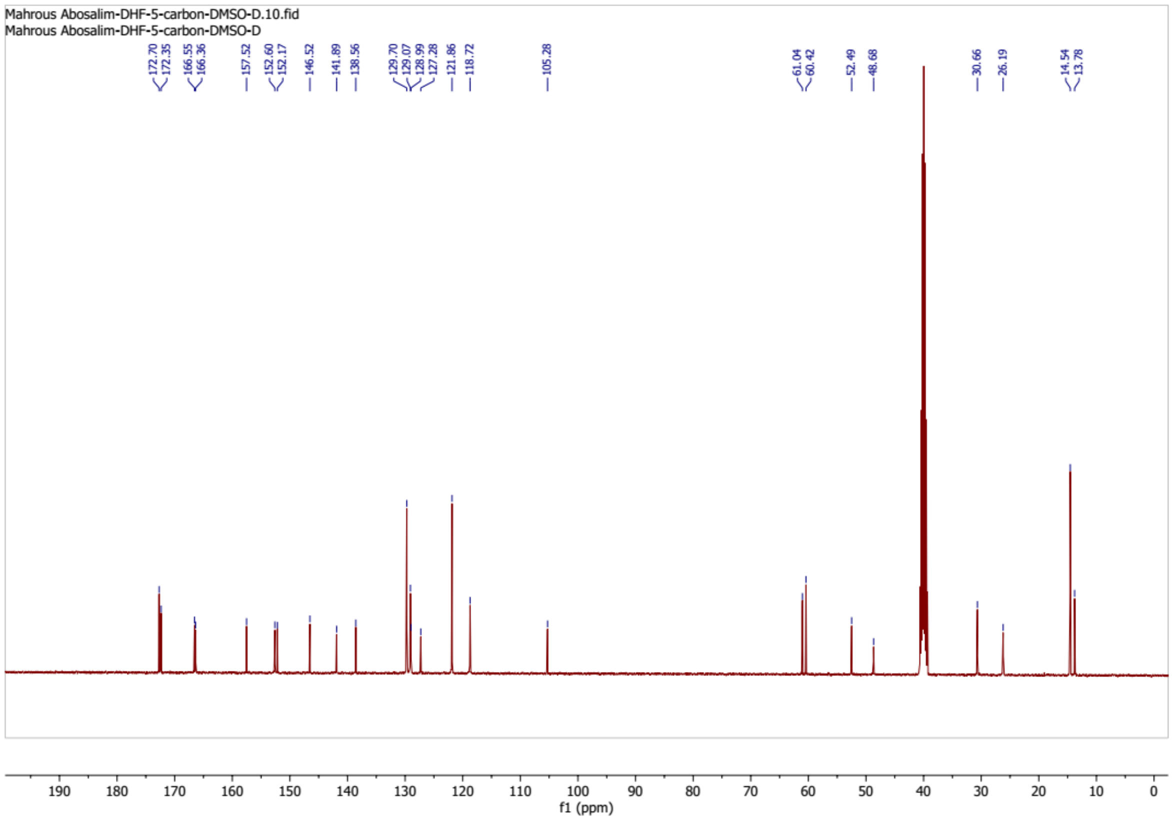

Figure S 8. ^{13}C NMR of **6c.**

1.5 6d

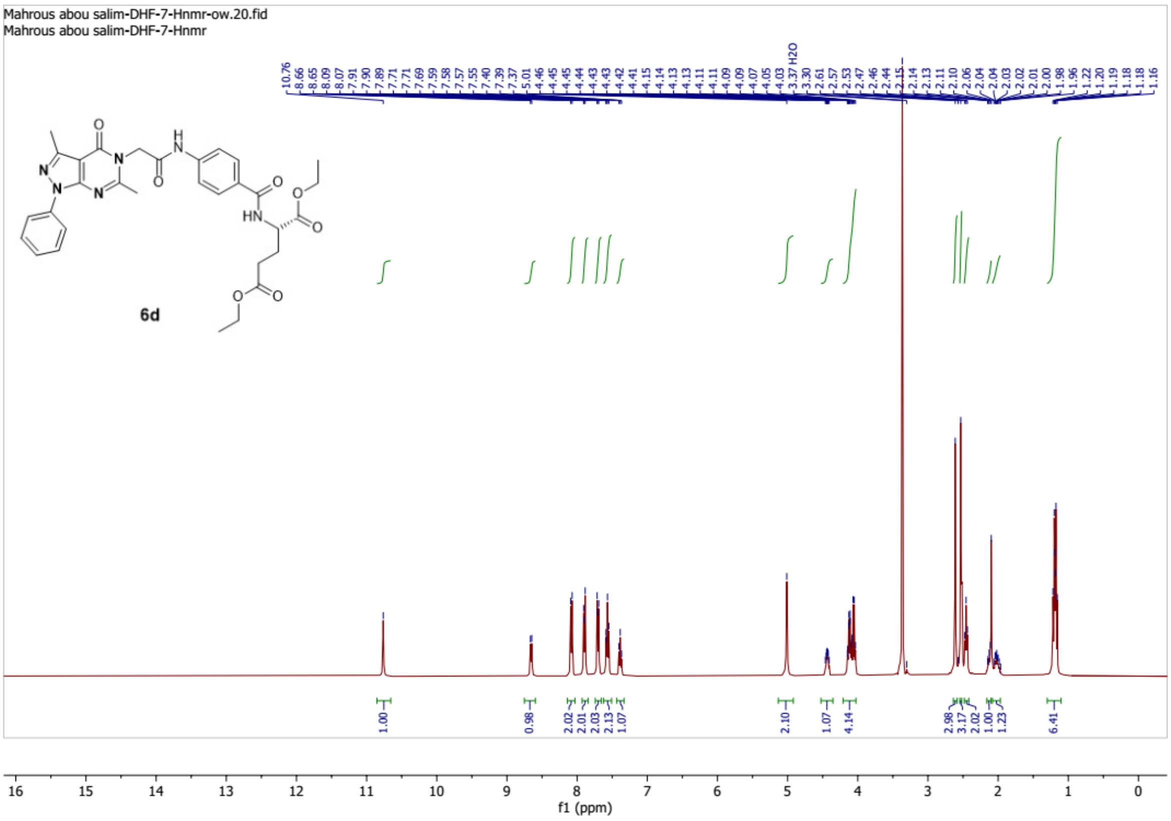

Figure S 9. ^{1}H NMR of **6d**

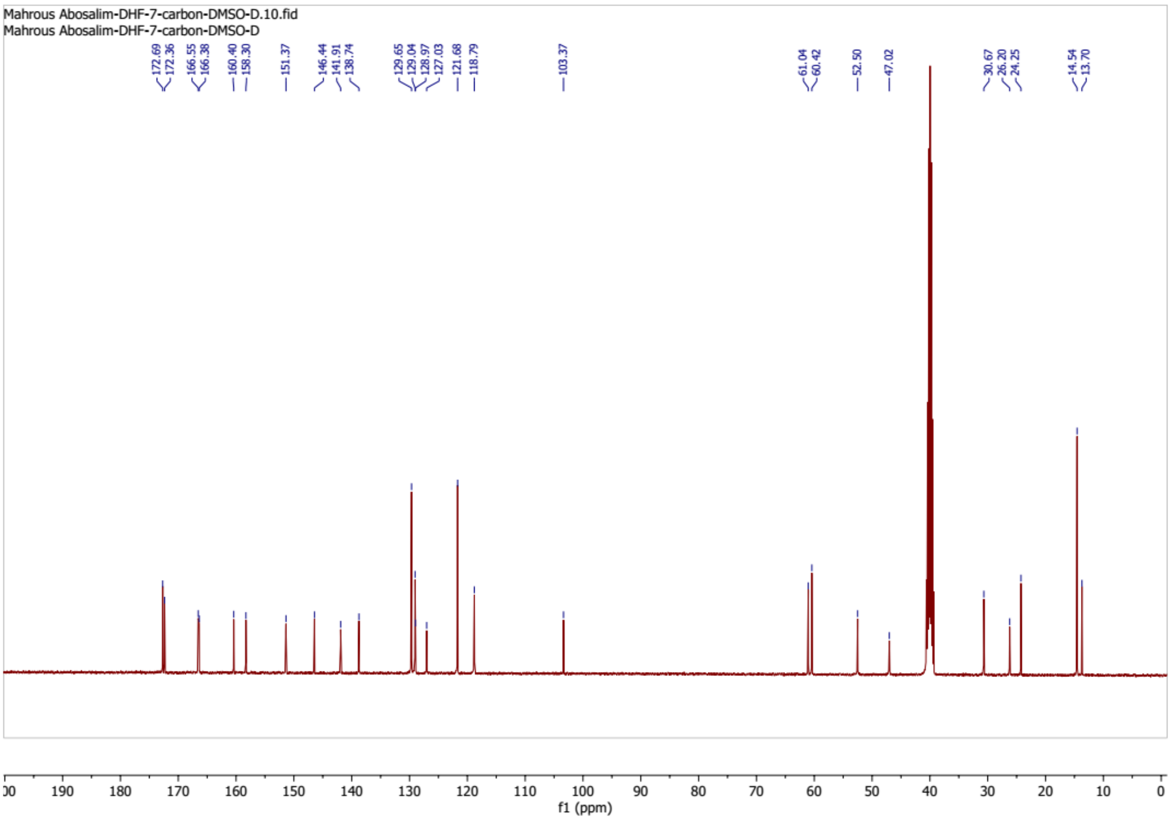

Figure S 10. ^{13}C NMR of **6d.**

1.6 6e

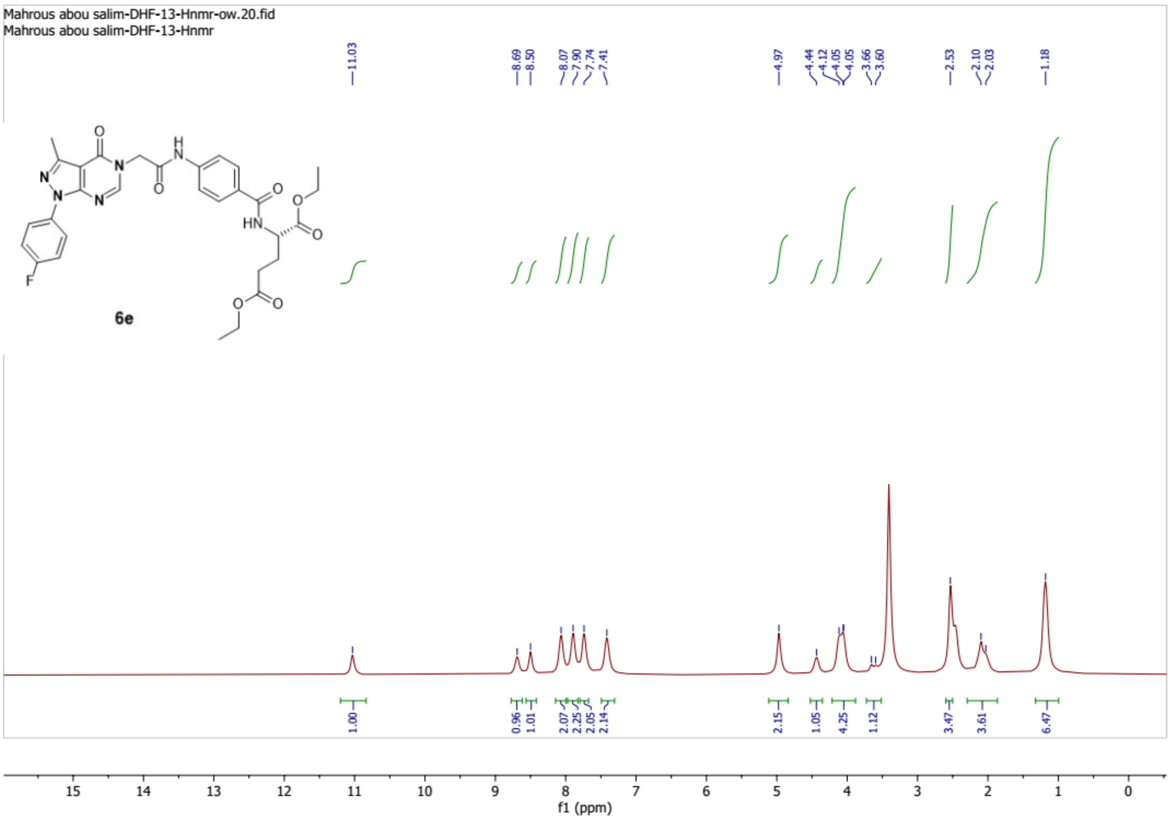

Figure S 11. ^{1}H NMR of **6e.**

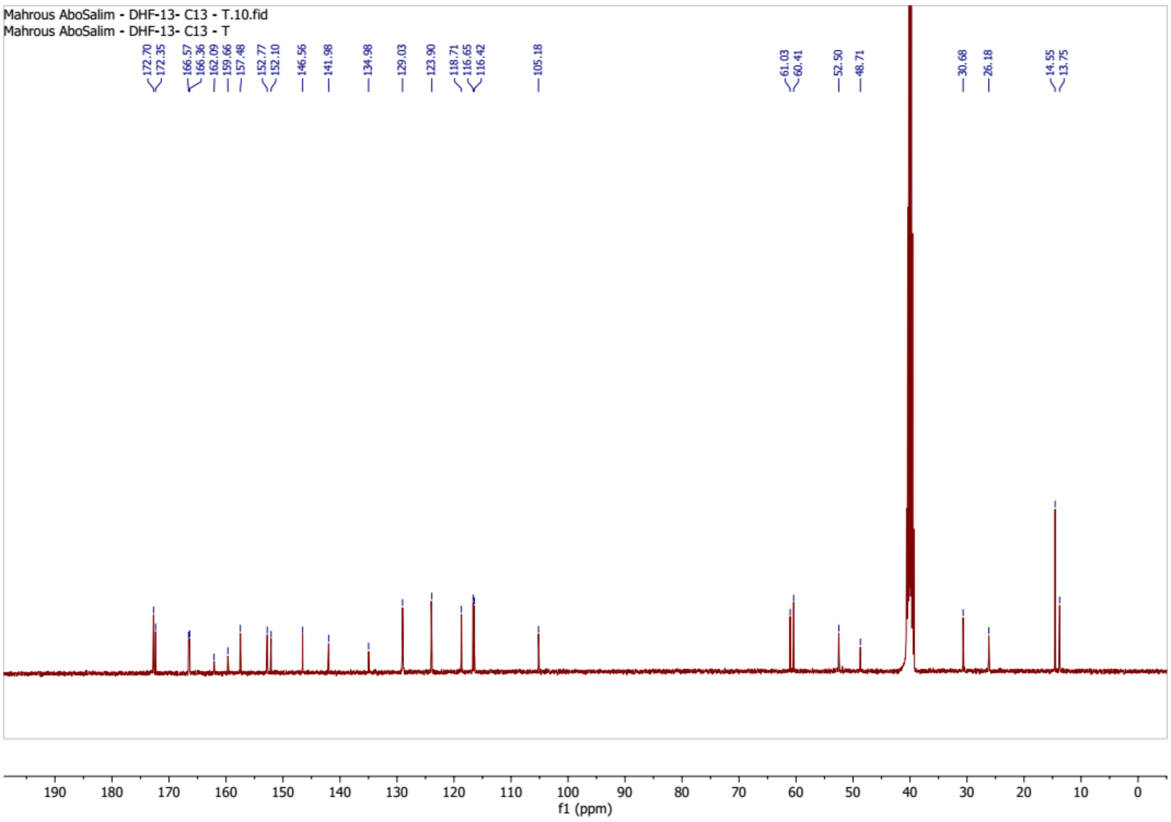

Figure S 12. ^{13}C NMR of **6e.**

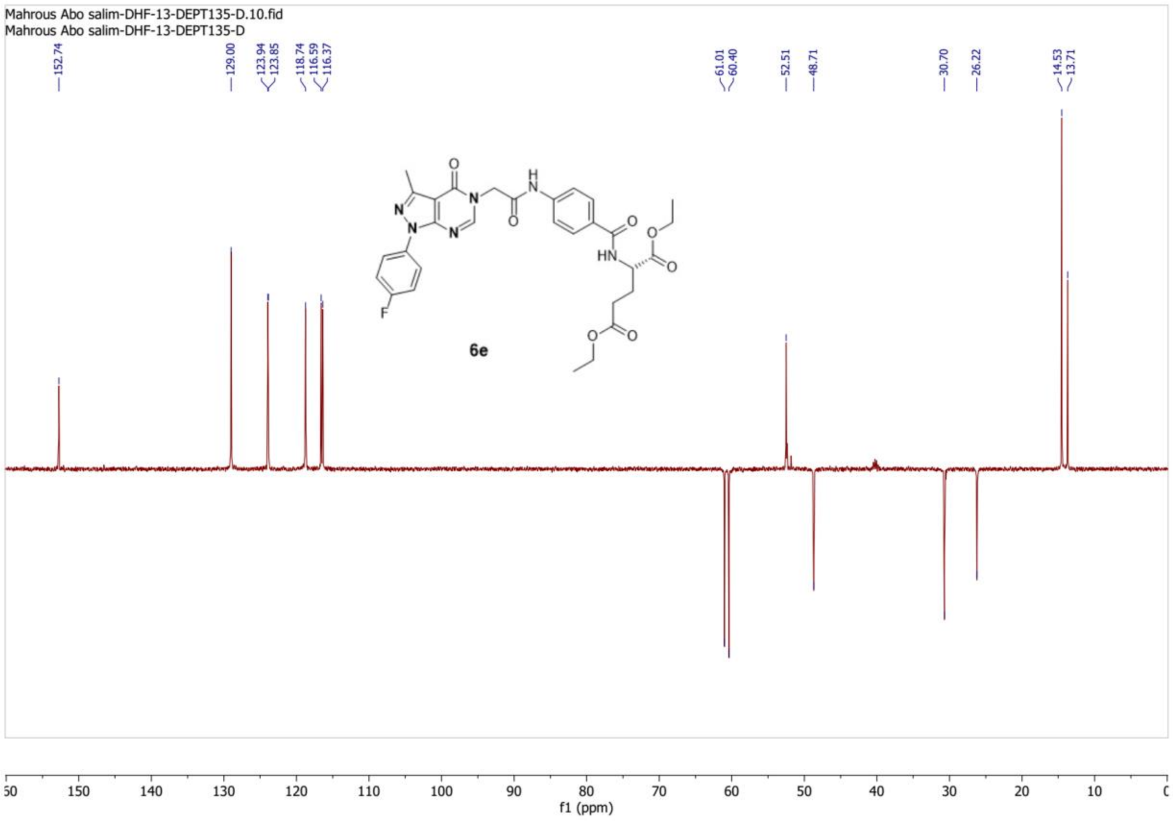

Figure S 13. DEPT135 ^{13}C NMR of **6e**.

1.7 6f

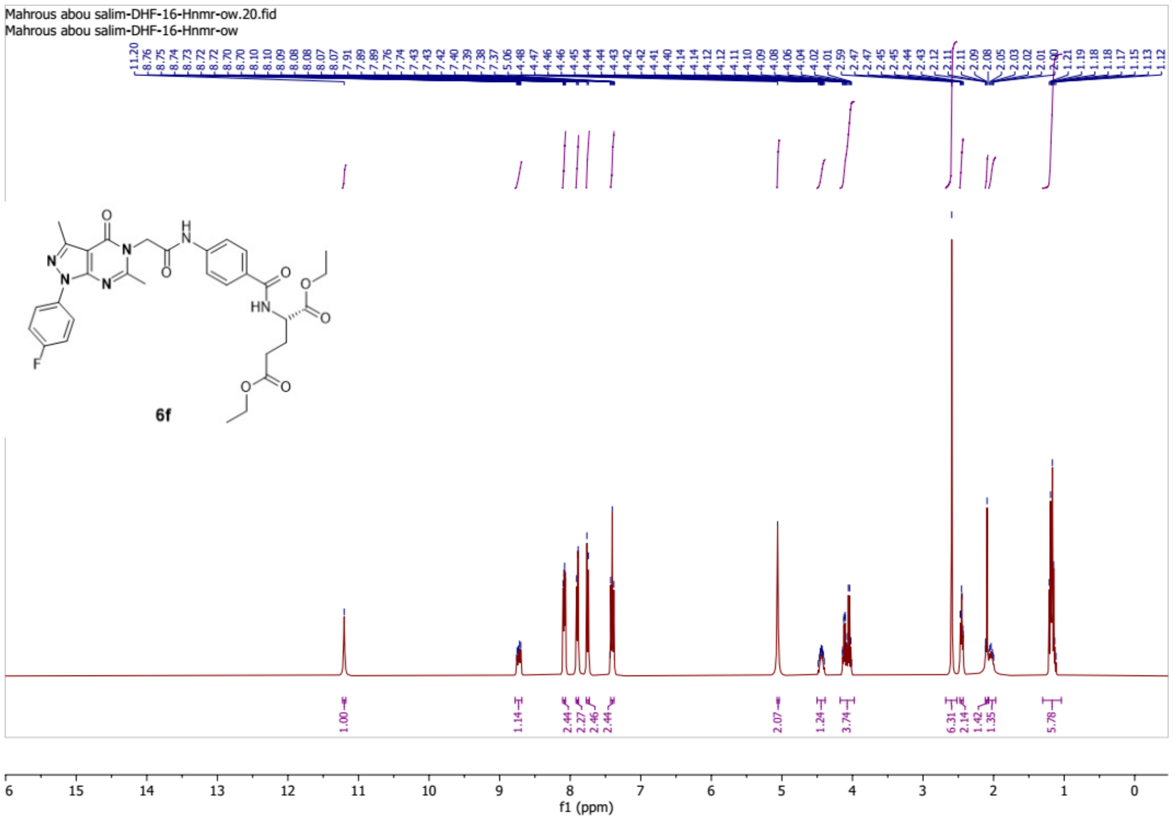

Figure S 14. ^{1}H NMR of **6f.**

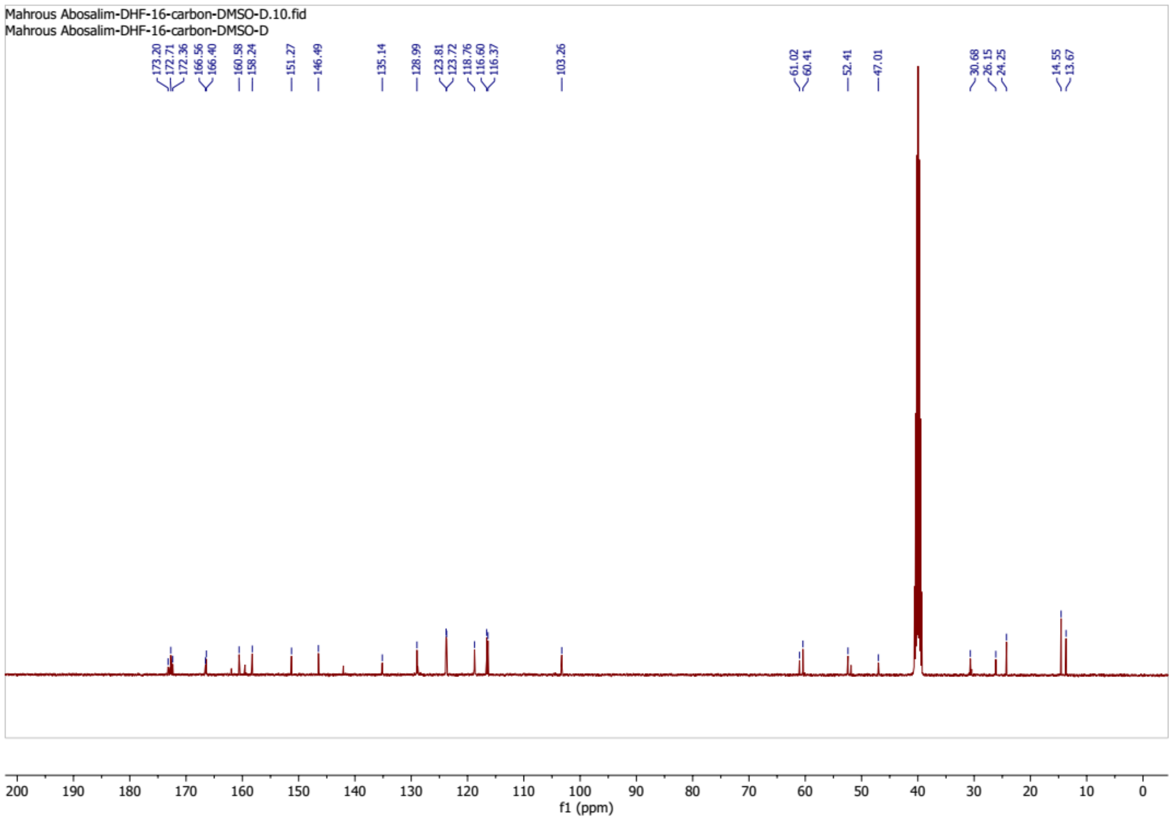

Figure S 15. ^{13}C NMR of **6f.**

1.8 6g

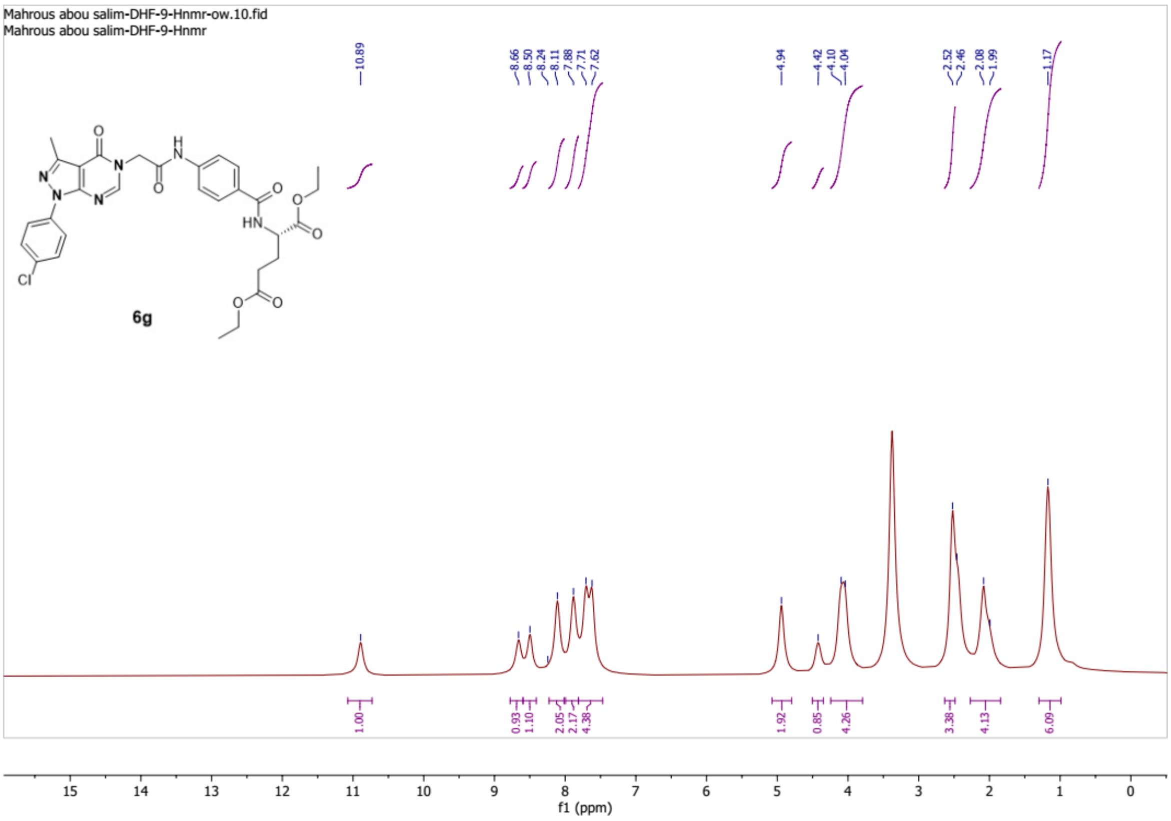

Figure S 16. ^{1}H NMR of **6g.**

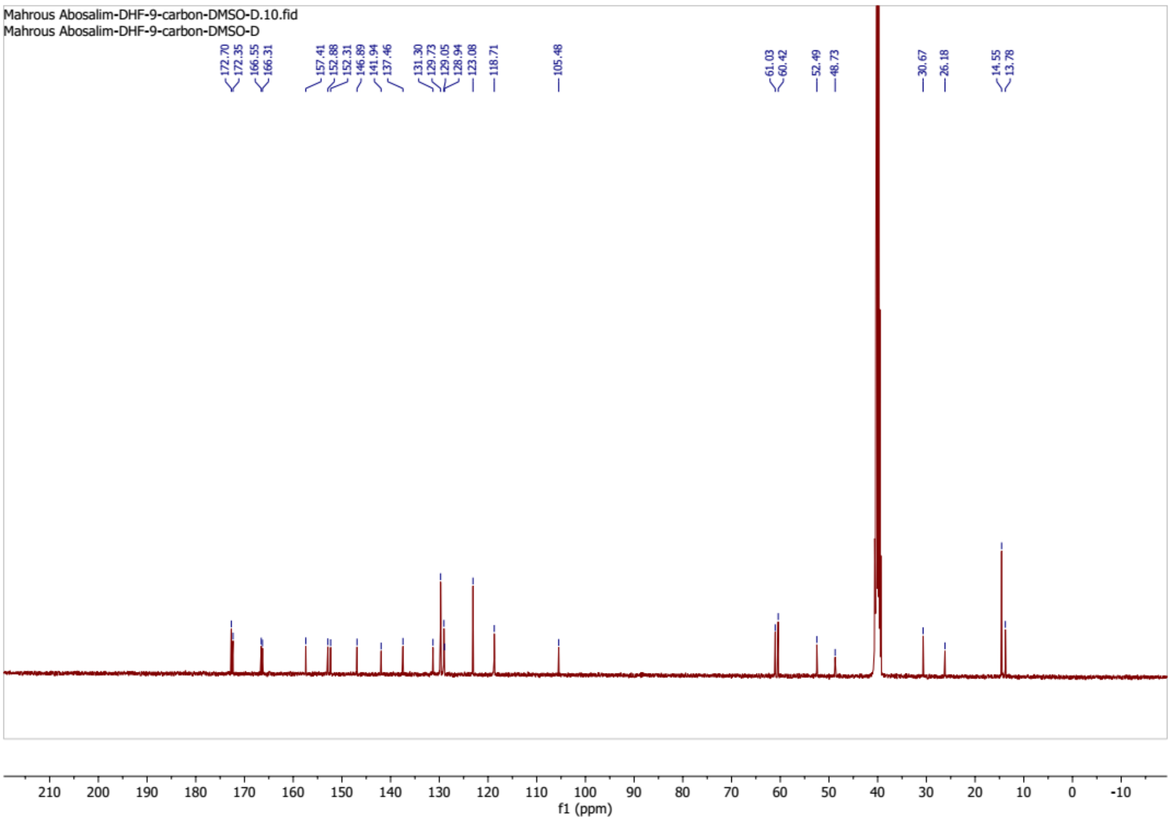

Figure S 17. ^{13}C NMR of **6g.**

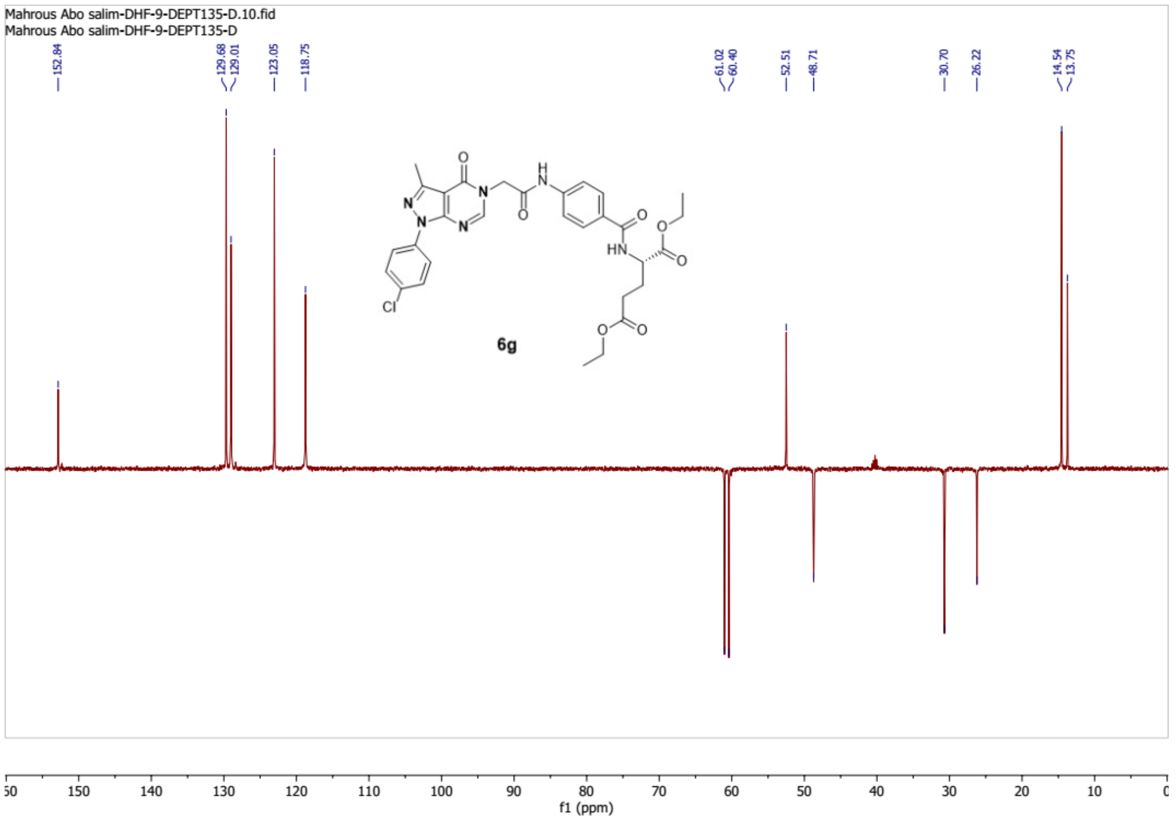

Figure S 18. DEPT135 ^{13}C NMR of **6g**.

1.9 6h

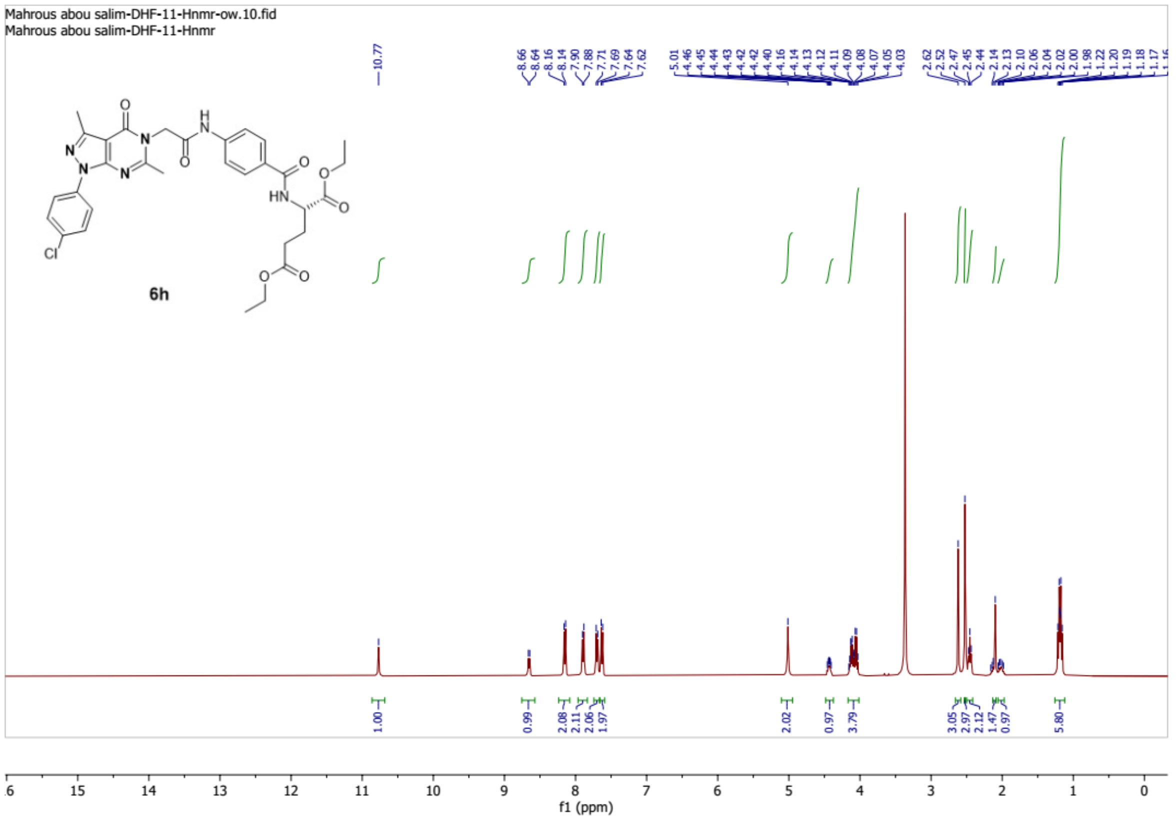

Figure S 19. ^{1}H NMR of **6h.**

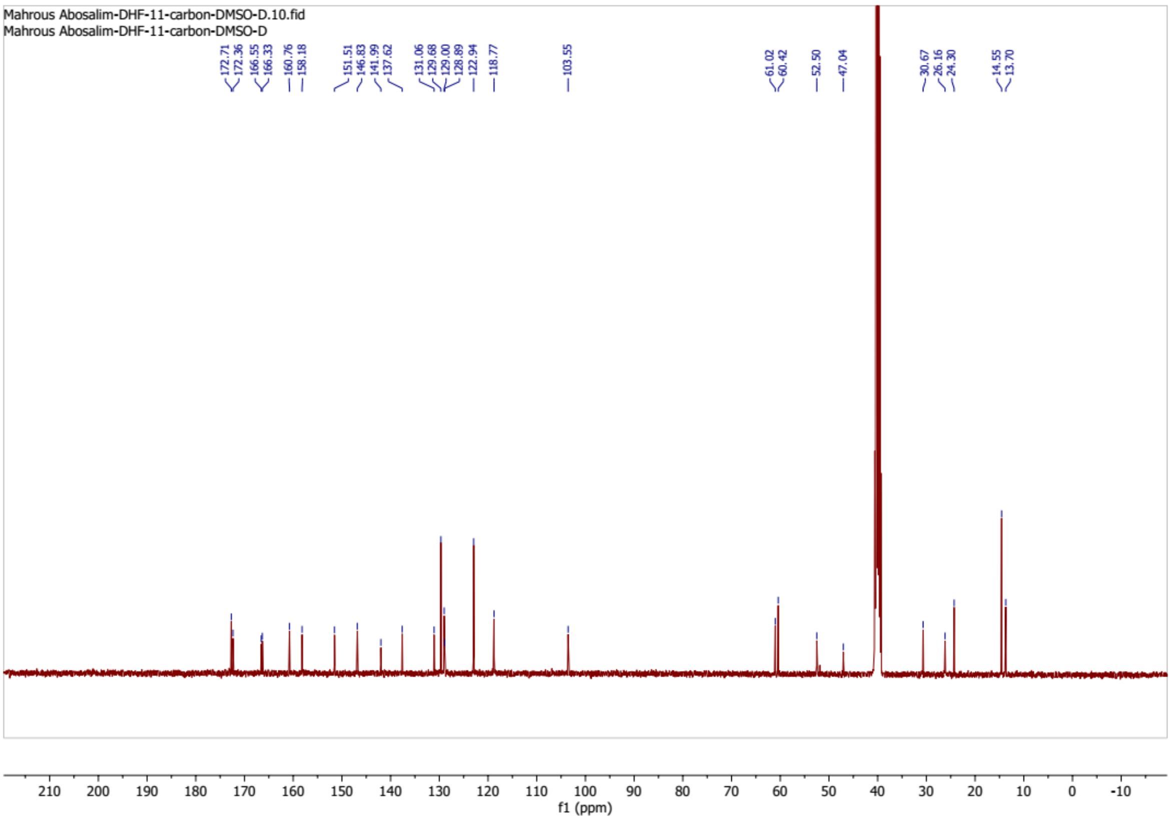

Figure S 20. ^{13}C NMR of **6h.**

1.10 6i

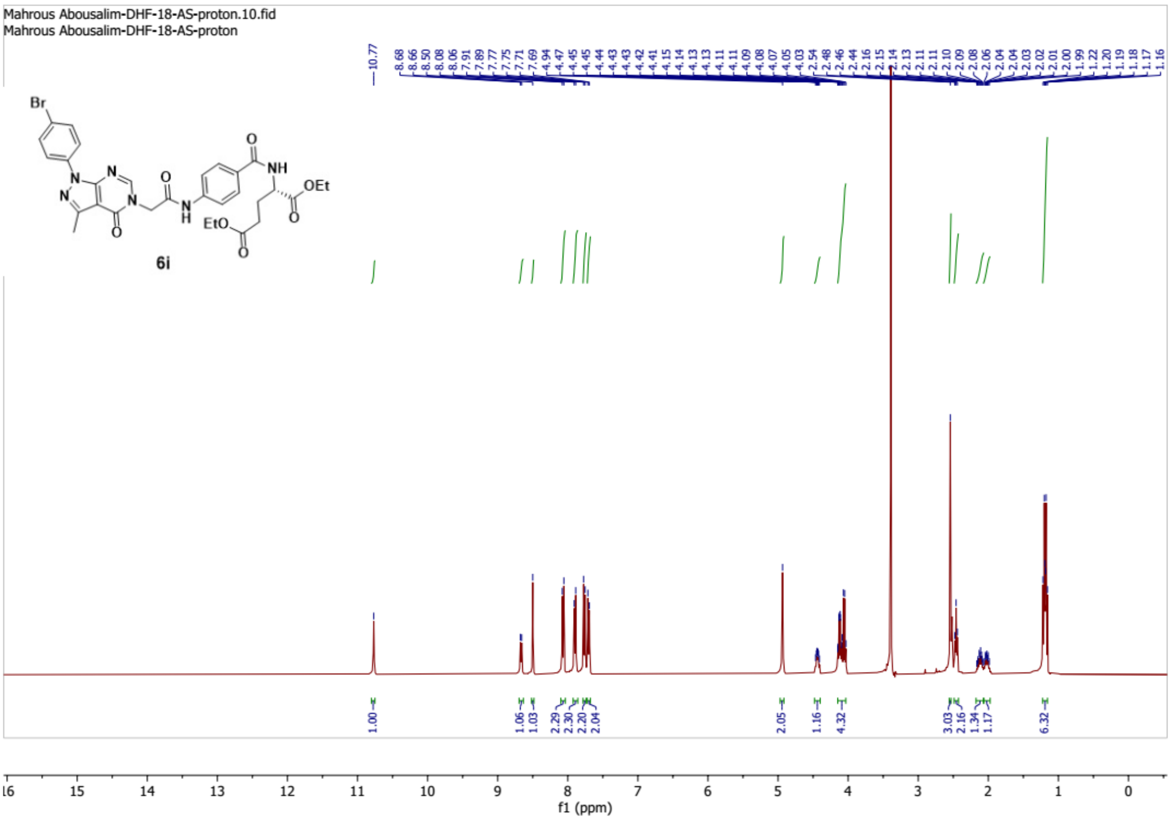

Figure S 21. ^{1}H NMR of **6i.**

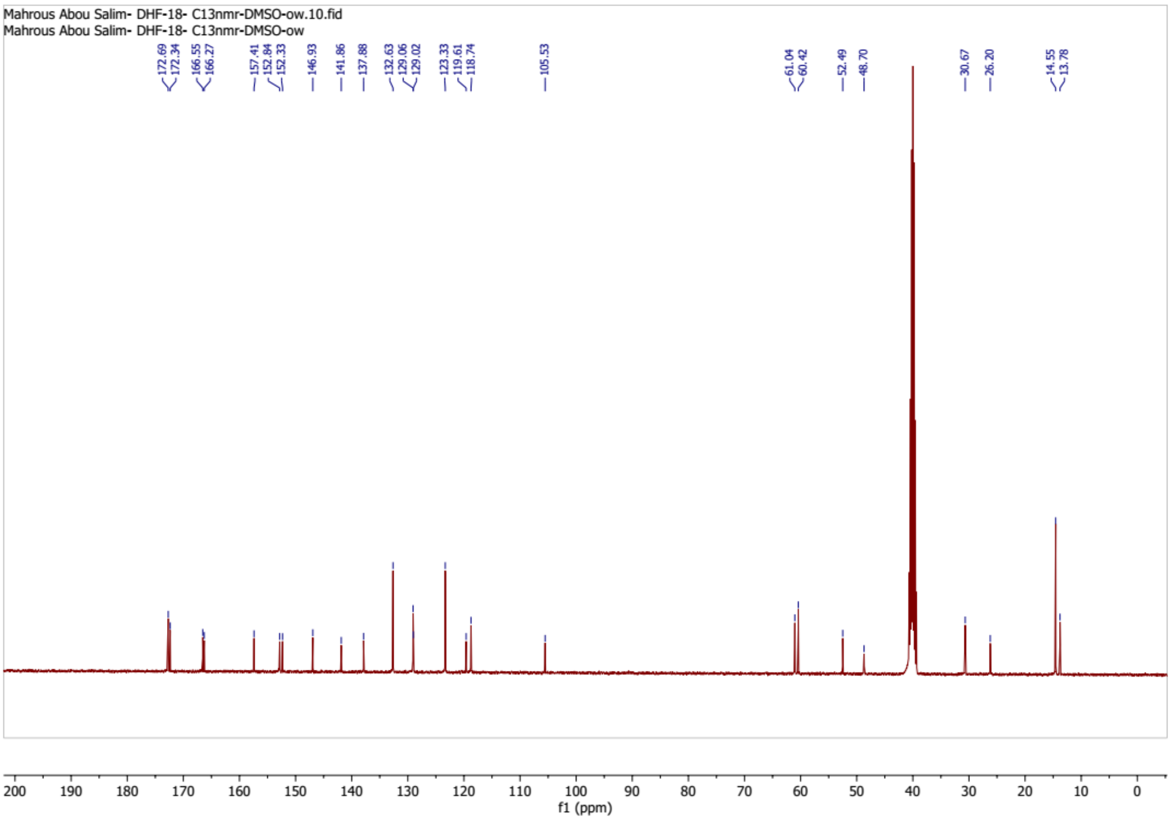

Figure S 22. ^{13}C NMR of **6i.**

1.11 6j

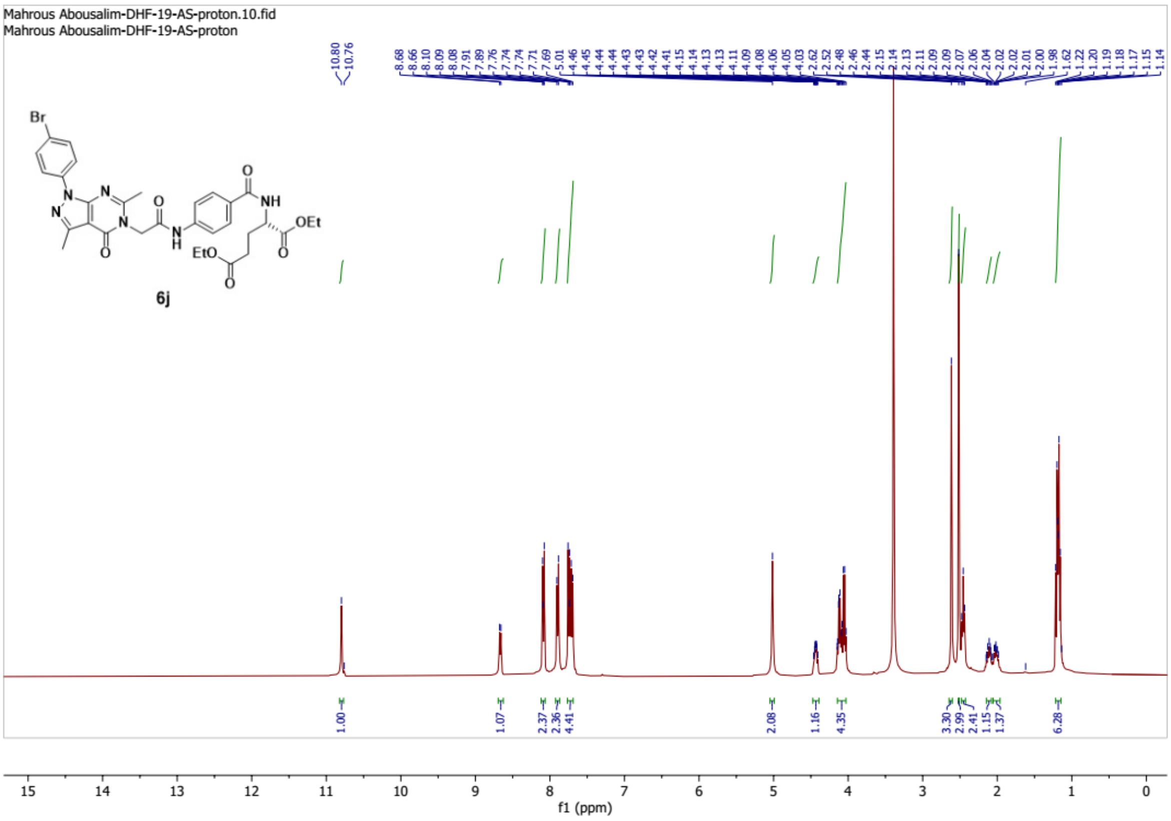

Figure S 23. ^{1}H NMR of **6j.**

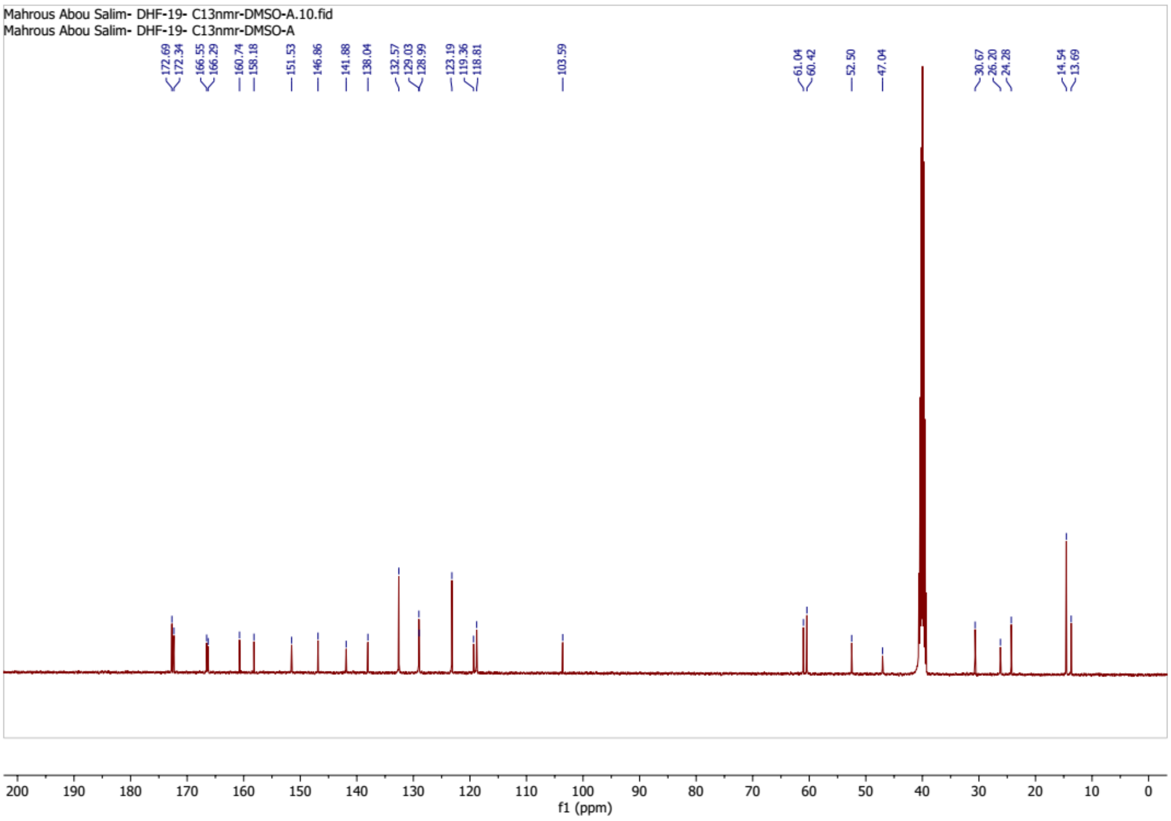

Figure S 24. ^{13}C NMR of **6j.**

1.12 6k

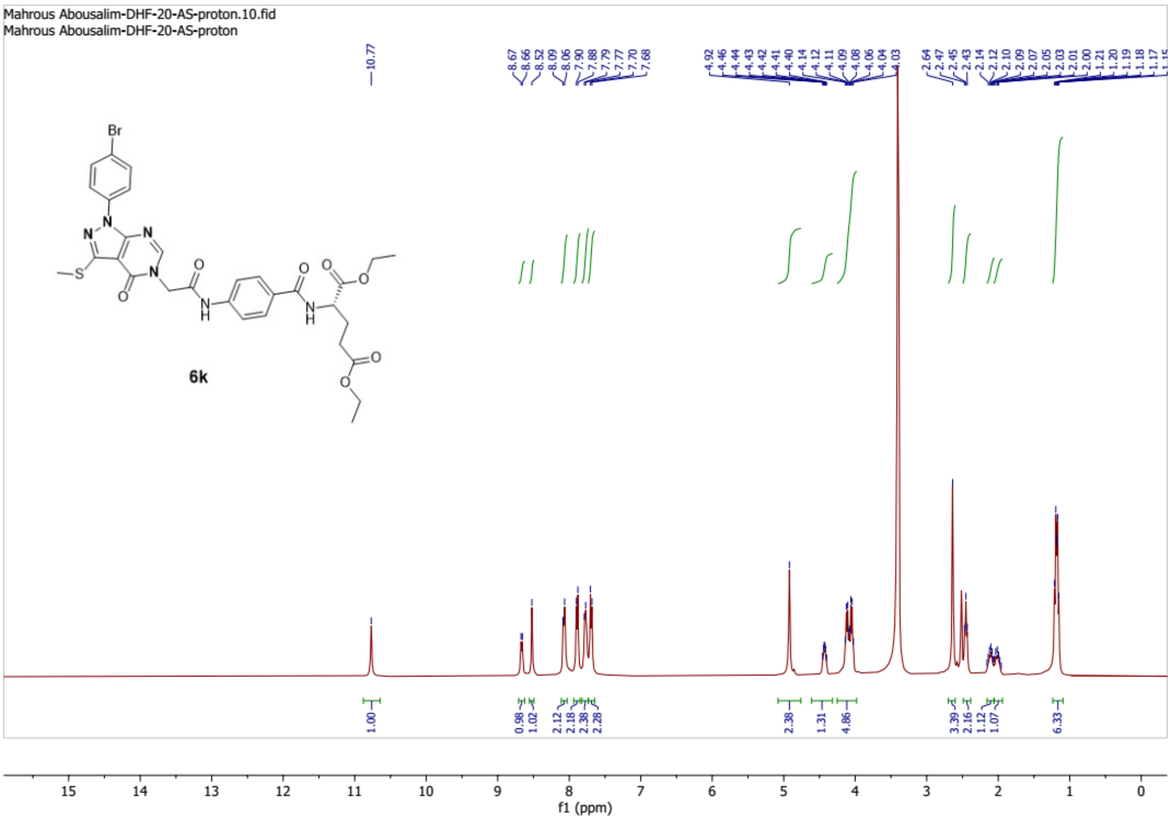

Figure S 25. ^{1}H NMR of **6k.**

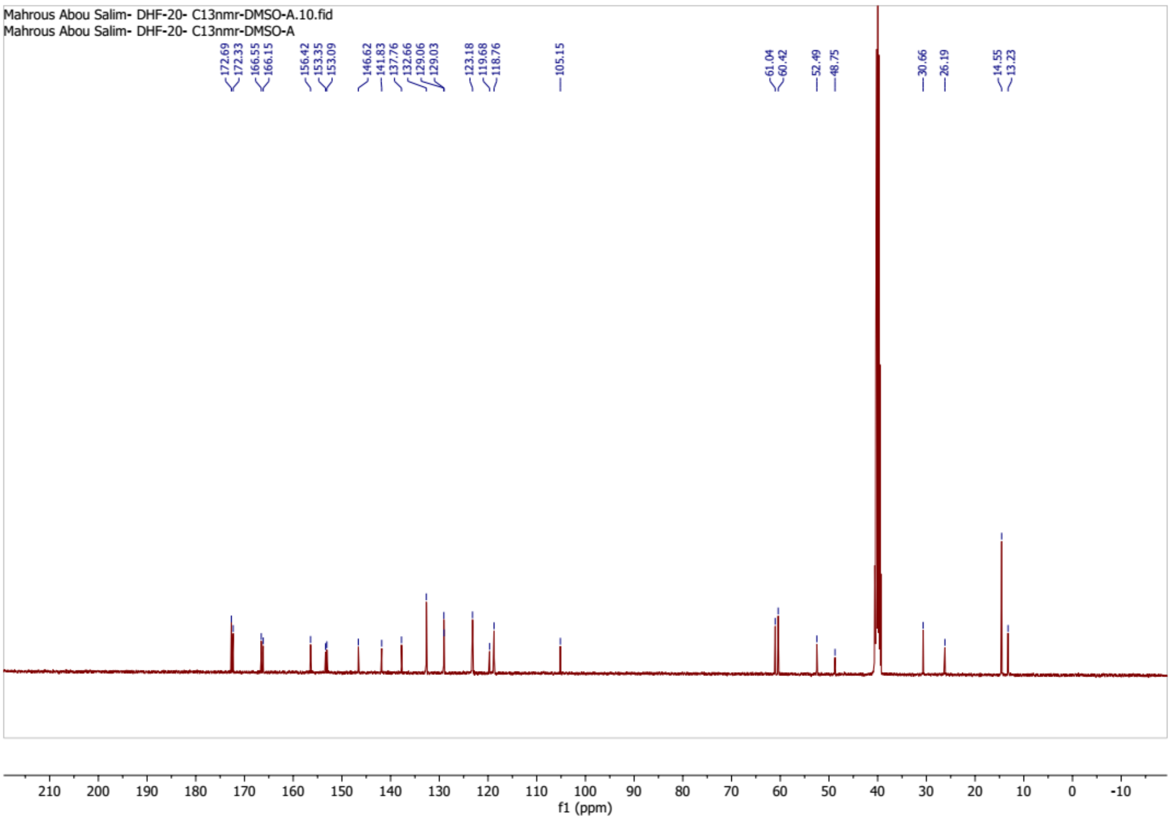

Figure S 26. ^{13}C NMR of **6k.**

1.13 6l

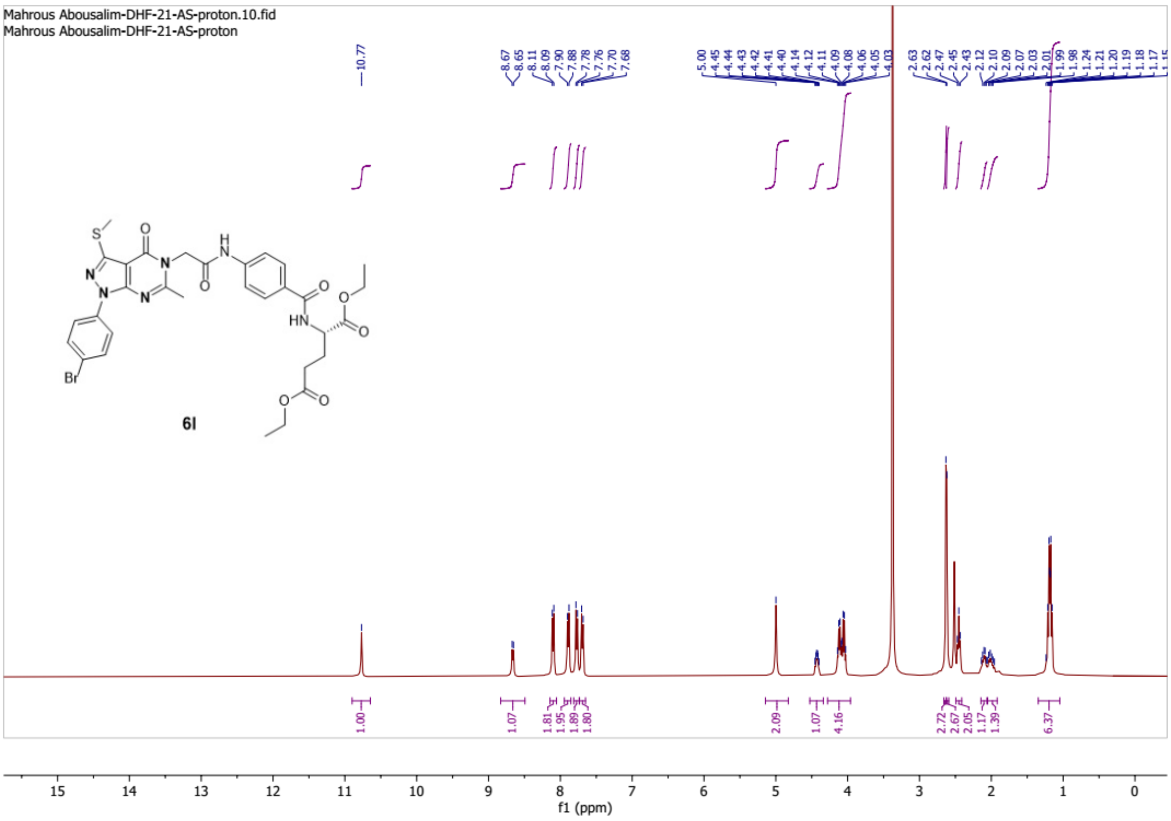

Figure S 27. ^{1}H NMR of **6l.**

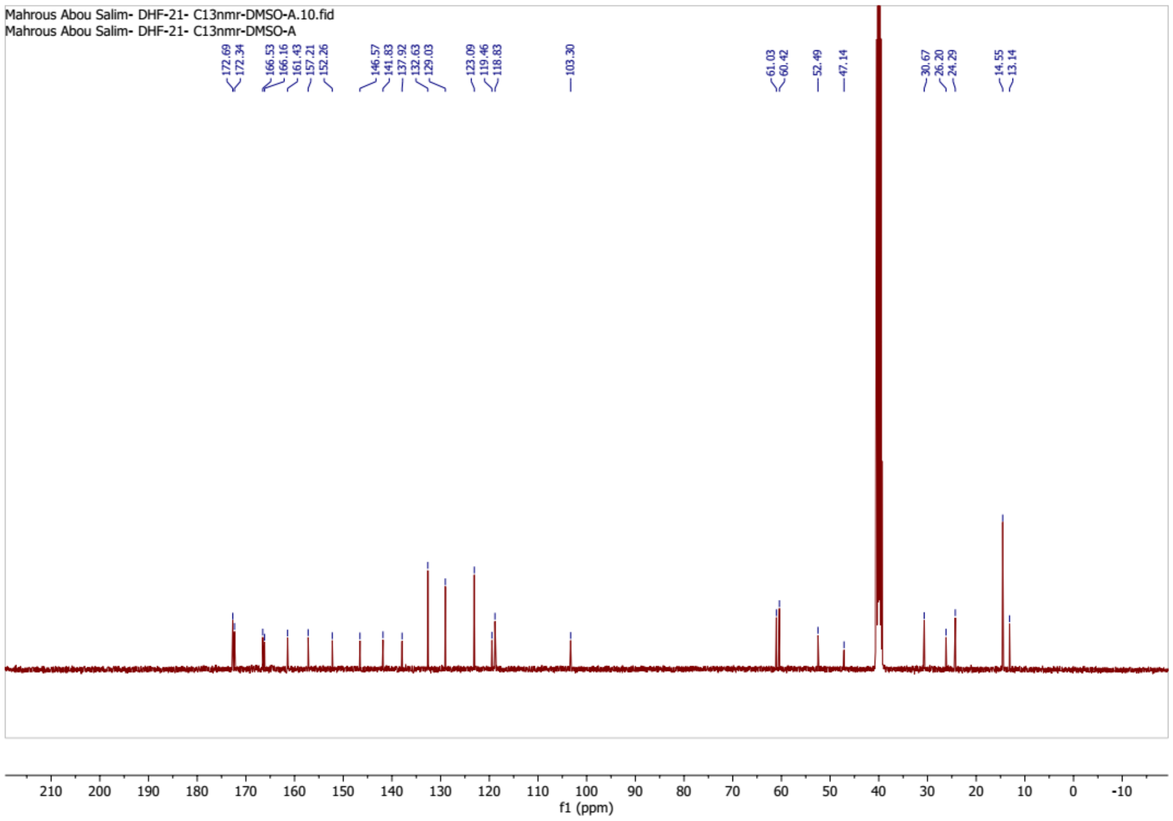

Figure S 28. ^{13}C NMR of **6l.**

1.14 7a

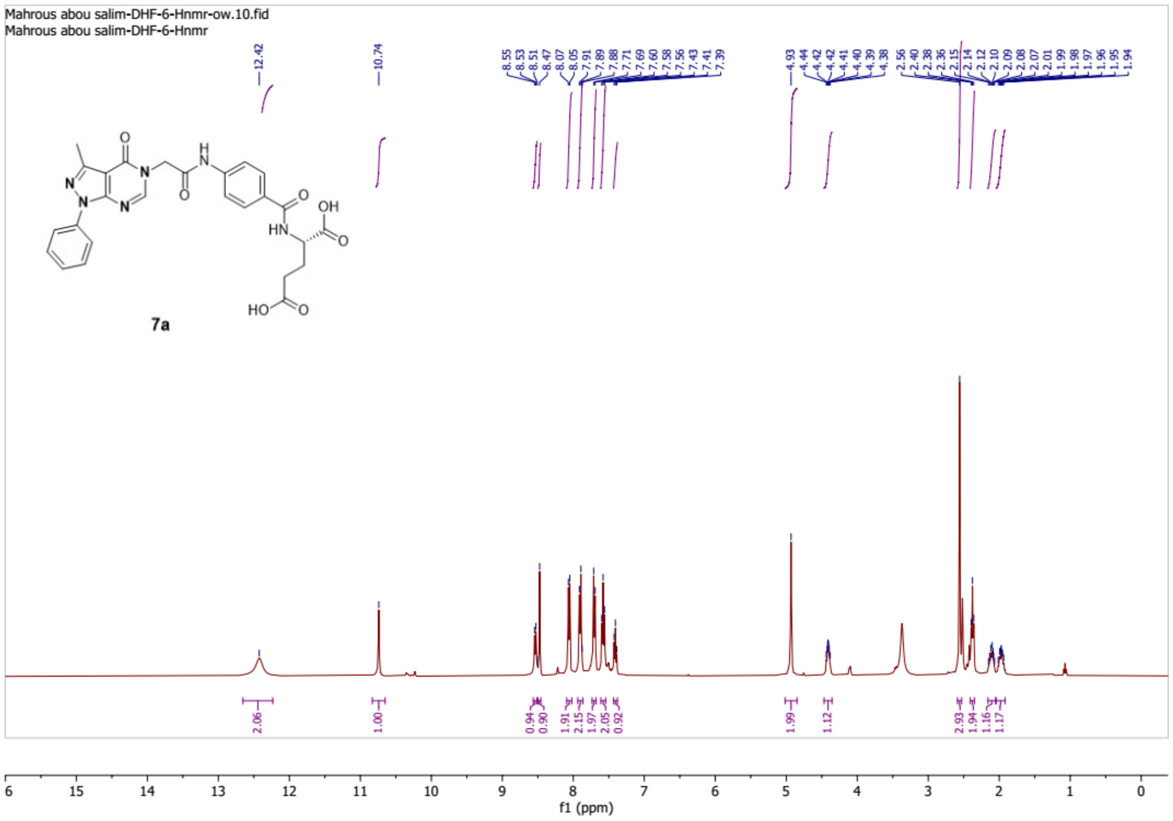

Figure S 29. ^{13}C NMR of **7a.**

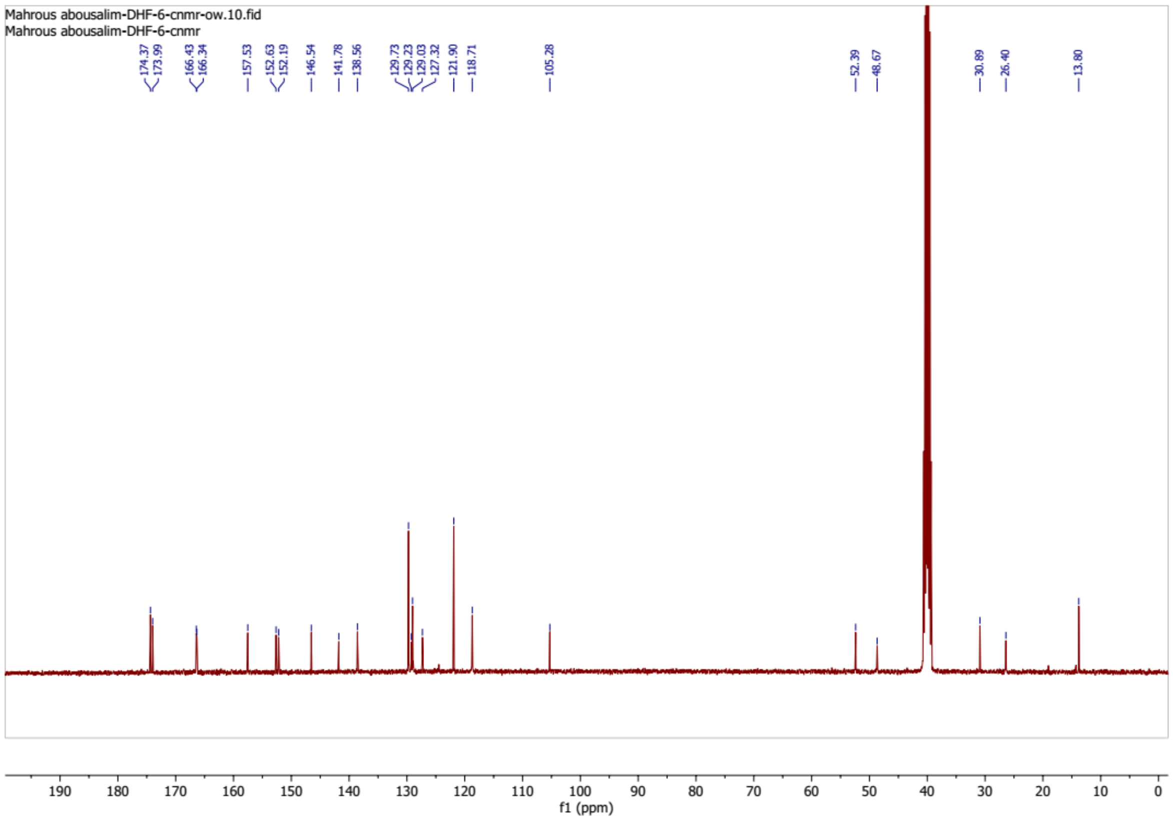

Figure S 30. ^{13}C NMR of **7a.**

1.15 7b

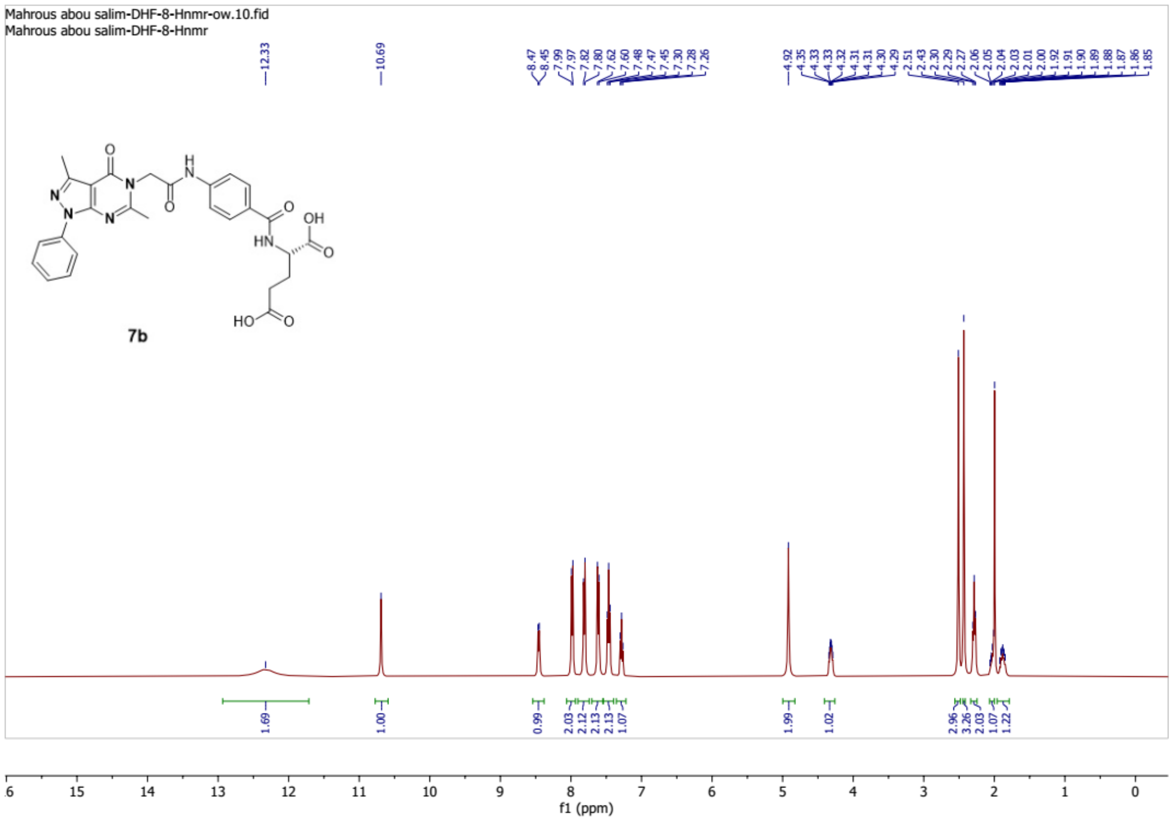

Figure S 31. ^{1}H NMR of **7b.**

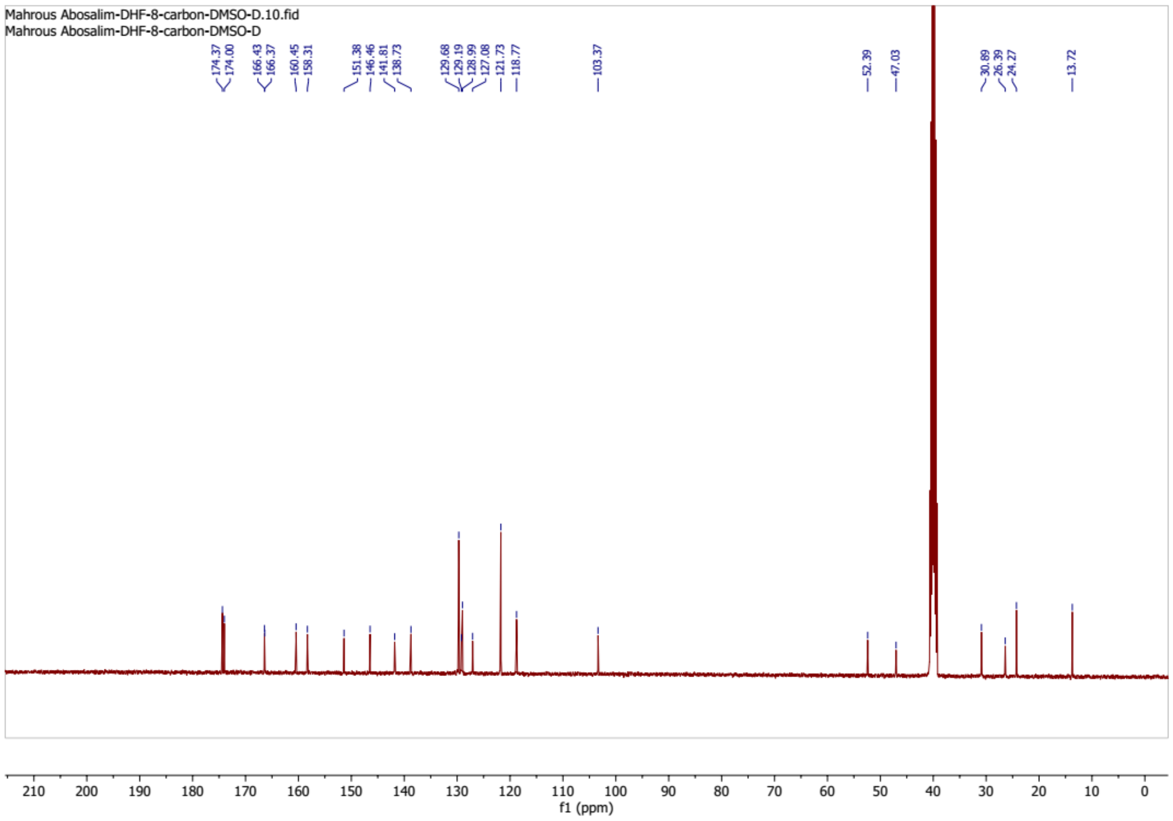

Figure S 32. ^{13}C NMR of **7b.**

1.16 9a

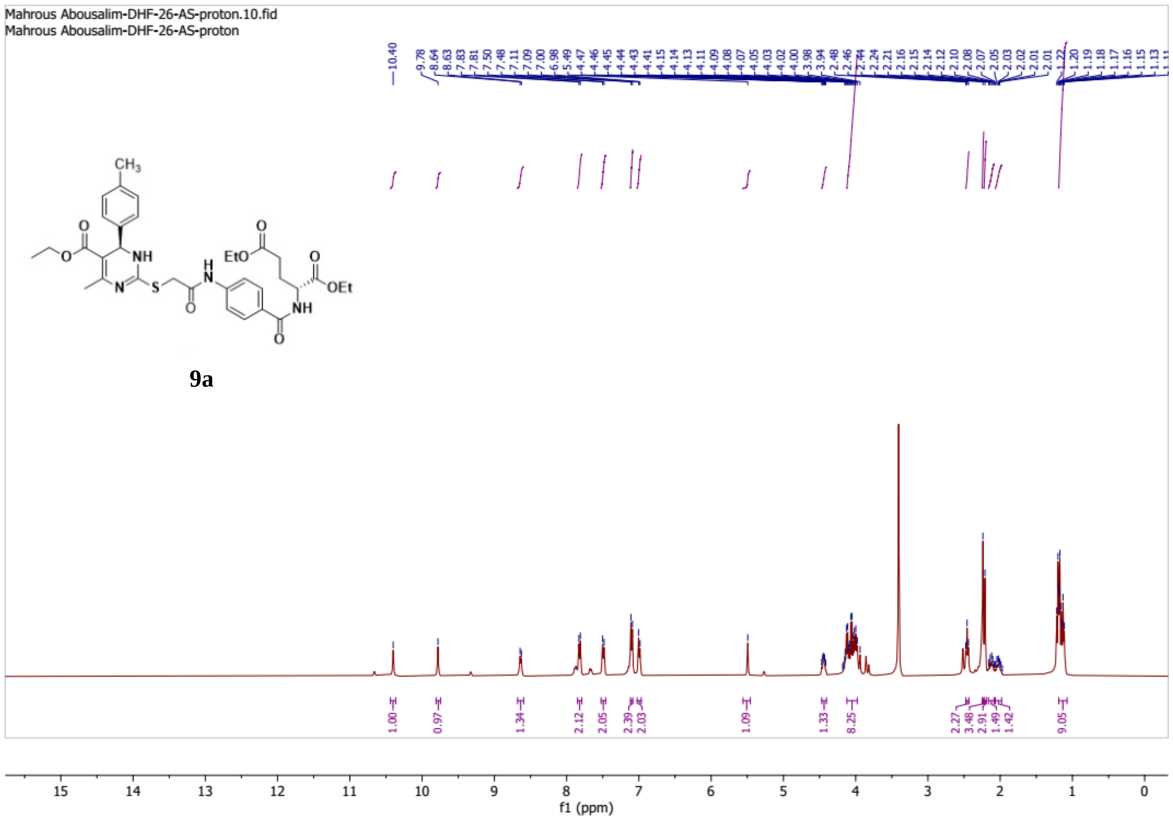

Figure S 33. ^{1}H NMR of **9a.**

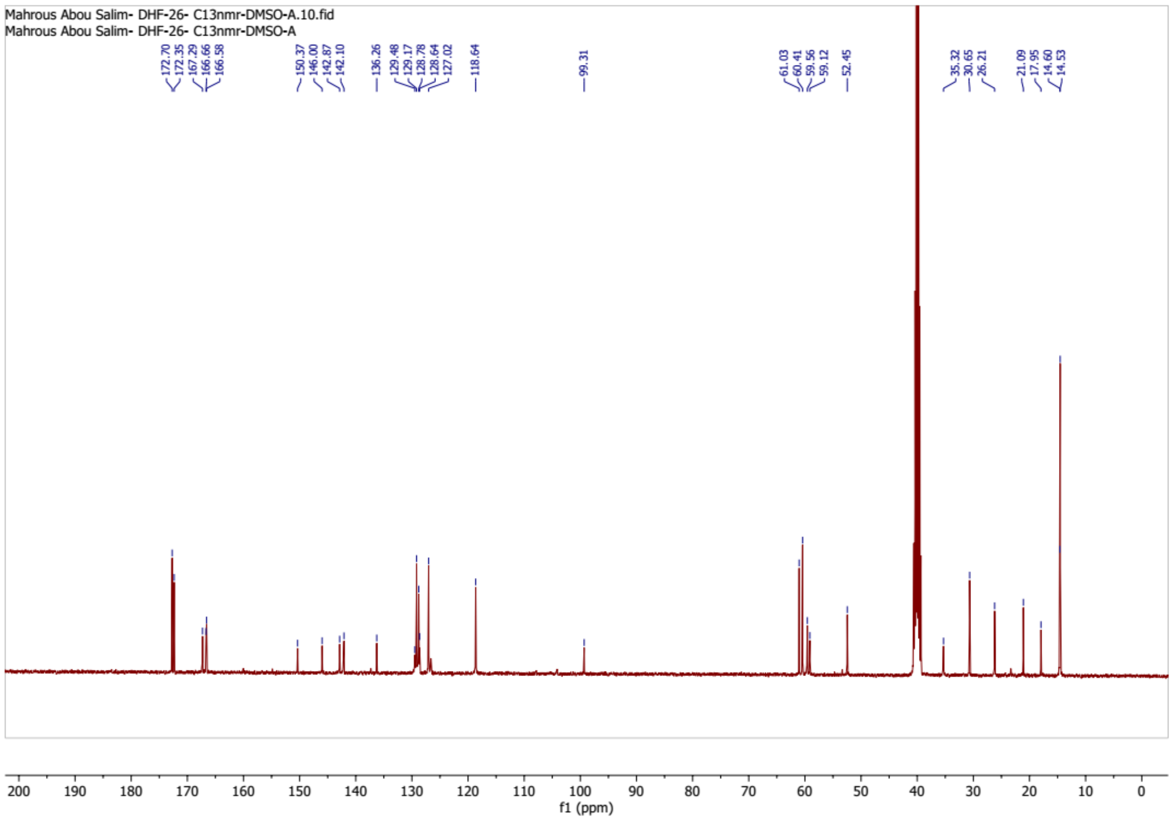

Figure S 34. ^{13}C NMR of **9a.**

1.17 9b

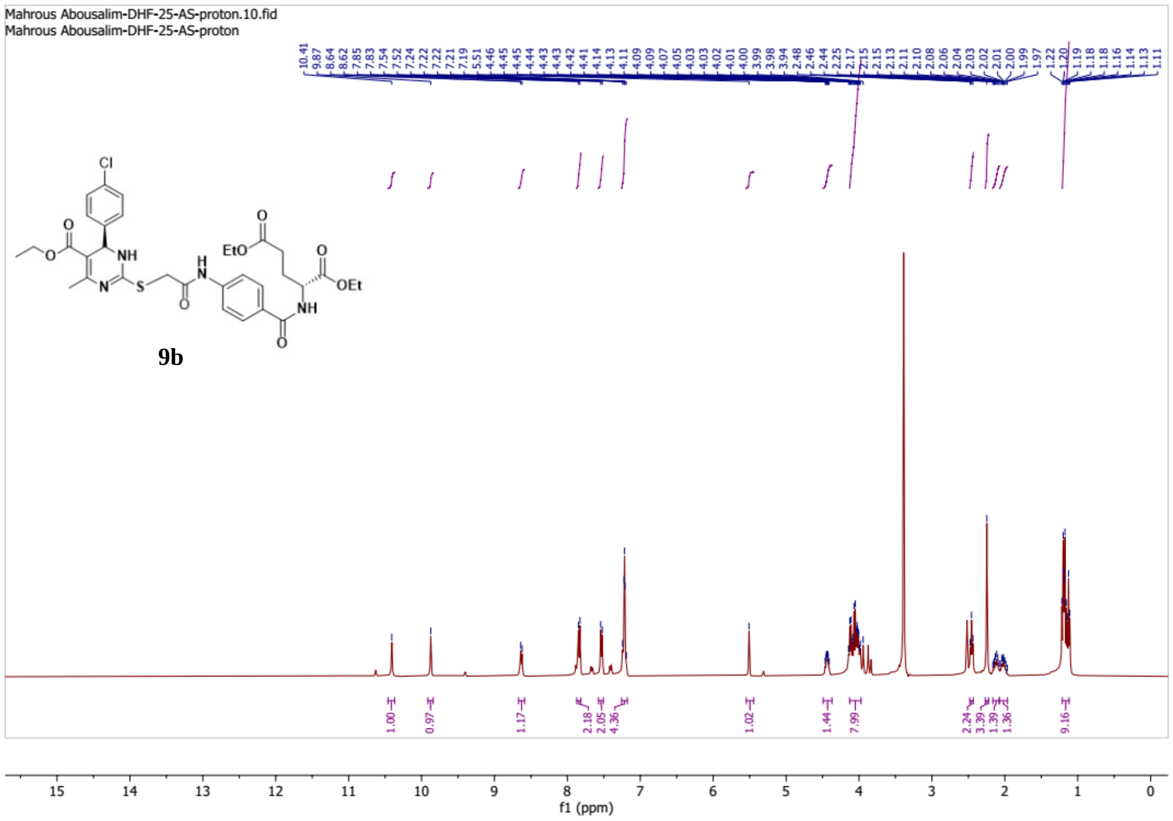

Figure S 35. ^{1}H NMR of **9b.**

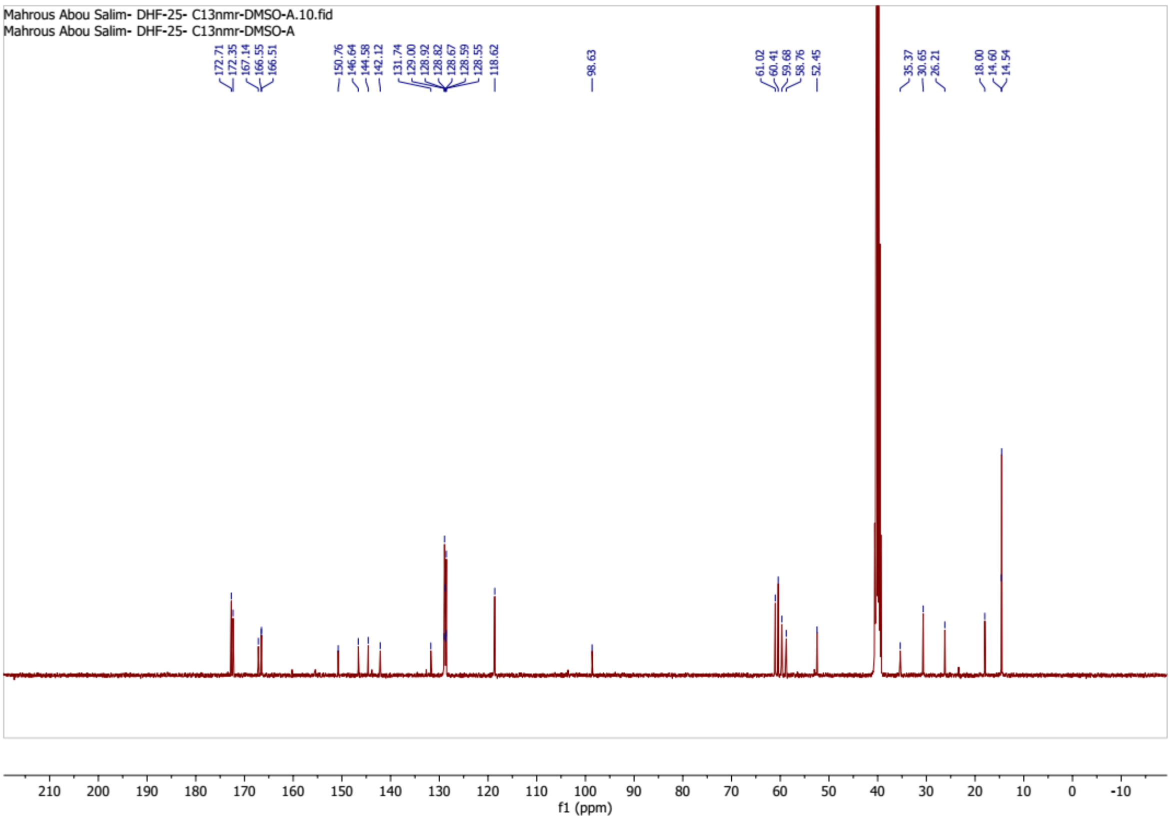

Figure S 36. ^{13}C NMR of **9b.**

1.18 9c

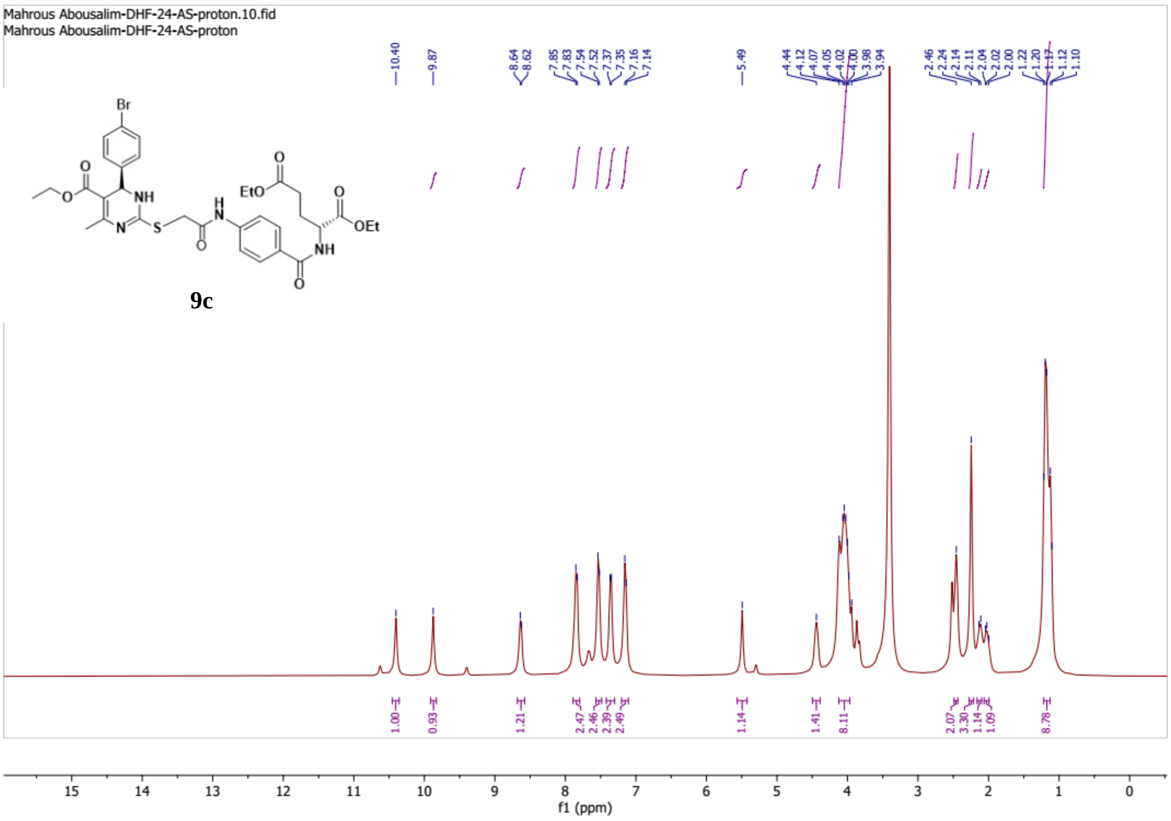

Figure S 37. ^{1}H NMR of **9c.**

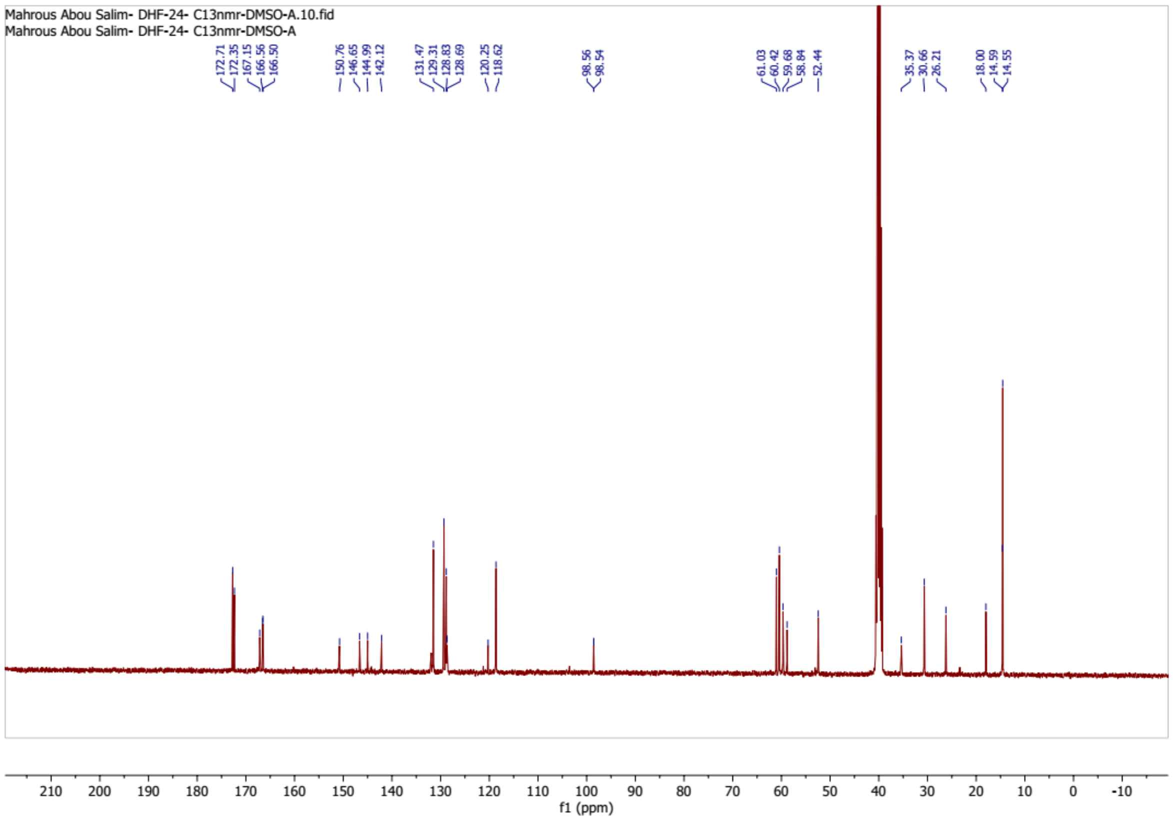

Figure S 38. ^{13}C NMR of **9c.**

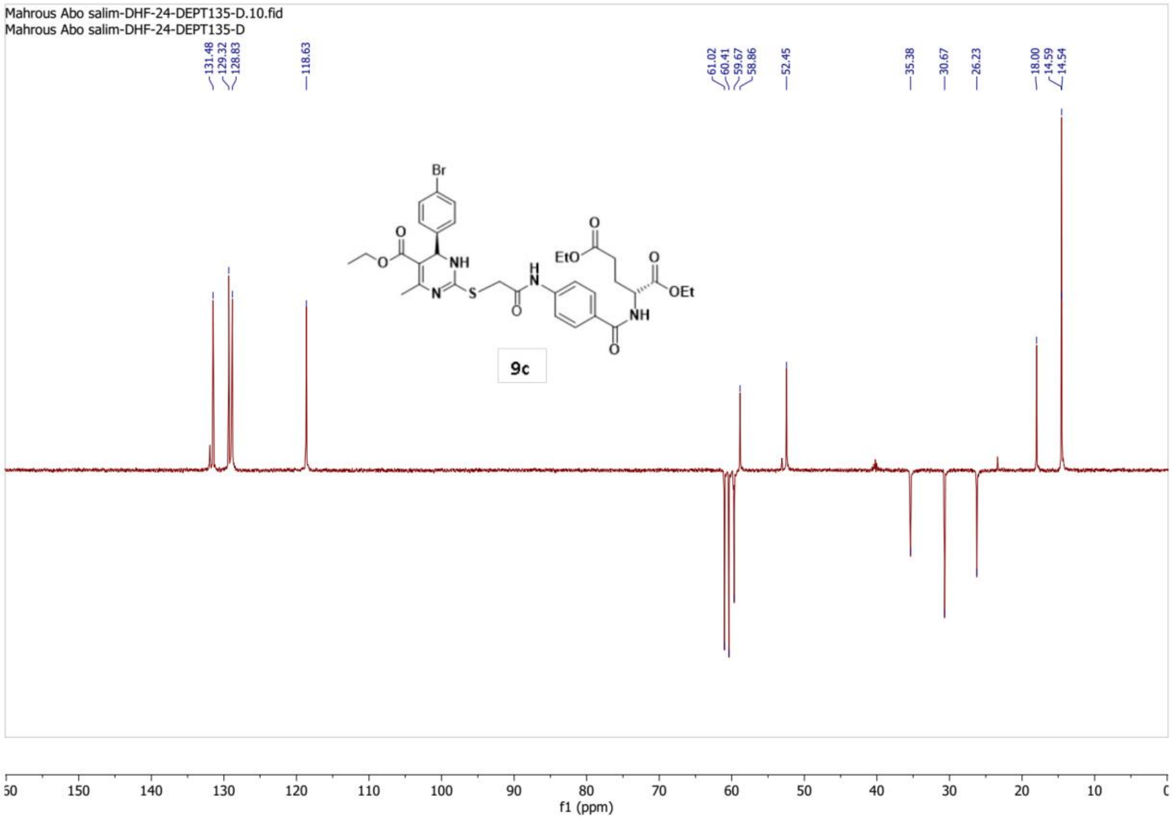

Figure S 39. DEPT135 ^{13}C NMR of **9c**.

2 3D representation of MTX and PMX inside the active sites

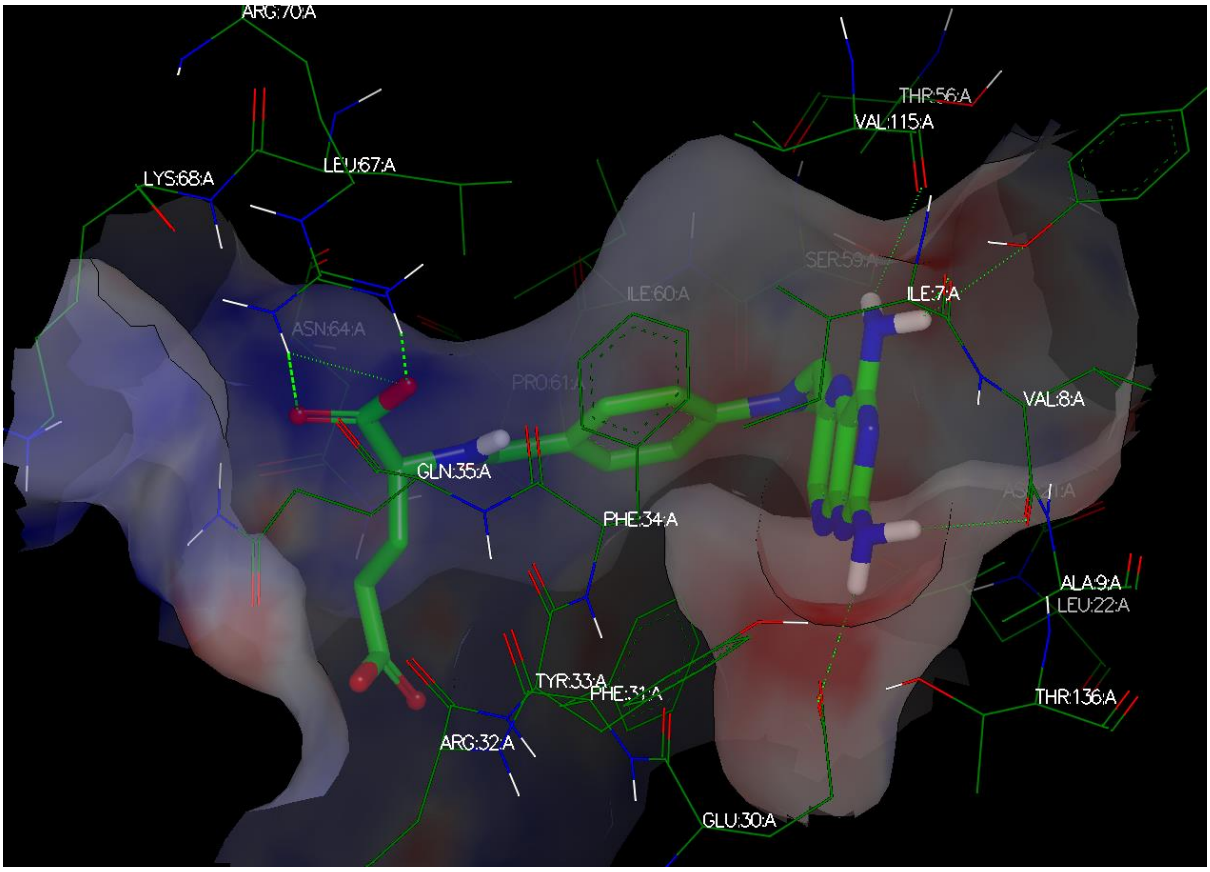

Figure S 40. 3D representation of MTX in the active site of DHFR (PDB ID: 1U72)

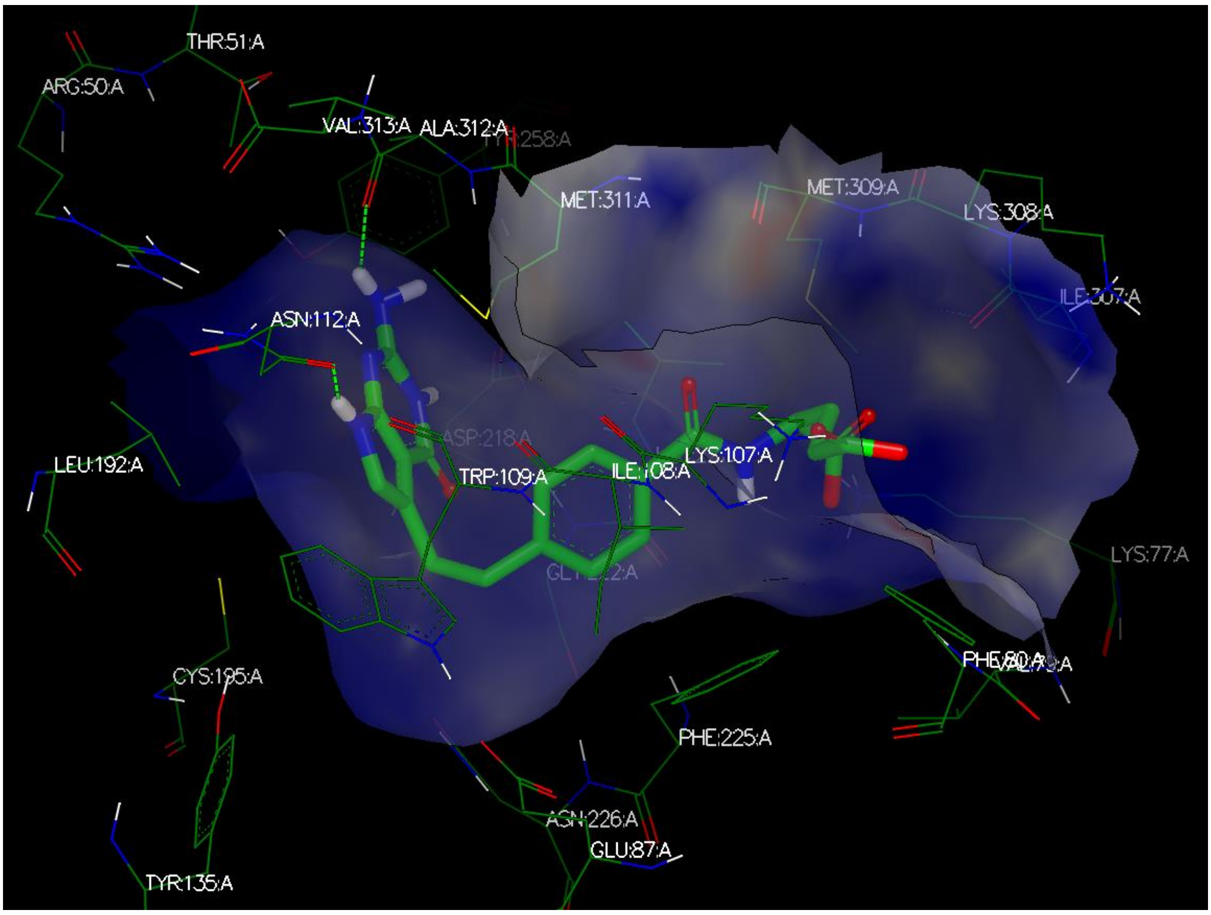

Figure S 41. 3D representation of PMX in the active site of TS (PDB ID: 1JU6).

3 NCI-60 Cell line screening; One-Dose Screen

3.1 6a

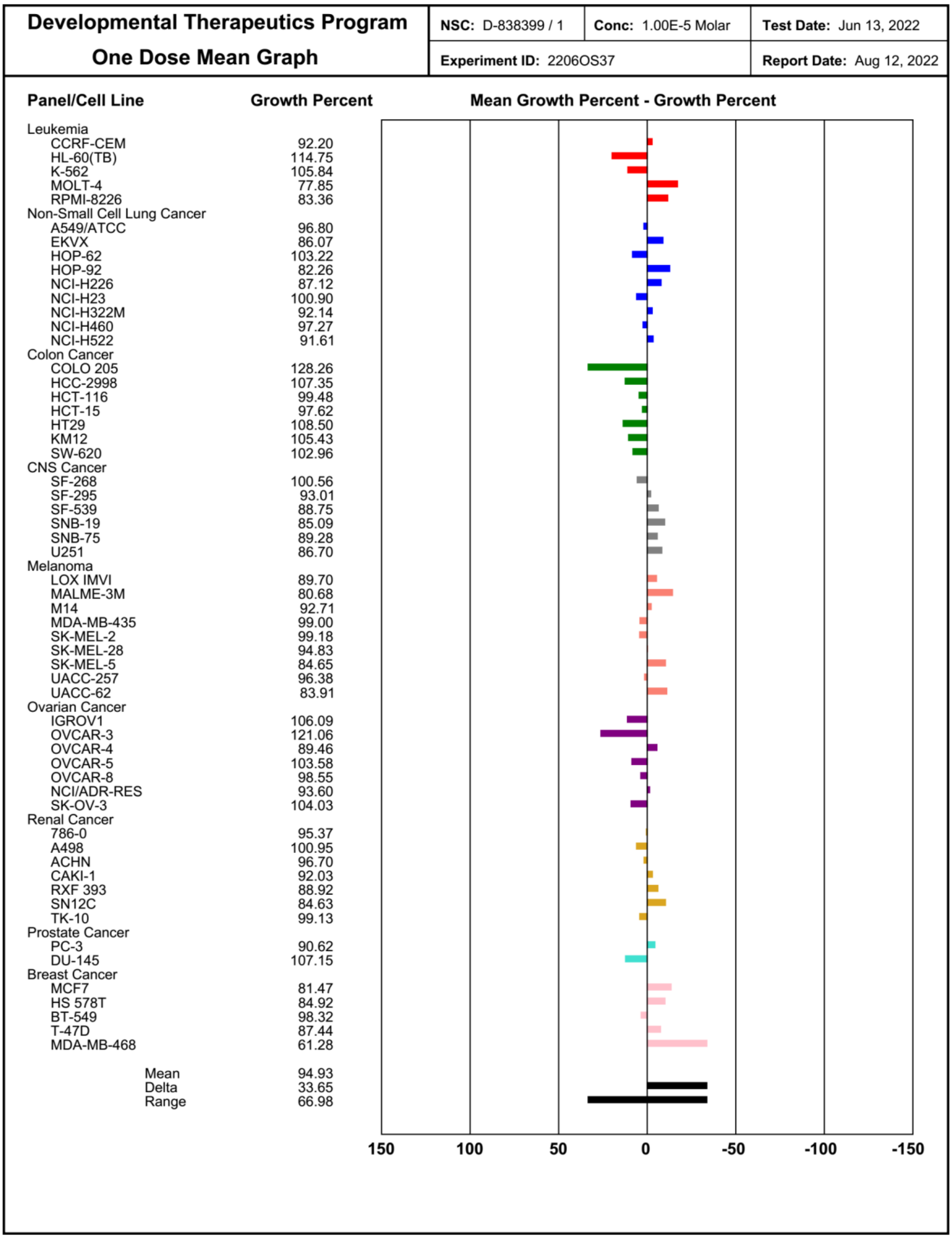

Figure S 42. One dose mean graph of **6a**.

3.2 6b

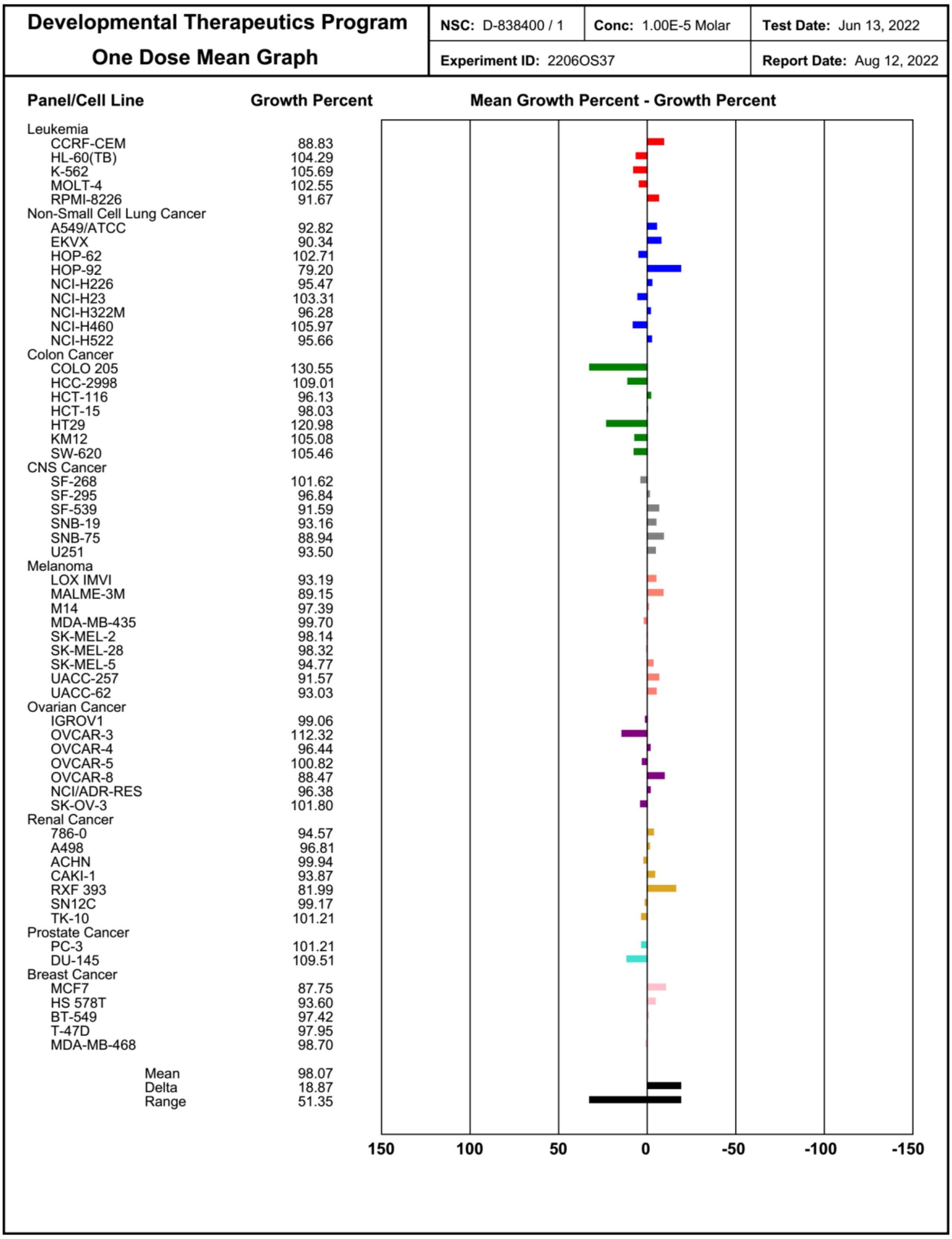

Figure S 43. One dose mean graph of **6b.**

3.3 6c

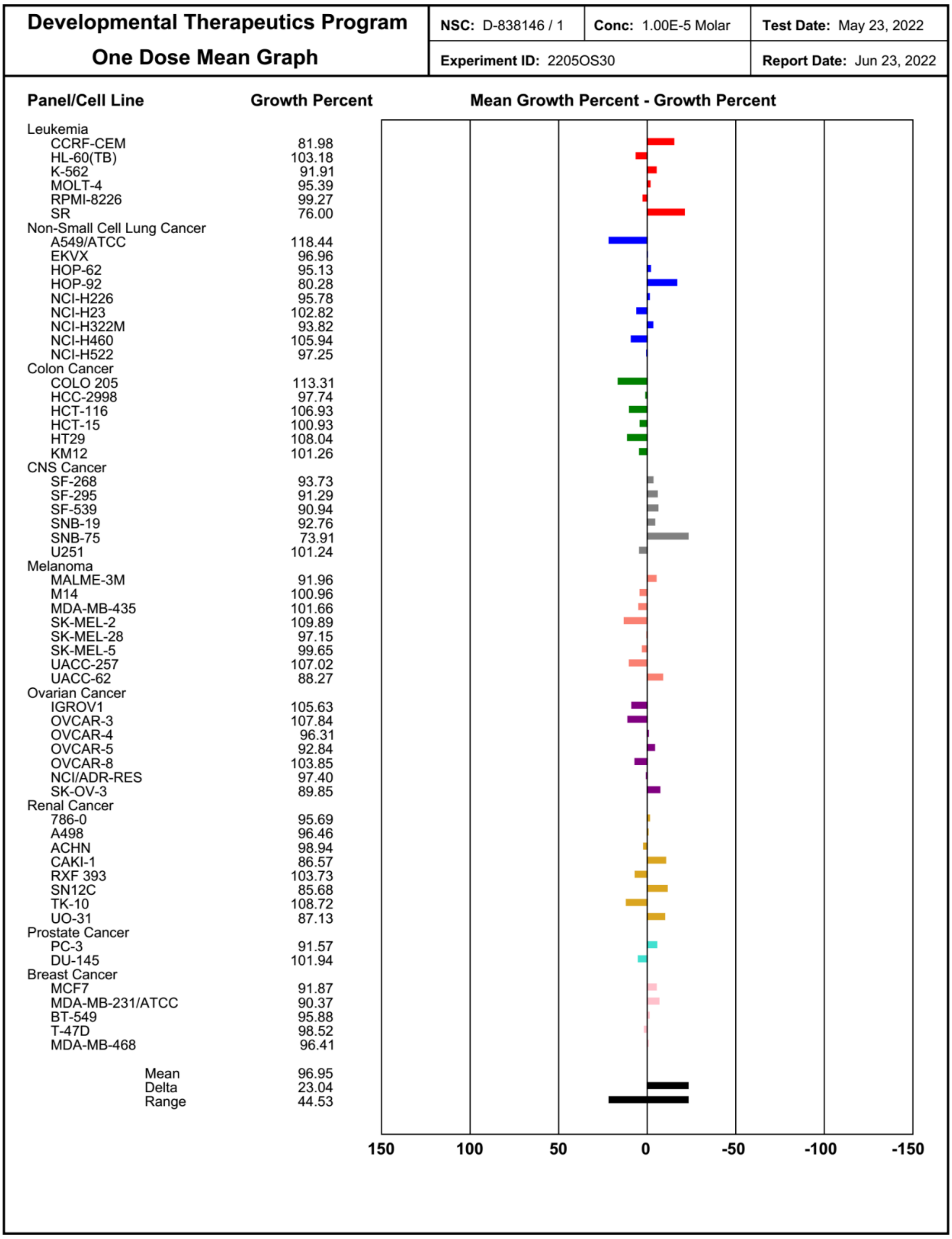

Figure S 44. One dose mean graph of **6c**.

3.4 6d

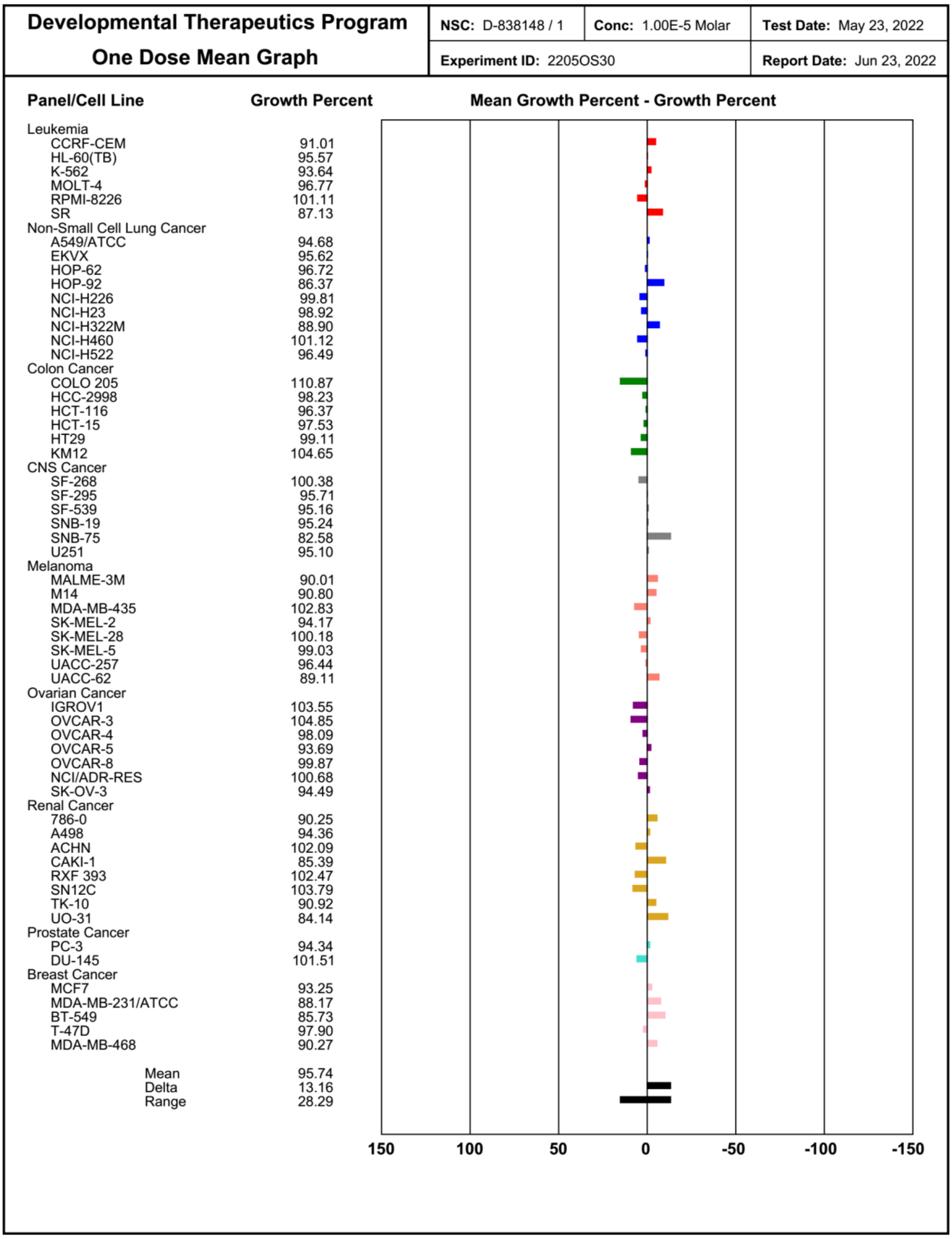

Figure S 45. One dose mean graph of **6d**.

3.5 6e

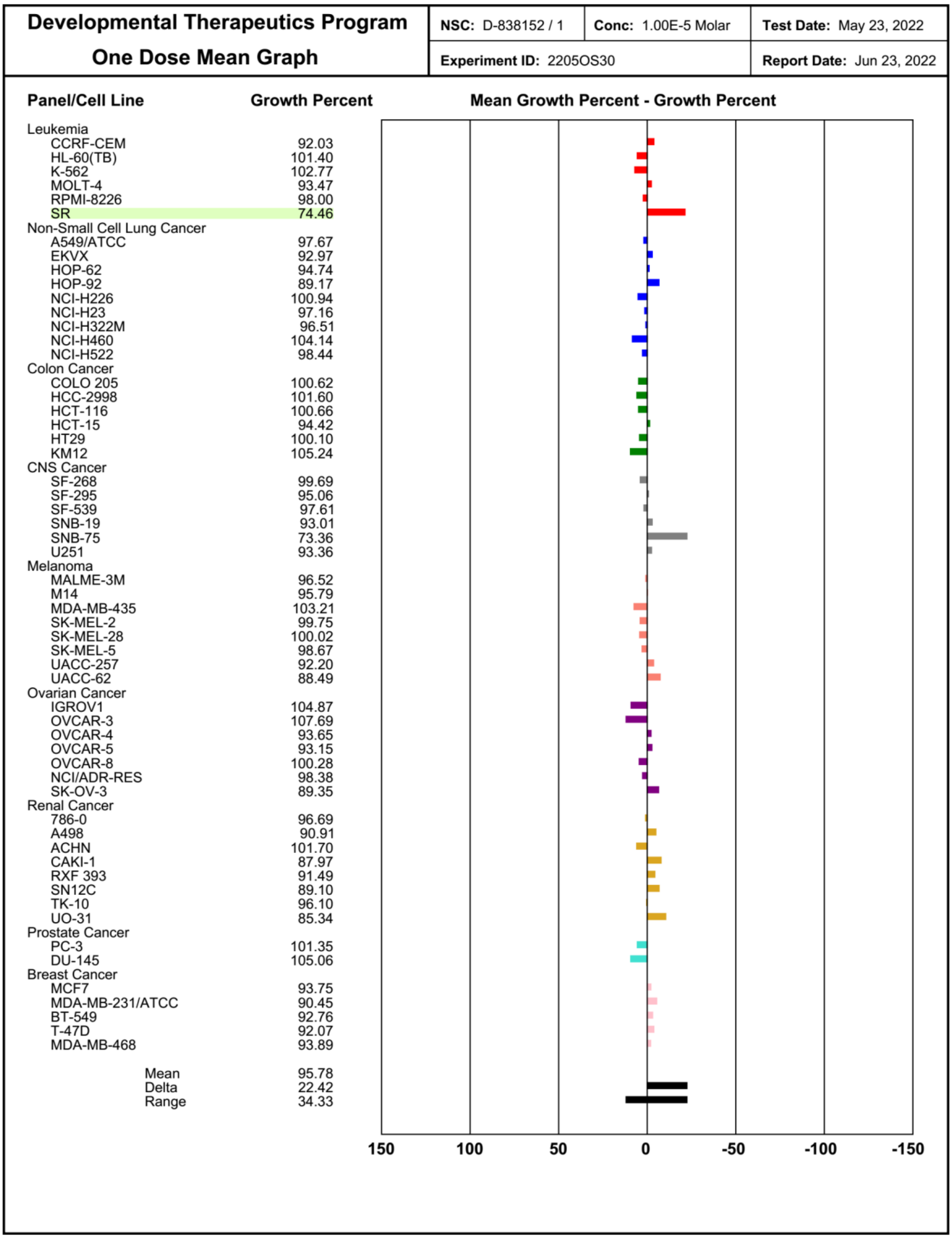

Figure S 46. One dose mean graph of **6e**.

3.6 6f

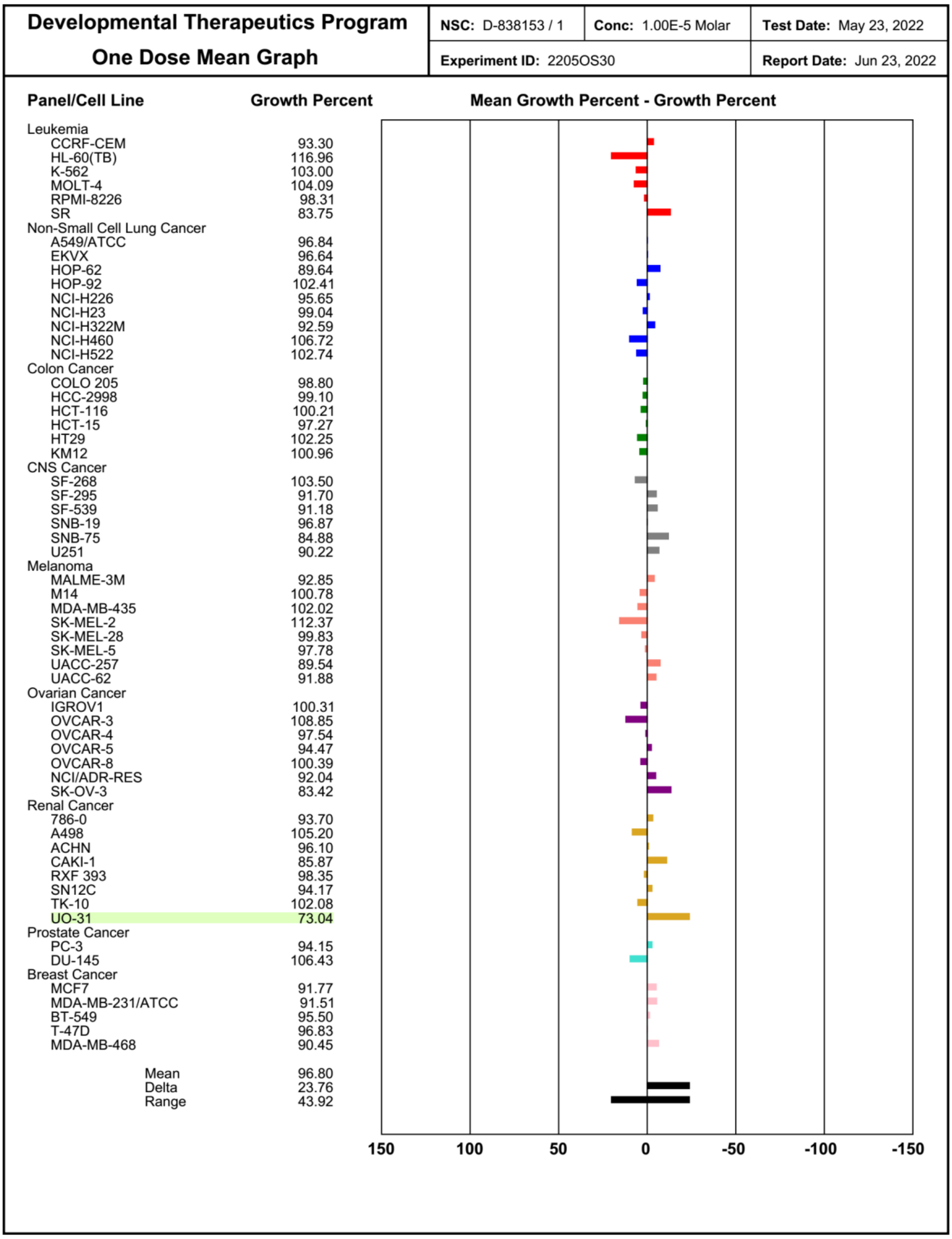

Figure S 47. One dose mean graph of **6f**.

3.7 6g

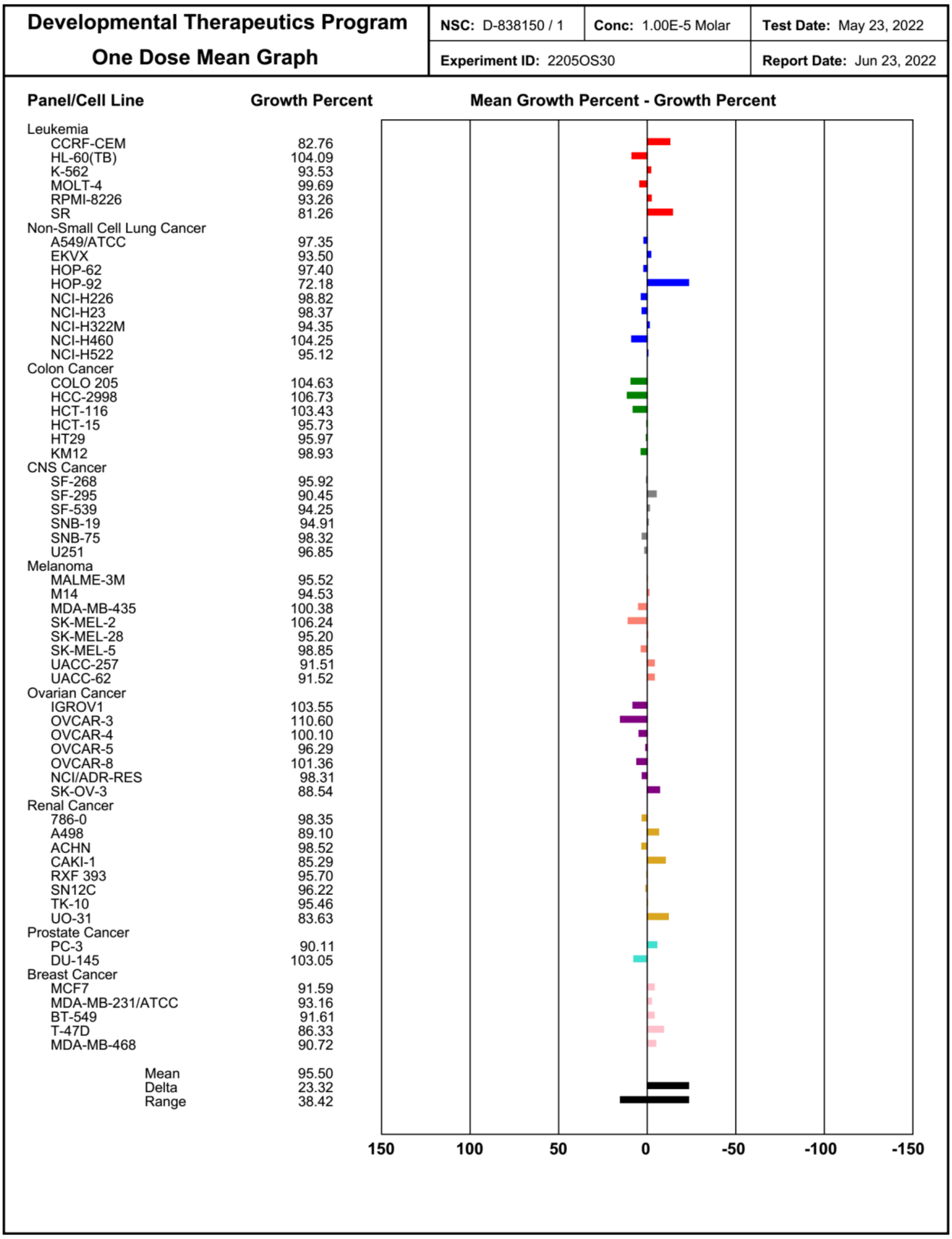

Figure S 48. One dose mean graph of **6g**.

3.8 6h

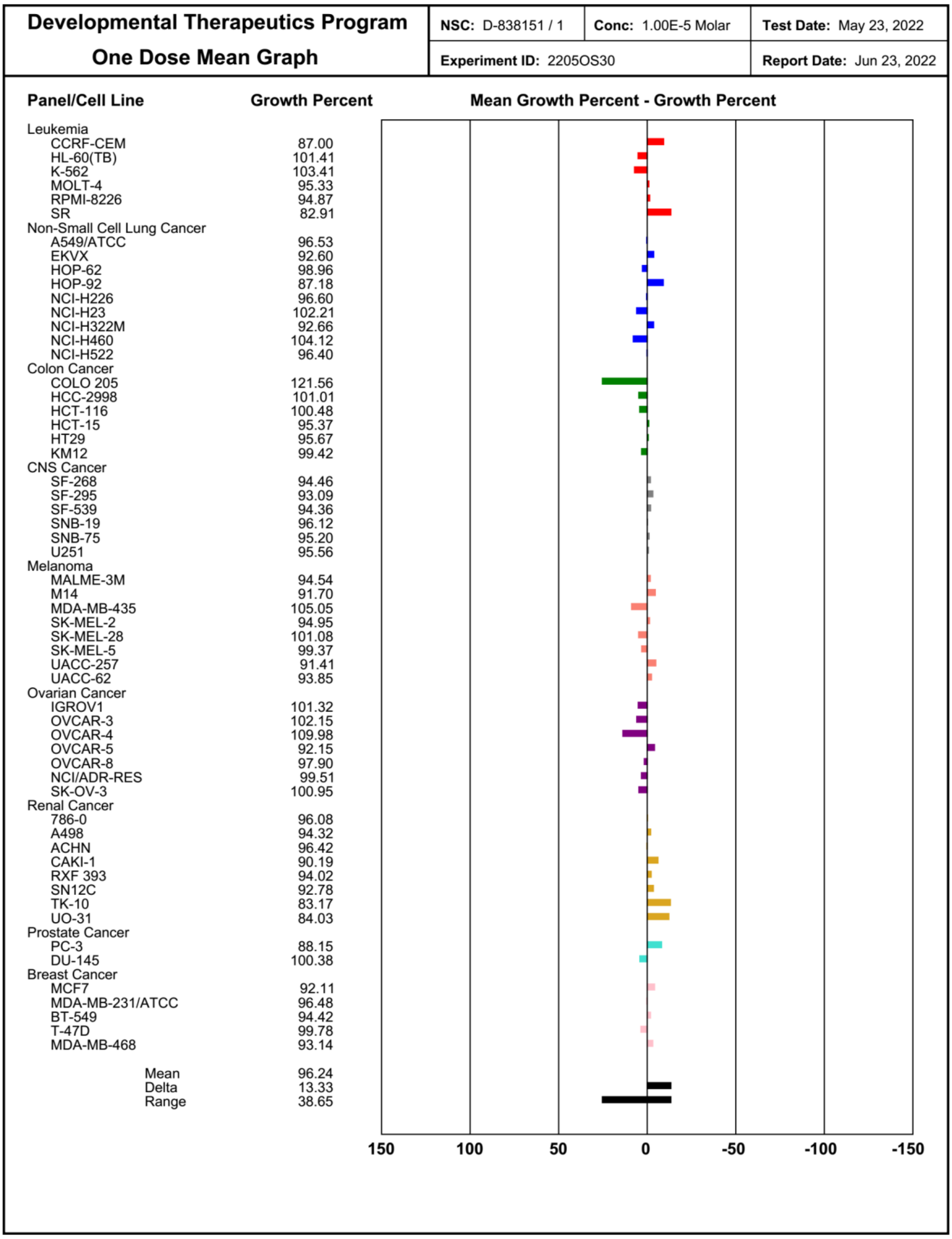

Figure S 49. One dose mean graph of **6h**.

3.9 6i

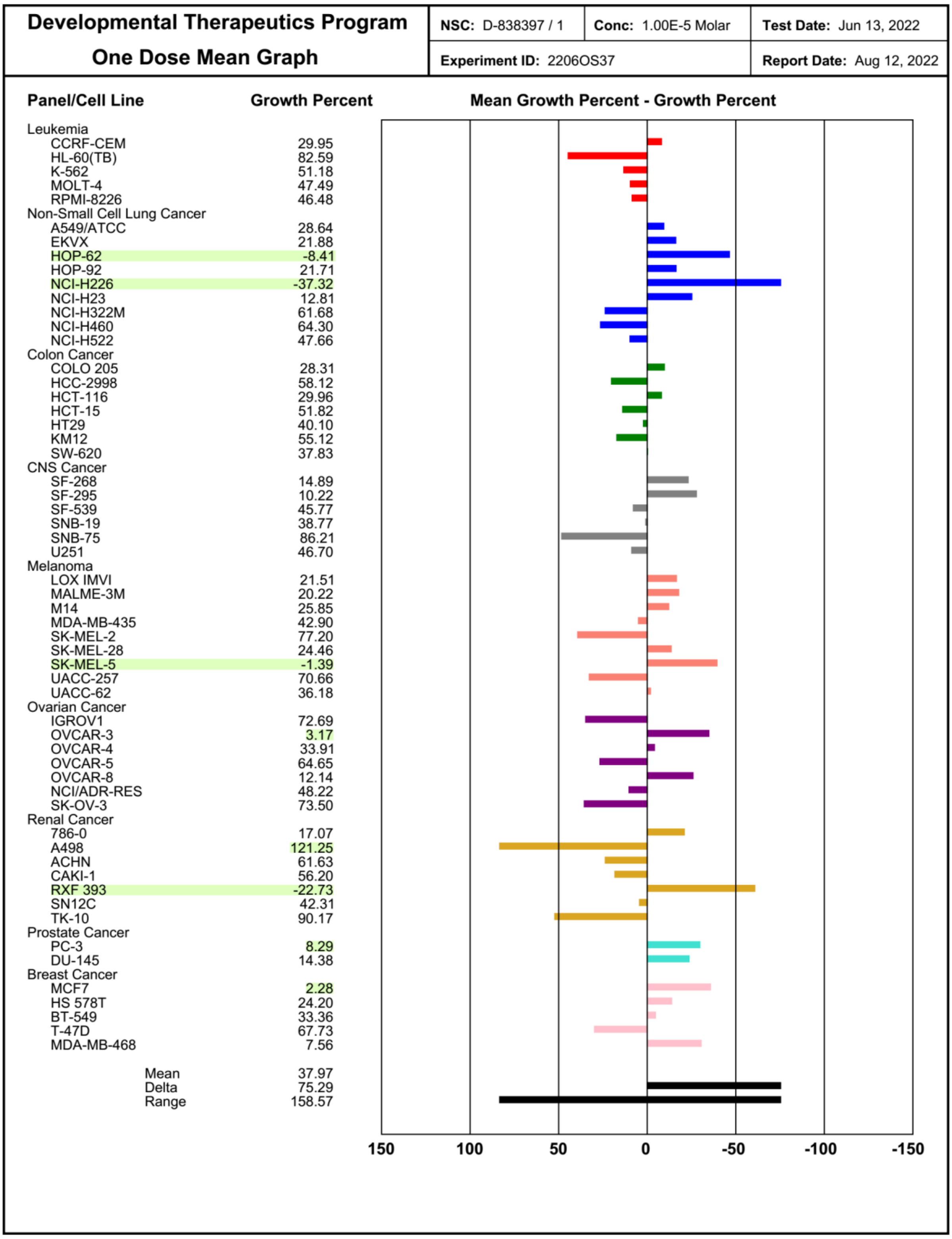

Figure S 50. One dose mean graph of **6i**.

3.10 6j

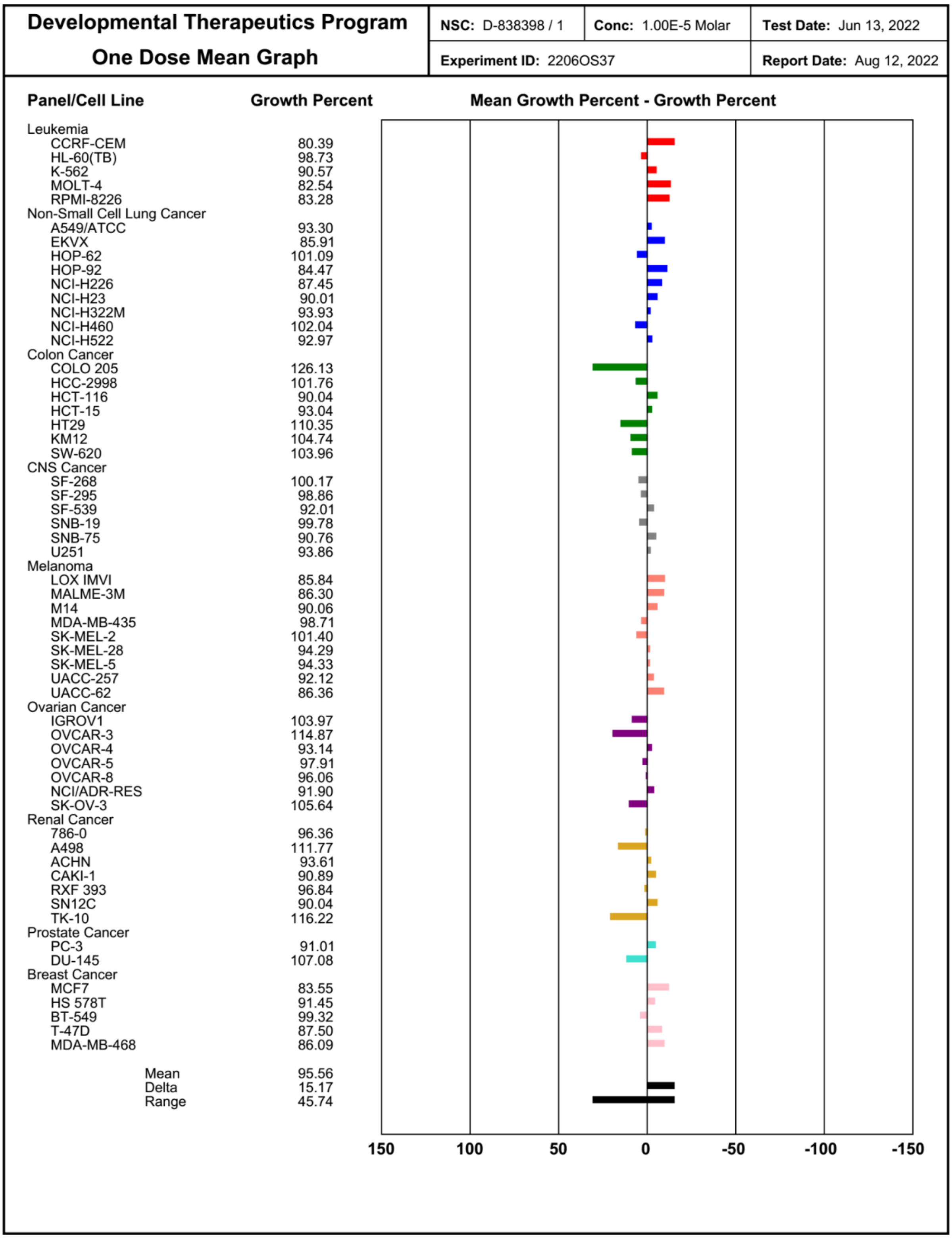

Figure S 51. One dose mean graph of **6j**.

3.11 6k

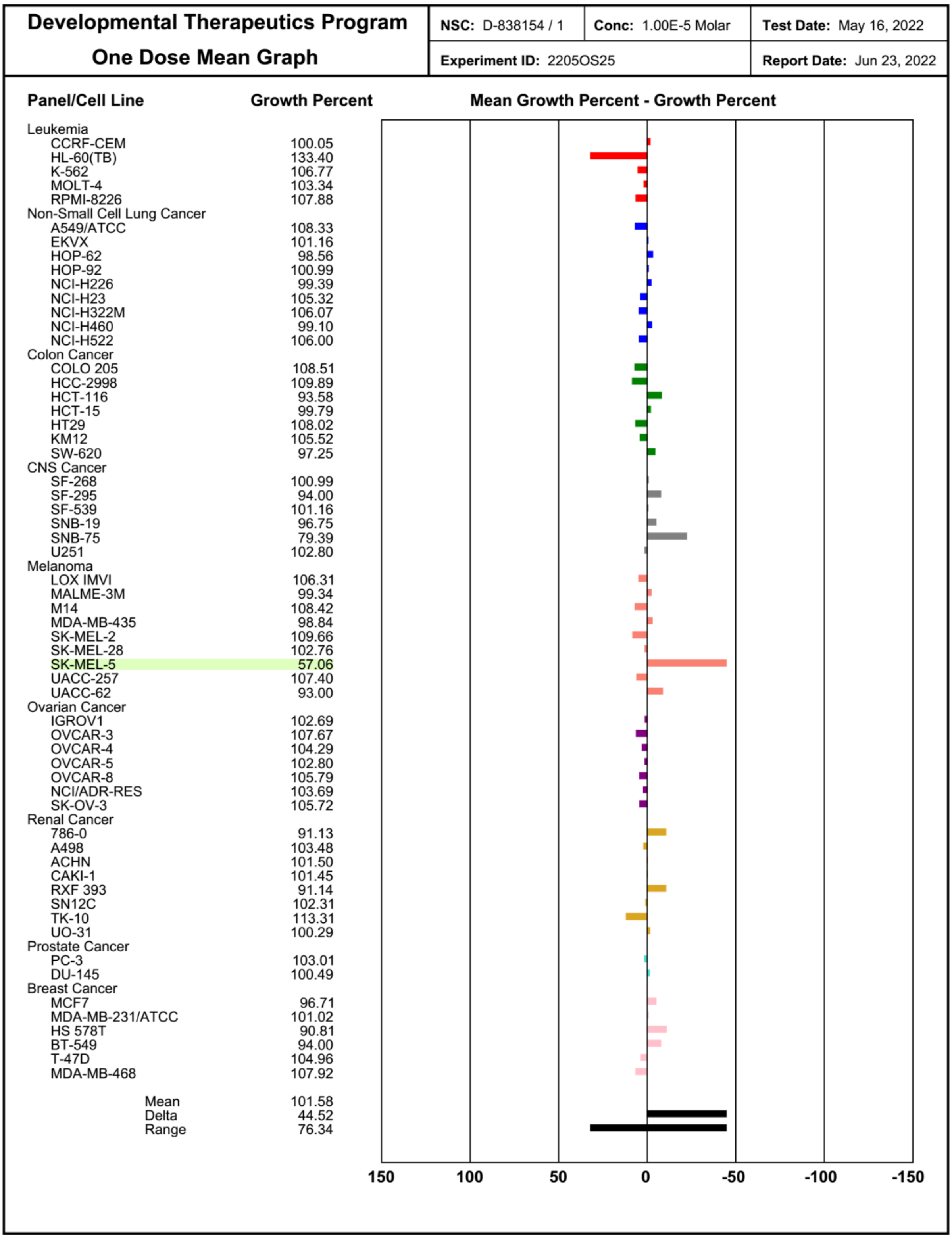

Figure S 52. One dose mean graph of **6k**.

3.12 6l

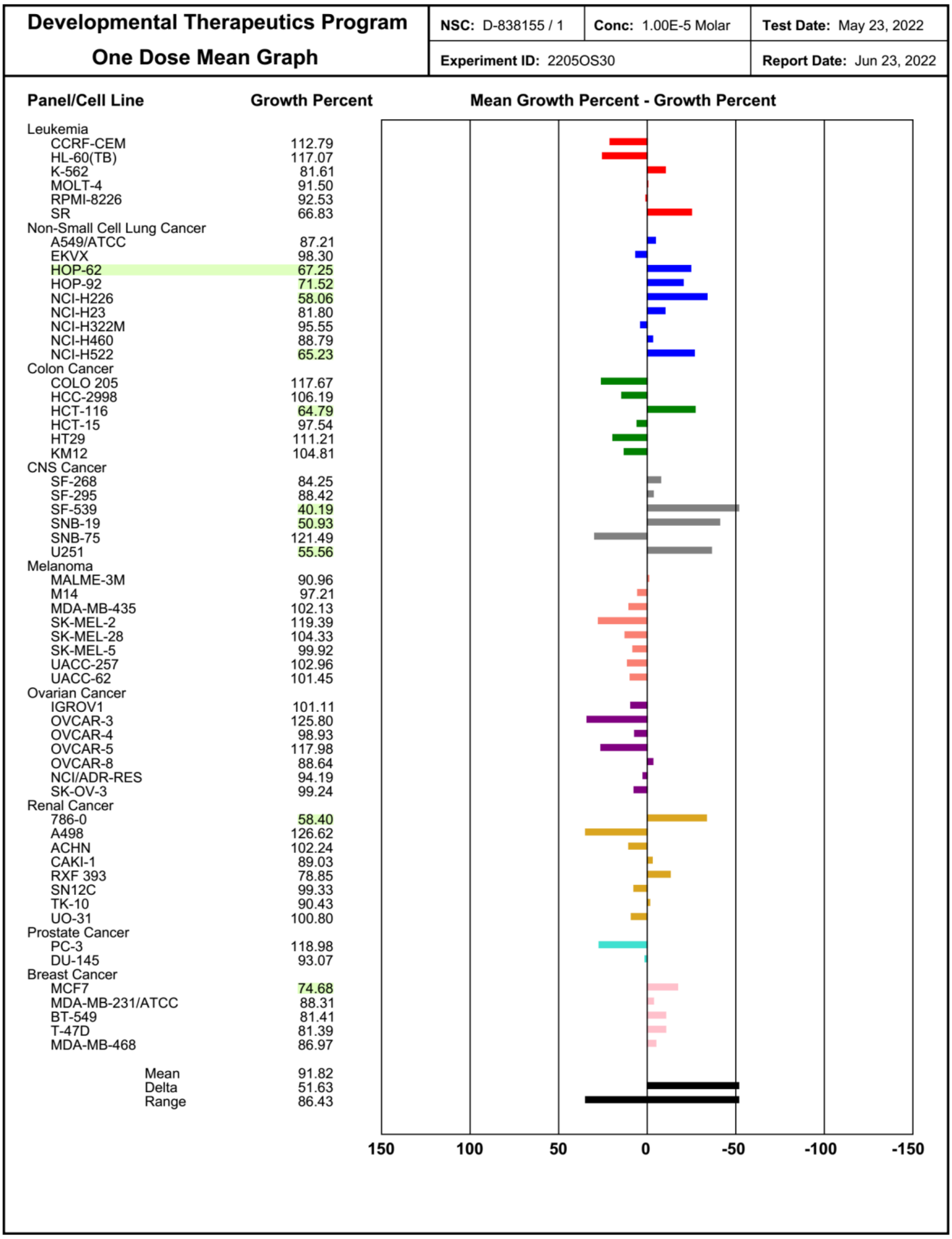

Figure S 53. One dose mean graph of **6l**.

3.13 7a

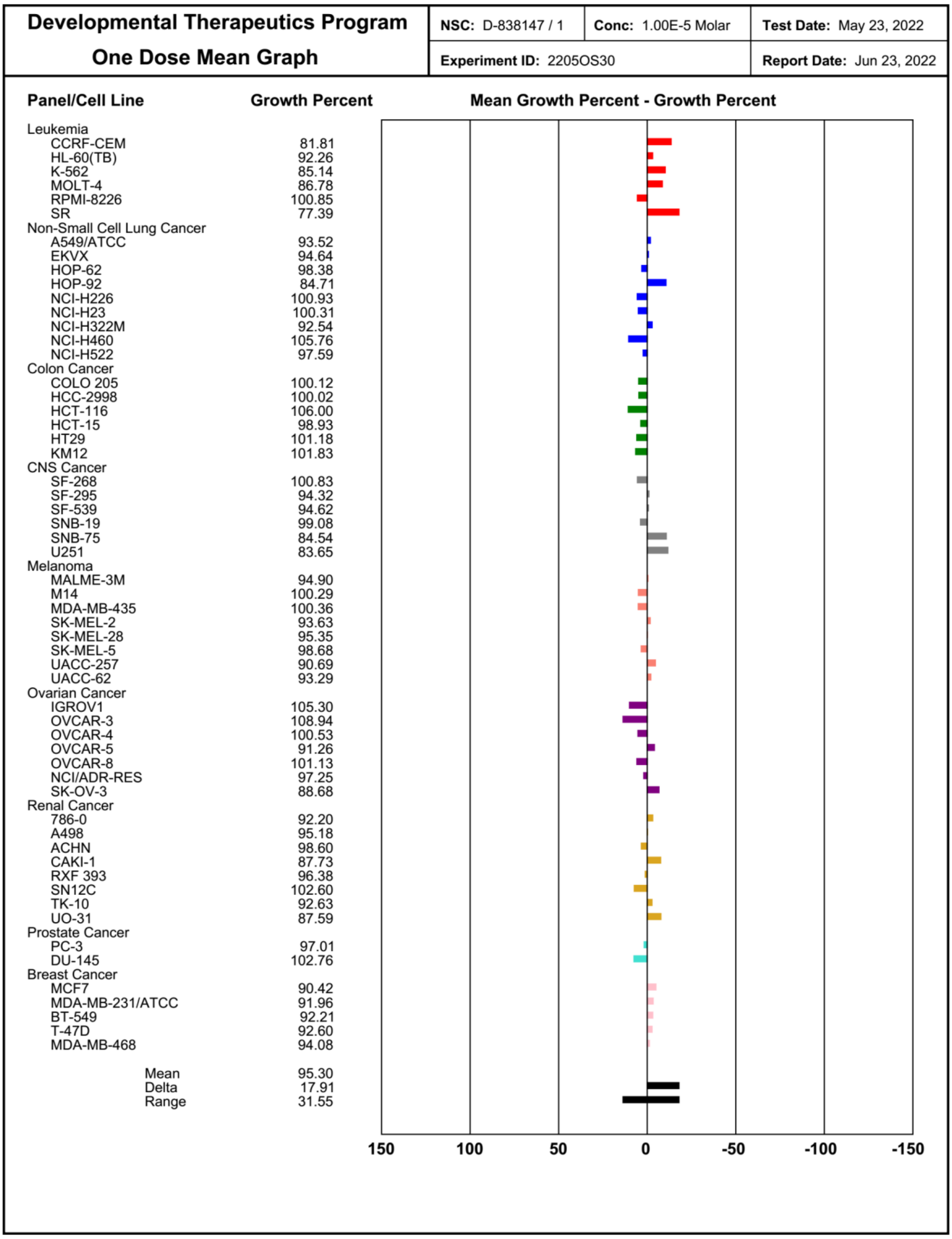

Figure S 54. One dose mean graph of **7a**.

3.14 7b

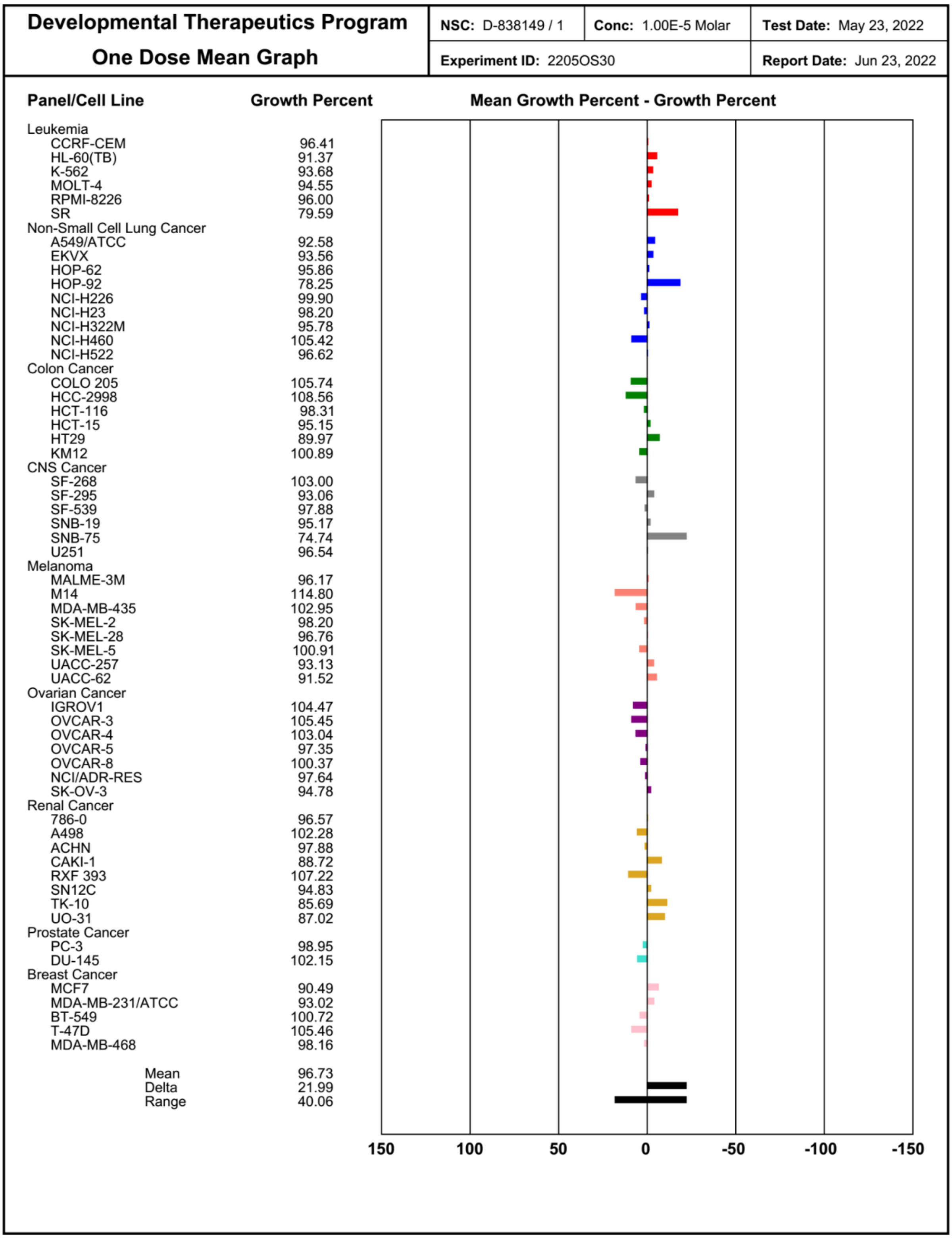

Figure S 55. One dose mean graph of **7b**.

3.15 9a

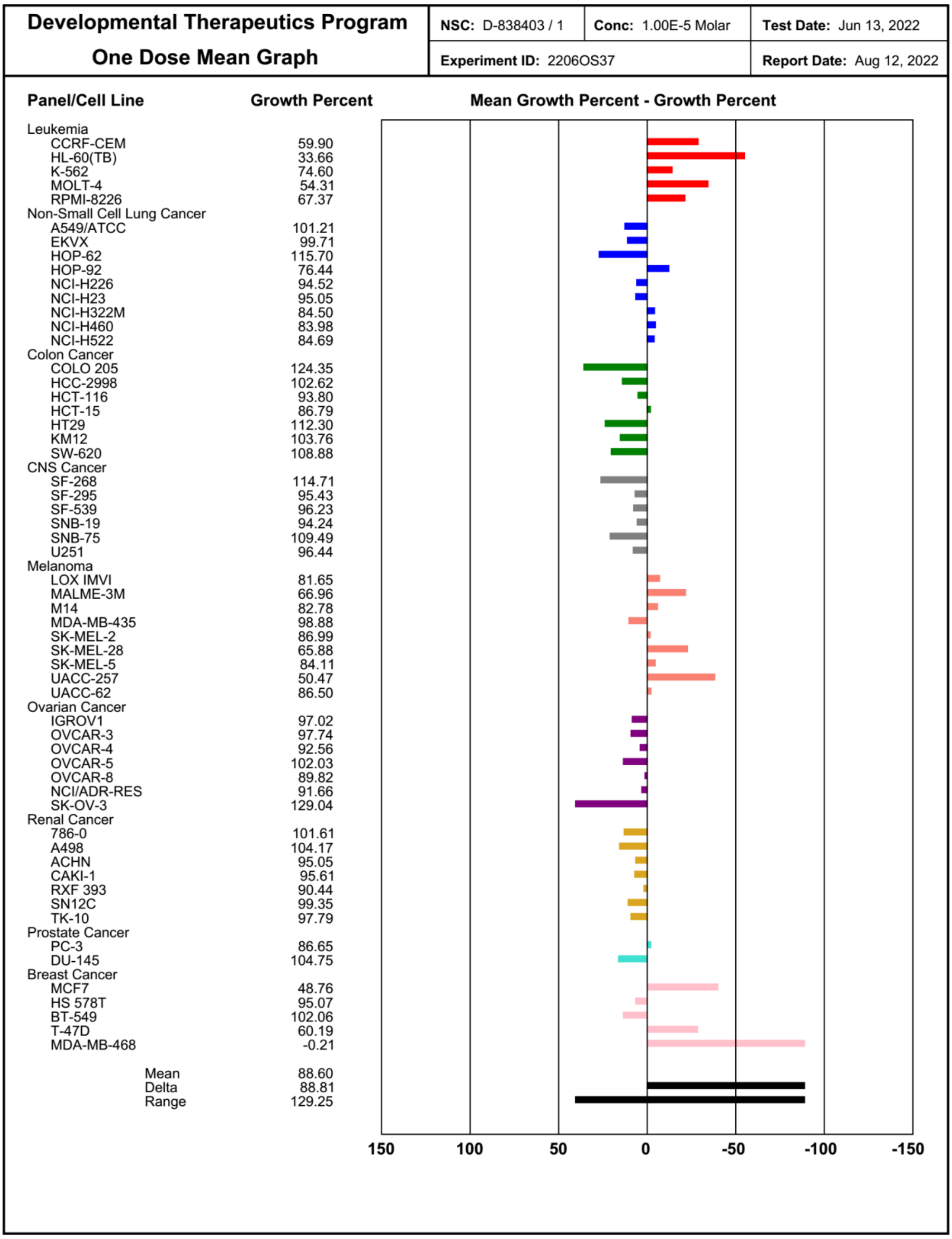

Figure S 56. One dose mean graph of **8a**.

3.16 9b

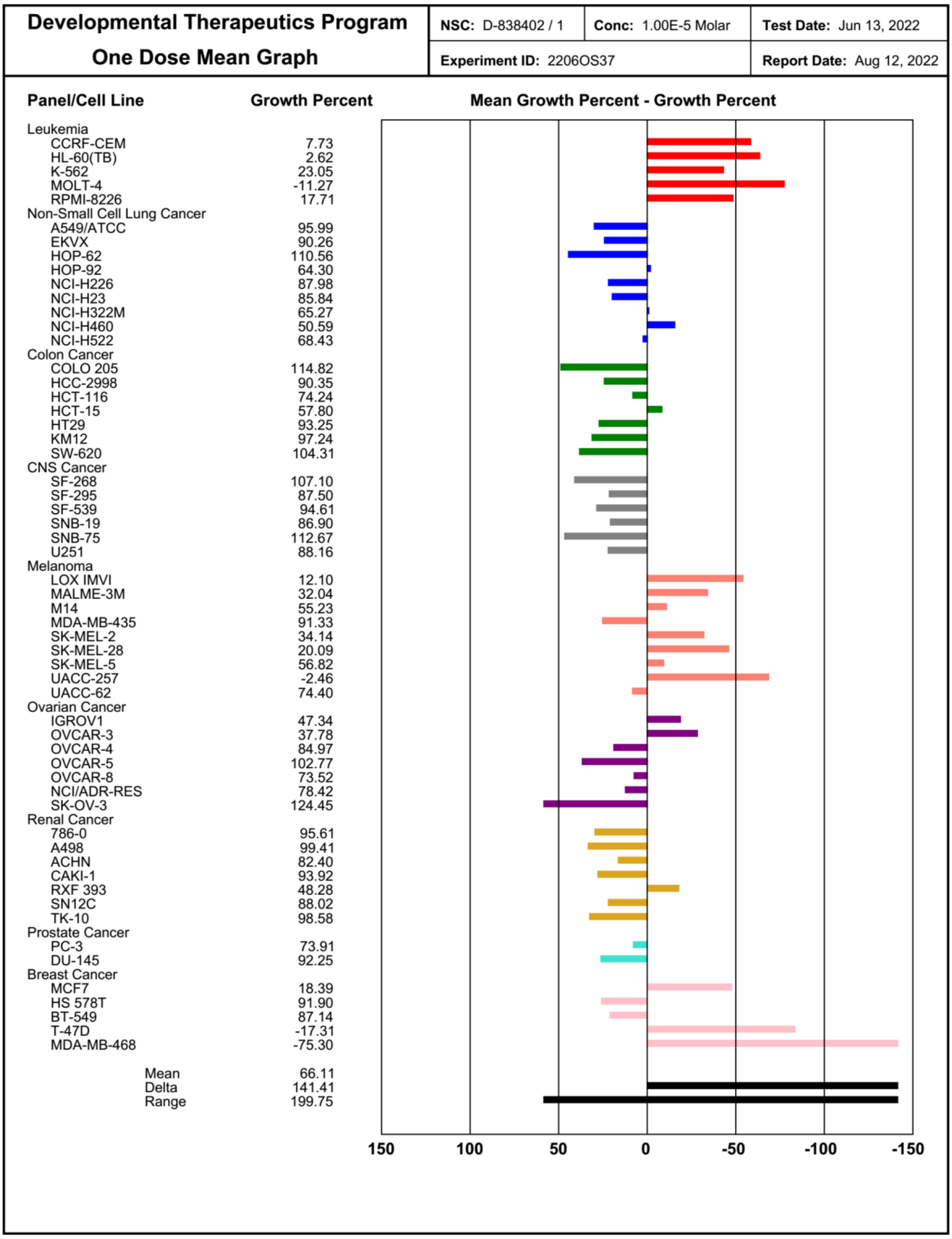

Figure S 57. One dose mean graph of **8b**.

3.17 9c

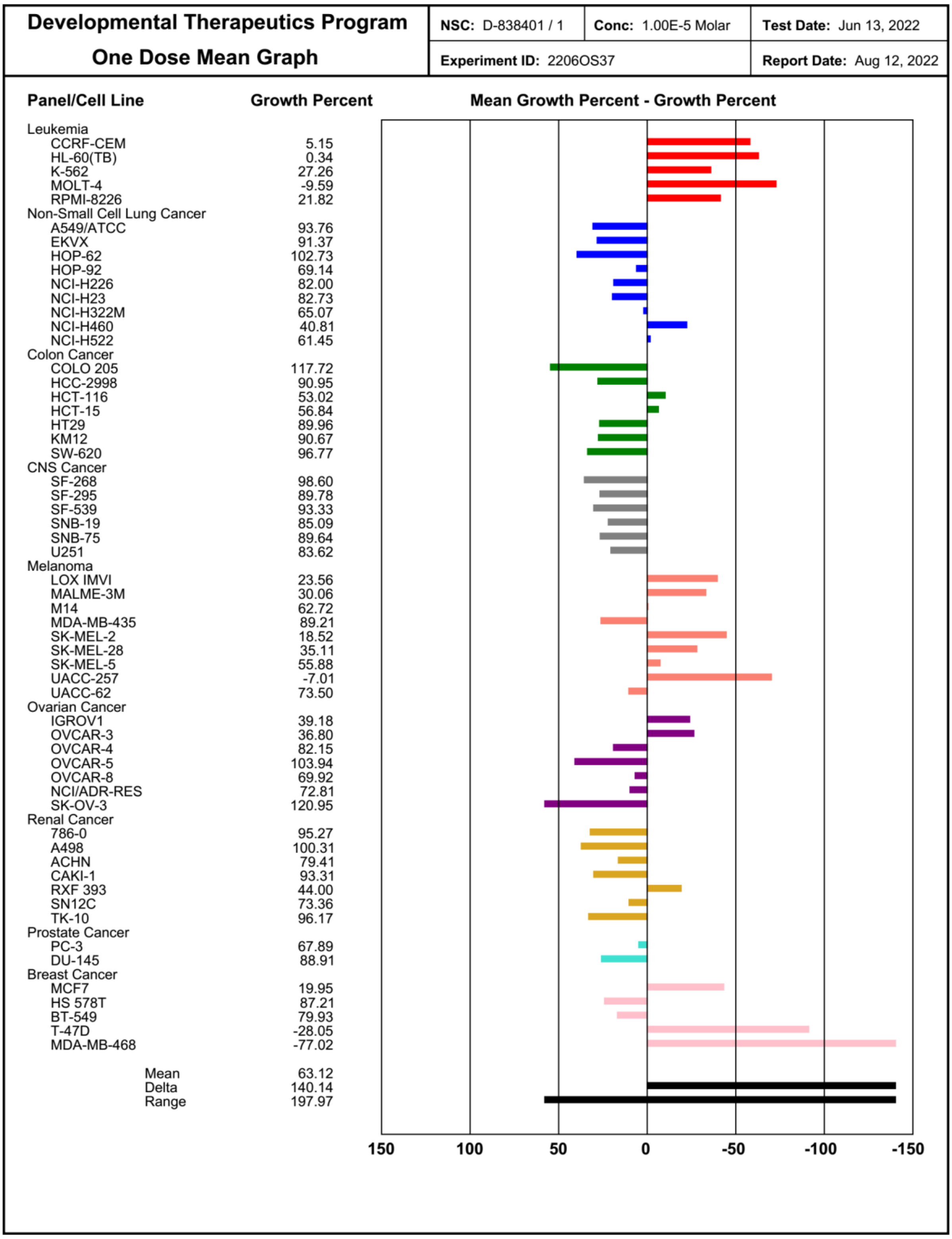

Figure S 58. One dose mean graph of **8c**.

4 NCI-60 cell line screening; Five-Dose Screen

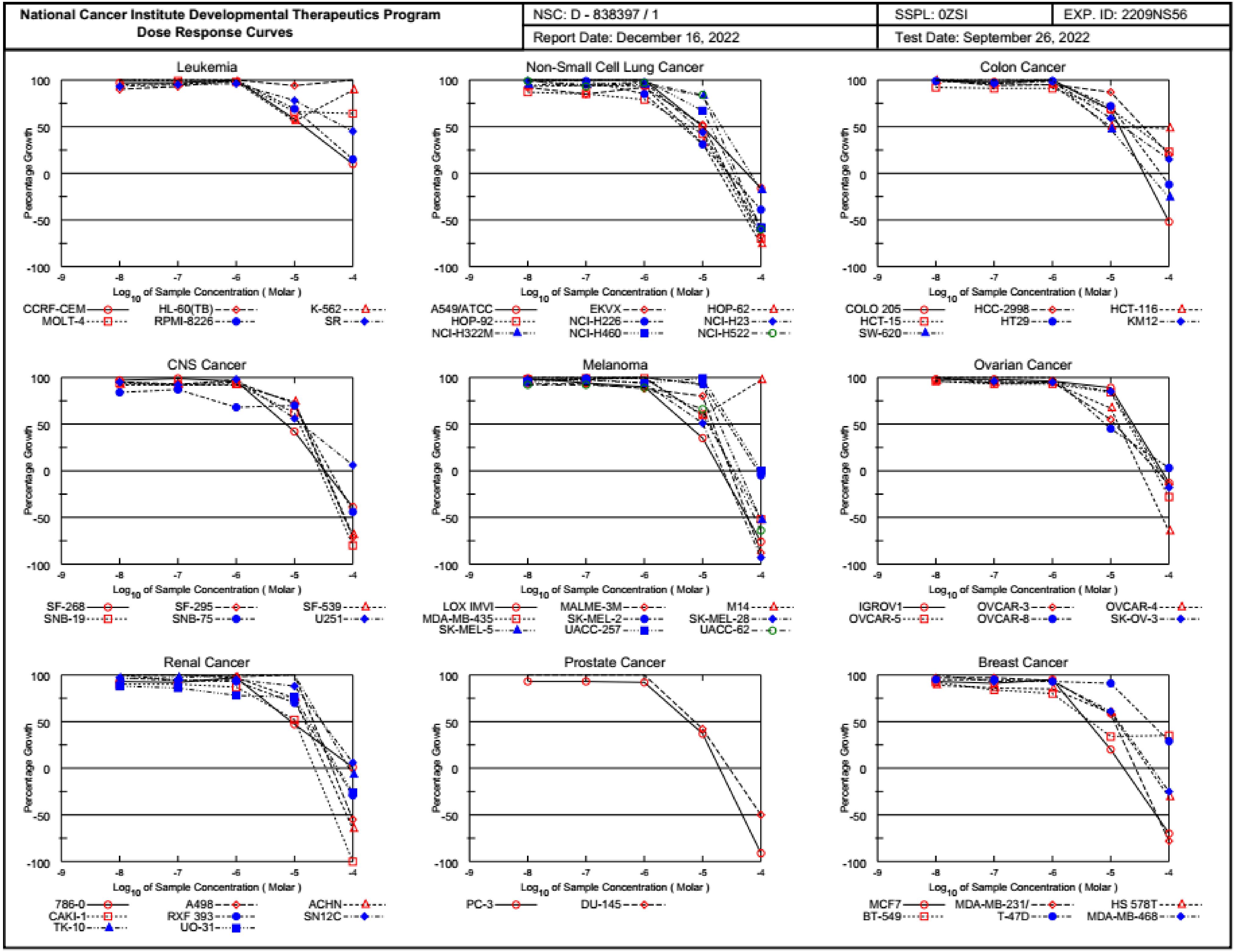

Figure S 59. *In vitro* five-dose testing: Dose response curves of compound **6i** against nine types of cancers.

National Cancer Institute Developmental Therapeutics Program
In-Vitro Testing Results

NSC : D - 838397 / 1	Experiment ID : 2209NS56	Test Type : 08	Units : Molar
Report Date : December 16, 2022	Test Date : September 26, 2022	QNS :	MC :
COMI : DHF-18	Stain Reagent : SRB Dual-Pass Related	SSPL : 0ZSI	

			Log10 Concentration												
	Time		Mean Optical Densities					Percent Growth							
Panel/Cell Line	Zero	Ctrl	-8.0	-7.0	-6.0	-5.0	-4.0	-8.0	-7.0	-6.0	-5.0	-4.0	GI50	TGI	LC50
**Leukemia**															
CCRF-CEM	0.400	1.895	1.852	1.847	1.922	1.264	0.543	97	97	102	58	10	1.45E-5	> 1.00E-4	> 1.00E-4
HL-60(TB)	0.429	1.826	1.684	1.723	1.871	1.737	1.942	90	93	103	94	108	> 1.00E-4	> 1.00E-4	> 1.00E-4
K-562	0.188	1.707	1.637	1.640	1.684	1.046	1.547	95	96	98	56	89	> 1.00E-4	> 1.00E-4	> 1.00E-4
MOLT-4	0.400	1.816	1.844	1.796	1.784	1.328	1.305	102	99	98	66	64	> 1.00E-4	> 1.00E-4	> 1.00E-4
RPMI-8226	0.462	2.401	2.412	2.477	2.392	1.808	0.750	101	104	100	69	15	2.27E-5	> 1.00E-4	> 1.00E-4
SR	0.207	0.597	0.568	0.577	0.582	0.511	0.383	93	95	96	78	45	7.10E-5	> 1.00E-4	> 1.00E-4
**Non-Small Cell Lung Cancer**															
A549/ATCC	0.300	2.150	2.196	2.250	2.233	1.217	0.253	102	105	104	50	-16	9.81E-6	5.73E-5	> 1.00E-4
EKVX	0.620	1.952	1.848	1.753	1.845	1.314	0.203	92	85	92	52	-67	1.04E-5	2.73E-5	7.17E-5
HOP-62	0.629	2.283	2.161	2.186	2.175	1.160	0.151	93	94	93	32	-76	5.11E-6	1.98E-5	5.75E-5
HOP-92	1.277	1.758	1.697	1.687	1.655	1.479	0.383	87	85	79	42	-70	6.01E-6	2.37E-5	6.62E-5
NCI-H226	0.708	1.842	1.882	1.828	1.677	1.065	0.429	104	99	85	31	-39	4.54E-6	2.78E-5	> 1.00E-4
NCI-H23	0.490	1.692	1.681	1.630	1.674	1.014	0.202	99	95	98	44	-59	7.64E-6	2.67E-5	8.21E-5
NCI-H322M	0.801	2.122	2.058	2.053	2.062	1.900	0.659	95	95	95	83	-18	2.13E-5	6.66E-5	> 1.00E-4
NCI-H460	0.224	2.073	2.372	2.239	2.169	1.460	0.095	116	109	105	67	-58	1.37E-5	3.44E-5	8.69E-5
NCI-H522	0.898	2.528	2.515	2.416	2.480	2.260	0.358	99	93	97	84	-60	1.71E-5	3.82E-5	8.50E-5
**Colon Cancer**															
COLO 205	0.562	2.208	2.340	2.176	2.310	1.676	0.269	108	98	106	68	-52	1.40E-5	3.67E-5	9.60E-5
HCC-2998	1.039	3.180	3.195	3.057	3.078	2.891	1.458	101	94	95	87	20	3.51E-5	> 1.00E-4	> 1.00E-4
HCT-116	0.260	2.414	2.392	2.332	2.302	1.331	1.287	99	96	95	50	48	9.85E-6	> 1.00E-4	> 1.00E-4
HCT-15	0.372	2.528	2.358	2.338	2.338	1.858	0.875	92	91	91	69	23	2.60E-5	> 1.00E-4	> 1.00E-4
HT29	0.484	2.656	2.630	2.585	2.634	2.055	0.425	99	97	99	72	-12	1.84E-5	7.17E-5	> 1.00E-4
KM12	0.473	2.425	2.526	2.353	2.408	1.627	0.768	105	96	99	59	15	1.61E-5	> 1.00E-4	> 1.00E-4
SW-620	0.330	2.119	2.225	2.128	2.145	1.180	0.245	106	100	101	47	-26	8.98E-6	4.44E-5	> 1.00E-4
**CNS Cancer**															
SF-268	0.665	2.268	2.224	2.250	2.204	1.332	0.405	97	99	96	42	-39	7.01E-6	3.28E-5	> 1.00E-4
SF-295	0.856	2.781	2.685	2.648	2.687	2.238	0.251	95	93	95	72	-71	1.42E-5	3.19E-5	7.16E-5
SF-539	0.766	2.438	2.301	2.306	2.300	2.011	0.237	92	92	92	74	-69	1.48E-5	3.30E-5	7.36E-5
SNB-19	0.521	1.906	1.829	1.794	1.803	1.377	0.104	94	92	93	62	-80	1.21E-5	2.73E-5	6.13E-5
SNB-75	1.299	2.260	2.111	2.136	1.952	1.970	0.723	84	87	68	70	-44	1.49E-5	4.09E-5	> 1.00E-4
U251	0.284	1.887	1.808	1.763	1.850	1.184	0.375	95	92	98	56	6	1.32E-5	> 1.00E-4	> 1.00E-4
**Melanoma**															
LOX IMVI	0.198	1.384	1.377	1.316	1.271	0.608	0.048	99	94	90	35	-76	5.30E-6	2.06E-5	5.84E-5
MALME-3M	0.681	1.389	1.342	1.339	1.305	1.245	0.082	93	93	88	80	-88	1.50E-5	2.99E-5	5.94E-5
M14	0.451	1.852	1.816	1.823	1.886	1.281	1.815	97	98	102	59	97	> 1.00E-4	> 1.00E-4	> 1.00E-4
MDA-MB-435	0.595	2.510	2.468	2.494	2.493	1.741	0.286	98	99	99	60	-52	1.22E-5	3.43E-5	9.61E-5
SK-MEL-2	1.479	2.877	2.836	2.858	2.871	2.783	1.407	97	99	100	93	-5	2.76E-5	8.91E-5	> 1.00E-4
SK-MEL-28	0.488	1.486	1.467	1.462	1.425	0.993	0.035	98	98	94	51	-93	1.01E-5	2.25E-5	5.02E-5
SK-MEL-5	0.911	3.365	3.362	3.369	3.362	3.159	0.431	100	100	100	92	-53	1.94E-5	4.31E-5	9.58E-5
UACC-257	1.111	2.812	2.729	2.763	2.718	2.798	1.119	95	97	94	99	0	3.15E-5	> 1.00E-4	> 1.00E-4
UACC-62	0.783	2.609	2.458	2.469	2.407	1.990	0.280	92	92	89	66	-64	1.33E-5	3.21E-5	7.78E-5
**Ovarian Cancer**															
IGROV1	0.835	2.613	2.574	2.572	2.546	2.410	0.725	98	98	96	89	-13	2.39E-5	7.42E-5	> 1.00E-4
OVCAR-3	0.555	1.876	1.927	1.803	1.815	1.280	0.473	104	94	95	55	-15	1.17E-5	6.13E-5	> 1.00E-4
OVCAR-4	1.008	2.187	2.143	2.117	2.117	1.796	0.351	96	94	94	67	-65	1.34E-5	3.21E-5	7.67E-5
OVCAR-5	0.709	1.597	1.562	1.530	1.531	1.458	0.511	96	93	93	84	-28	2.02E-5	5.63E-5	> 1.00E-4
OVCAR-8	0.464	2.568	2.674	2.584	2.572	1.414	0.532	105	101	100	45	3	8.17E-6	> 1.00E-4	> 1.00E-4
SK-OV-3	0.994	2.231	2.311	2.186	2.170	2.042	0.819	106	96	95	85	-18	2.18E-5	6.73E-5	> 1.00E-4
**Renal Cancer**															
786-0	0.583	2.371	2.254	2.220	2.312	1.422	0.594	93	92	97	47	1	8.66E-6	> 1.00E-4	> 1.00E-4
A498	1.209	1.829	1.852	1.879	1.819	1.947	0.539	104	108	98	119	-55	2.49E-5	4.81E-5	9.30E-5
ACHN	0.375	1.705	1.734	1.747	1.670	1.370	0.132	102	103	97	75	-65	1.51E-5	3.43E-5	7.83E-5
CAKI-1	0.550	2.182	2.014	2.019	1.966	1.406	-0.004	90	90	87	52	-100	1.04E-5	2.21E-5	4.70E-5
RXF 393	0.898	1.816	1.814	1.761	1.755	1.540	0.638	100	94	93	70	-29	1.59E-5	5.09E-5	> 1.00E-4
SN12C	1.086	3.246	3.185	3.117	3.133	2.993	1.215	97	94	95	88	6	2.92E-5	> 1.00E-4	> 1.00E-4
TK-10	1.345	2.905	2.836	2.854	2.965	3.005	1.246	96	97	104	106	-7	3.13E-5	8.62E-5	> 1.00E-4
UO-31	0.743	2.292	2.111	2.078	1.951	1.924	0.552	88	86	78	76	-26	1.81E-5	5.59E-5	> 1.00E-4
**Prostate Cancer**															
PC-3	0.557	2.328	2.196	2.196	2.188	1.210	0.050	93	93	92	37	-91	5.79E-6	1.94E-5	4.77E-5
DU-145	0.455	2.045	2.098	2.091	2.095	1.128	0.229	103	103	103	42	-50	7.47E-6	2.88E-5	> 1.00E-4
**Breast Cancer**															
MCF7	0.401	2.241	2.117	2.070	2.140	0.761	0.119	93	91	95	20	-70	3.93E-6	1.65E-5	5.94E-5
MDA-MB-231/ATCC	0.528	1.269	1.249	1.247	1.227	0.960	0.117	97	97	94	58	-78	1.15E-5	2.68E-5	6.25E-5
HS 578T	1.518	2.555	2.444	2.415	2.404	2.133	1.055	89	86	85	59	-31	1.27E-5	4.57E-5	> 1.00E-4
BT-549	1.169	2.168	2.089	2.004	1.971	1.511	1.522	92	84	80	34	35	4.54E-6	> 1.00E-4	> 1.00E-4
T-47D	1.830	3.338	3.269	3.262	3.239	3.202	2.264	95	95	93	91	29	4.56E-5	> 1.00E-4	> 1.00E-4
MDA-MB-468	0.688	1.755	1.780	1.686	1.686	1.340	0.517	102	93	93	61	-25	1.35E-5	5.13E-5	> 1.00E-4

Figure S 60. *In vitro* five-dose testing: GI_{50}, TGI and LC_{50} parameters for compound **6i.**

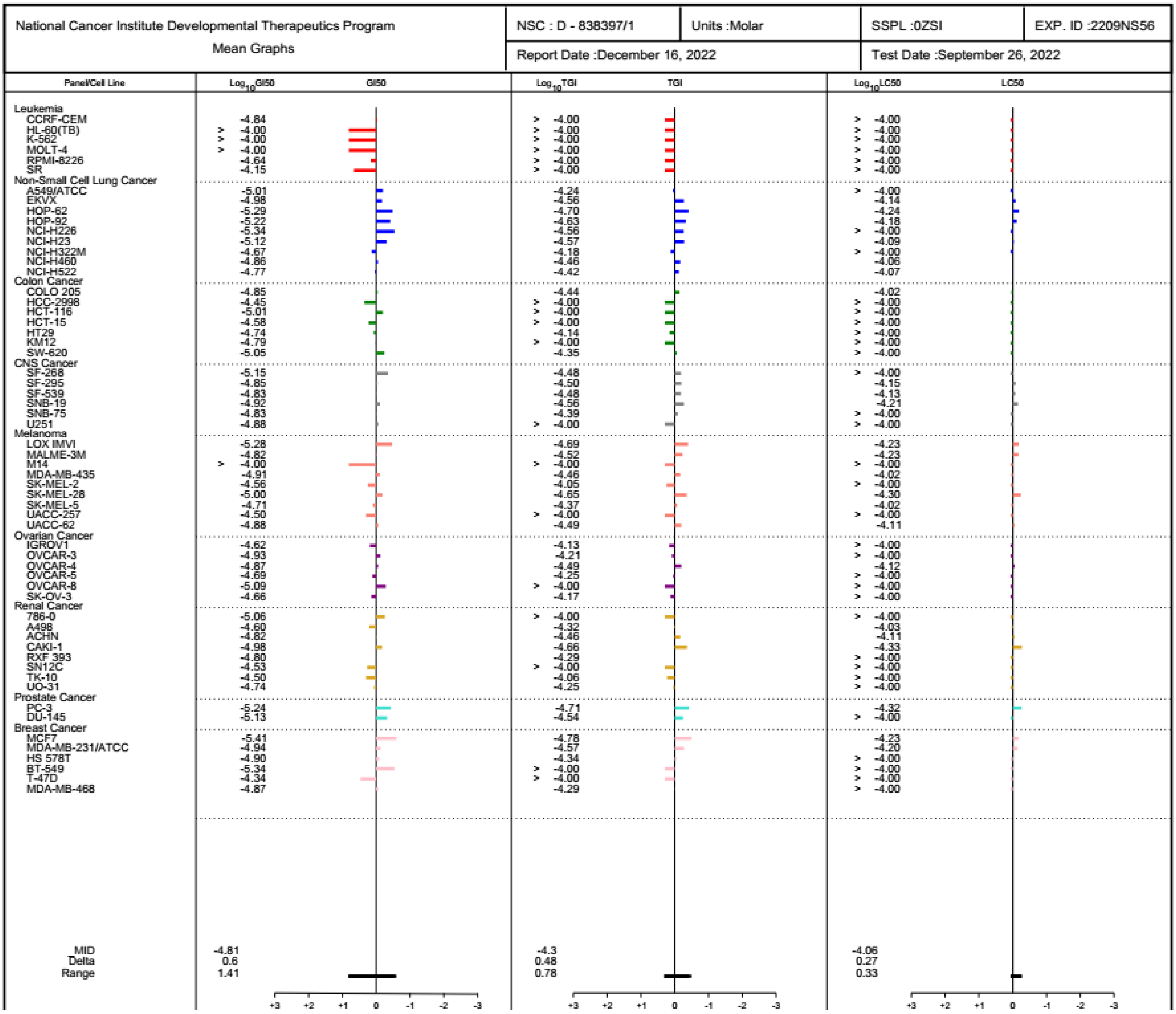

Figure S 61. *In vitro* five-dose testing: Mean graphs of compound **6i.**

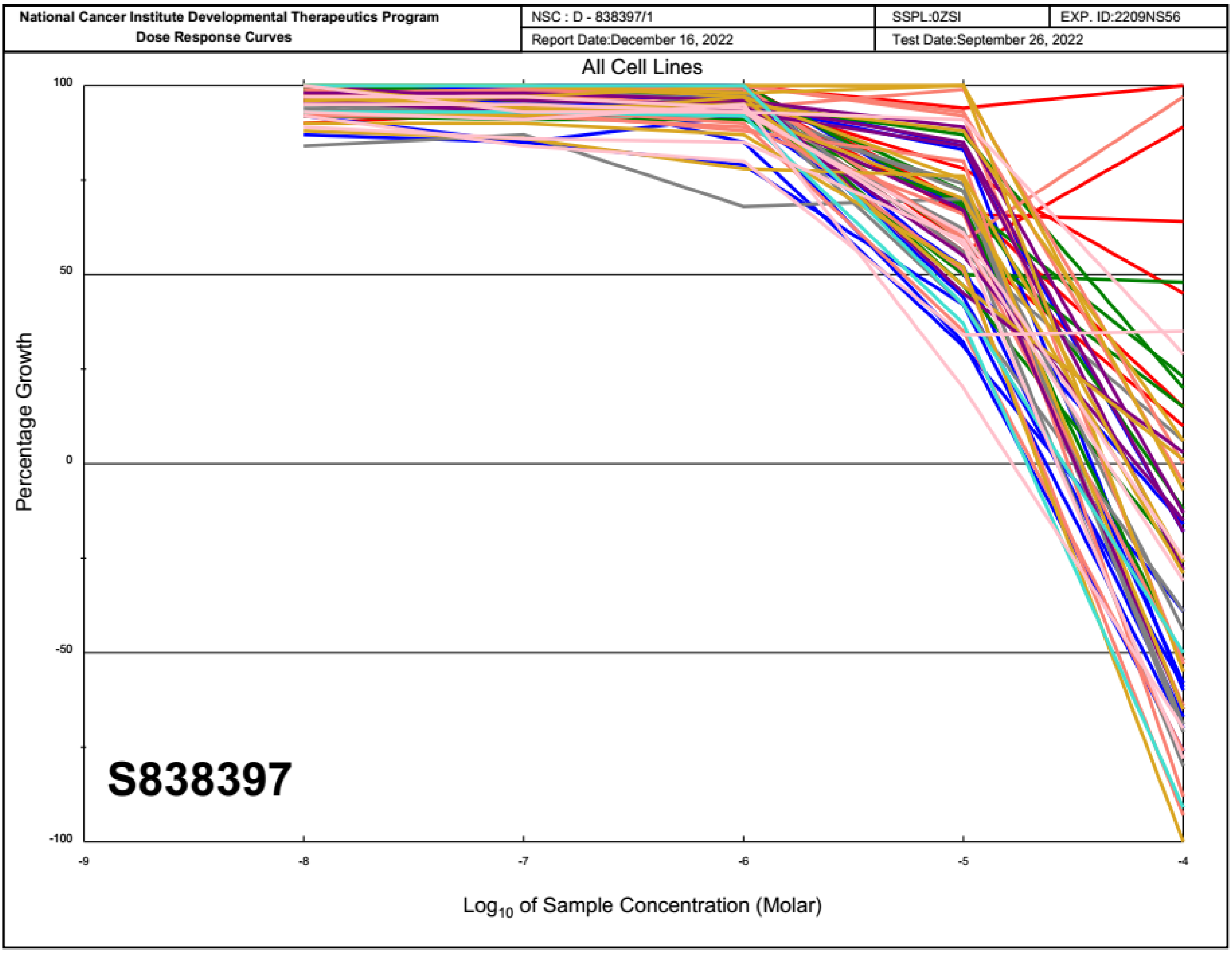

Figure S 62. *In vitro* five-dose testing: Dose response curves (all cell lines) for compound **6i.**

5 DHFR assay graphs for both 6i and MTX

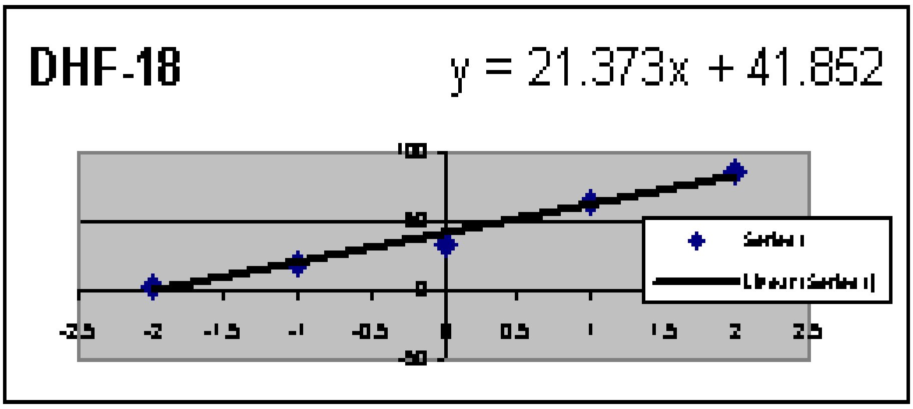

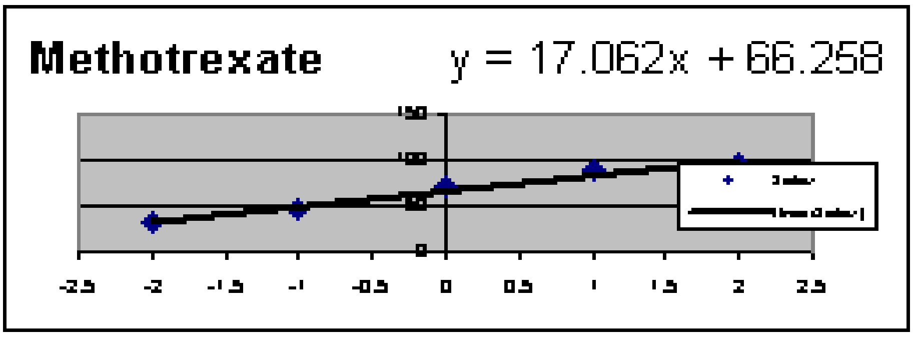

6 TS assay graph for both 6i and 5-FU

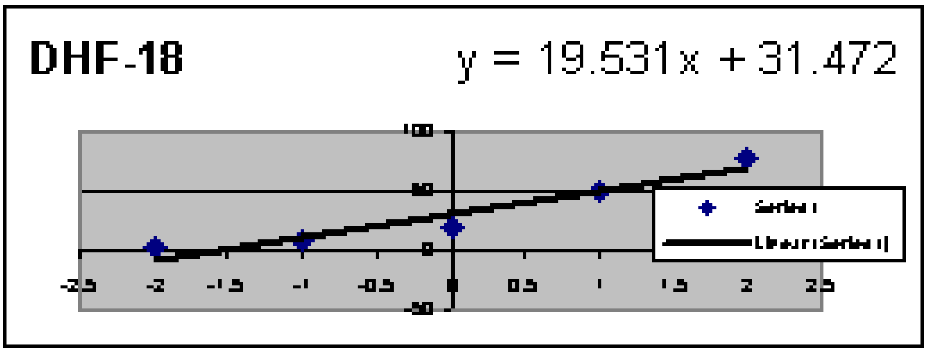

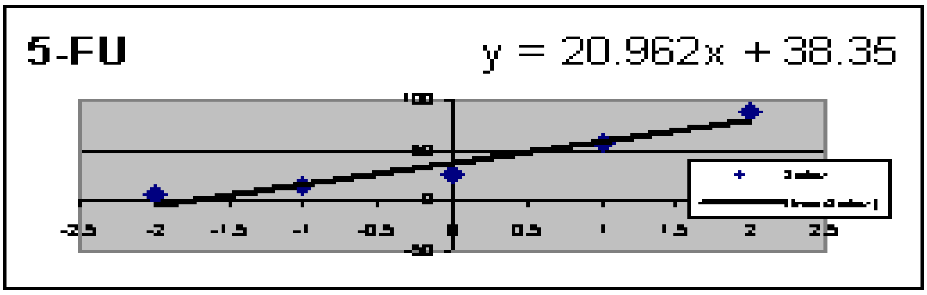

7 Experimental Chemistry

7.1 *1-(4-fluorophenyl)-3,6-dimethyl-1,5-dihydro-4H-pyrazolo[3,4-d]pyrimidin-4-one (**3f**)*

White crytals; 69% yield; hexane; Melting point: >300 °C; ^{1}H NMR (400 MHz, DMSO-*d*$_6$) δ 12.25 (s, 1H, amide NH), 8.05 (dd, *J* = 9.0, 4.9 Hz, 2H, H-Ar), 7.37 (t, *J* = 8.8 Hz, 2H, H-Ar), 2.51 (s, 3H, 3-CH_3), 2.38 (s, 3H, 6-CH_3); ^{13}C NMR (101 MHz, DMSO) δ 159.26, 159.01, 153.27, 146.20, 135.33, 123.70, 116.45, 116.22, 104.10, 21.93, 13.75; Anal. Calcd. for $C_{13}H_{11}FN_4O$ (258.2564): C, 60.46; H, 4.29; N, 21.69; Found: C, 60.61; H, 4.37; N, 21.85.

7.2 *Diethyl (4-(2-(4-oxo-1-phenyl-1,4-dihydro-5H-pyrazolo[3,4-d]pyrimidin-5-yl)acetamido)benzoyl)-L-glutamate (**6a**)*

Off-white powder; 60% yield; aqueous ethanol 50%; Melting point: 209-211 °C; ^{1}H NMR (400 MHz, DMSO-*d*$_6$) δ 10.76 (s, 1H, CH_2CONH), 8.66 (d, *J* = 7.4 Hz, 1H, CON*H*CH), 8.53 (s, 1H, C6-H), 8.43 (s, 1H, C3-H), 8.10 – 8.05 (m, 2H, H-Ar), 7.90 (d, *J* = 8.4 Hz, 2H, H-Ar), 7.70 (d, *J* = 8.5 Hz, 2H, H-Ar), 7.61 (t, *J* = 7.8 Hz, 2H, H-Ar), 7.45 (t, *J* = 7.4 Hz, 1H, H-Ar), 4.97 (s, 2H, CH_2CONH), 4.43 (ddd, *J* = 9.6, 7.2, 5.1 Hz, 1H, Cα-H), 4.16 – 4.02 (m, 4H, 2 x OCH_2CH_3), 2.46 (t, *J* = 7.5 Hz, 2H, Cγ-H), 2.18 – 2.07 (m, 1H, Cβ-Ha), 2.07 – 1.97 (m, 1H, Cβ-Hb), 1.19 (dt, *J* = 10.6, 7.1 Hz, 6H, 2 x OCH_2CH_3); ^{13}C NMR (101 MHz, DMSO-*d*$_6$) δ 172.69, 172.34, 166.55, 166.27, 156.87, 152.51, 151.79, 141.86, 138.55, 136.63, 129.81, 129.07, 129.03, 127.79, 122.27, 118.76, 107.16, 61.04, 60.42, 52.49, 48.84, 30.67, 26.20, 14.55; GC-MS m/z calcd for $[M]^+$ $C_{29}H_{30}N_6O_7$: 574.59, found: 574.55; Anal. Calcd.: C, 60.62; H, 5.26; N, 14.63; Found: C, 60.89; H, 5.43; N, 14.74.

7.3 *Diethyl (4-(2-(6-methyl-4-oxo-1-phenyl-1,4-dihydro-5H-pyrazolo[3,4-d]pyrimidin-5-yl)acetamido)benzoyl)-L-glutamate (**6b**)*

Off-white powder; 64% yield; aqueous ethanol 50%; Melting point: 280-282 °C; ^{1}H NMR (400 MHz, DMSO-*d*$_6$) δ 10.78 (s, 1H, CH_2CONH), 8.67 (d, *J* = 7.4 Hz, 1H, CON*H*CH), 8.36 (s, 1H, C3-H), 8.10 (d, *J* = 8.0 Hz, 2H, H-Ar), 7.90 (d, *J* = 8.4 Hz, 2H, H-Ar), 7.71 (d, *J* = 8.4 Hz, 2H, H-Ar), 7.60 (t, *J* = 7.8 Hz, 2H, H-Ar), 7.43 (t, *J* = 7.4 Hz, 1H, H-Ar), 5.05 (s, 2H, CH_2CONH), 4.44 (ddd, *J* = 9.8, 7.3, 5.2 Hz, 1H, Cα-H), 4.16 – 3.98 (m, 4H, 2 x OCH_2CH_3),

2.64 (s, 3H, C6-CH_3), 2.46 (t, *J* = 7.5 Hz, 2H, Cγ-H), 2.17 – 2.07 (m, 1H, Cβ-Ha), 2.07 – 1.95 (m, 1H, Cβ-Hb), 1.19 (m, 6H, 2 x OCH_2CH_3); ^{13}C NMR (101 MHz, DMSO-d_6) δ 172.71, 172.34, 166.59, 166.30, 160.37, 157.71, 150.96, 141.86, 138.68, 136.58, 129.76, 129.02, 127.57, 122.13, 118.85, 105.23, 61.06, 60.43, 56.53, 52.50, 47.23, 30.66, 26.19, 24.20, 18.99, 14.53; GC-MS m/z calcd for $[M]^+$ $C_{30}H_{32}N_6O_7$: 588.62, found: 588.79; Anal. Calcd.: C, 61.22; H, 5.48; N, 14.28; Found: C, 61.43; H, 5.66; N, 14.41.

*7.4 Diethyl (4-(2-(3-methyl-4-oxo-1-phenyl-1,4-dihydro-5H-pyrazolo[3,4-d]pyrimidin-5-yl)acetamido)benzoyl)-L-glutamate (**6c**)*

Off-white powder; 68% yield; aqueous ethanol 50%; Melting point: 168-170 °C; ^{1}H NMR (400 MHz, DMSO-d_6) δ 10.75 (s, 1H, CH_2CONH), 8.65 (d, *J* = 7.5 Hz, 1H, CON*H*CH), 8.47 (s, 1H, C6-H), 8.06 (d, *J* = 8.0 Hz, 2H, H-Ar), 7.89 (d, *J* = 8.8 Hz, 2H, H-Ar), 7.70 (d, *J* = 8.9 Hz, 2H, H-Ar), 7.58 (t, *J* = 7.8 Hz, 2H, H-Ar), 7.40 (t, *J* = 7.5 Hz, 1H, H-Ar), 4.93 (s, 2H, CH_2CONH), 4.44 (ddd, *J* = 9.4, 7.3, 5.1 Hz, 1H, Cα-H), 4.15 – 4.04 (m, 4H, 2 x OCH_2CH_3), 2.52 (s, 3H, C3-CH_3), 2.46 (t, *J* = 7.5 Hz, 2H, Cγ-H), 2.11 (p, *J* = 7.3, 6.6 Hz, 1H, Cβ-Ha), 2.07 – 1.96 (m, 1H, Cβ-Hb), 1.20 (t, *J* = 5.1 Hz, 3H, OCH_2CH_3), 1.17 (t, *J* = 5.1 Hz, 3H, OCH_2CH_3); ^{13}C NMR (101 MHz, DMSO-d_6) δ 172.70, 172.35, 166.55, 166.36, 157.52, 152.60, 152.17, 146.52, 141.89, 138.56, 129.70, 129.07, 128.99, 127.28, 121.86, 118.72, 105.28, 61.04, 60.42, 52.49, 48.68, 30.66, 26.19, 14.54, 13.78; Anal. Calcd. for $C_{30}H_{32}N_6O_7$ (588.6210): C, 61.22; H, 5.48; N, 14.28; Found: C, 61.49; H, 5.62; N, 14.51.

*7.5 Diethyl (4-(2-(3,6-dimethyl-4-oxo-1-phenyl-1,4-dihydro-5H-pyrazolo[3,4-d]pyrimidin-5-yl)acetamido)benzoyl)-L-glutamate (**6d**)*

Off-white powder; 71% yield; isopropanol; Melting point: 217-219 °C; ^{1}H NMR (400 MHz, DMSO-d_6) δ 10.76 (s, 1H, CH_2CONH), 8.66 (d, *J* = 7.4 Hz, 1H, CON*H*CH), 8.08 (d, *J* = 8.0 Hz, 2H, H-Ar), 7.93 – 7.84 (m, 2H, H-Ar), 7.74 – 7.66 (m, 2H, H-Ar), 7.57 (t, *J* = 8.0 Hz, 2H, H-Ar), 7.39 (t, *J* = 7.4 Hz, 1H, H-Ar), 5.01 (s, 2H, CH_2CONH), 4.44 (ddd, *J* = 9.9, 7.3, 5.2 Hz, 1H, Cα-H), 4.08 (dq, *J* = 24.0, 7.1, 6.4 Hz, 4H, 2 x OCH_2CH_3), 2.61 (s, 3H, C6-CH_3), 2.53 (s, 3H, C3-CH_3), 2.46 (t, *J* = 7.5 Hz, 2H, Cγ-H), 2.13 (t, *J* = 6.7 Hz, 1H, Cβ-Ha), 2.02 (ddd, *J* = 14.1, 8.3, 4.4 Hz, 1H, Cβ-Hb), 1.19 (m, 6H, 2 x OCH_2CH_3); ^{13}C NMR (101 MHz, DMSO-d_6) δ 172.69, 172.36, 166.55, 166.38, 160.40, 158.30, 151.37, 146.44, 141.91, 138.74, 129.65, 129.04, 128.97, 127.03, 121.68, 118.79, 103.37, 61.04, 60.42, 52.50, 47.02, 30.67, 26.20, 24.25, 14.54, 13.70; Anal. Calcd. for $C_{31}H_{34}N_6O_7$ (602.6480): C, 61.78; H, 5.69; N, 13.95; Found: C, 61.67; H, 5.85; N, 14.13.

*7.6 Diethyl (4-(2-(1-(4-fluorophenyl)-3-methyl-4-oxo-1,4-dihydro-5H-pyrazolo[3,4-d]pyrimidin-5-yl)acetamido)benzoyl)-L-glutamate (**6e**)*

Off-white powder; 75% yield; aqueous ethanol 50%; Melting point: 167-169 °C; ^{1}H NMR (400 MHz, DMSO-d_6) δ 11.03 (s, 1H, CH_2CONH), 8.69 (s, 1H, CON*H*CH), 8.50 (s, 1H, C6-H), 8.15 – 8.00 (m, 2H, H-Ar), 7.92 - 7.82 (m, 2H, H-Ar), 7.80 – 7.67 (m, 2H, H-Ar), 7.49 – 7.30 (m, 2H, H-Ar), 4.97 (s, 2H, CH_2CONH), 4.52 – 4.39 (m, 1H, Cα-H), 4.21 – 3.88 (m, 4H, 2 x OCH_2CH_3), 2.53 (s, 3H, C3-CH_3), 2.32 – 1.82 (m, 4H, Cβ-H, Cγ-H), 1.18 (s, 6H, 2 x OCH_2CH_3); ^{13}C NMR (101 MHz, DMSO-d_6) δ 172.70, 172.35, 166.57, 166.36, 162.09, 159.66, 157.48, 152.77, 152.10, 146.56, 141.98, 134.98, 129.03, 123.90, 118.71, 116.65, 116.42, 105.18, 61.03, 60.41, 52.50, 48.71, 30.68, 26.18, 14.55, 13.75; DEPT135 ^{13}C NMR (101 MHz, DMSO-d_6) δ 152.74, 129.00, 123.94, 123.85, 118.74, 116.59, 116.37, 61.01 (inverted), 60.40 (inverted), 52.51, 48.71 (inverted), 30.70 (inverted), 26.22 (inverted), 14.53,

13.71; Anal. Calcd. for $C_{30}H_{31}FN_6O_7$ (606.6114): C, 59.40; H, 5.15; N, 13.85; Found: C, 59.61; H, 5.34; N, 13.97.

*7.7 Diethyl (4-(2-(1-(4-fluorophenyl)-3,6-dimethyl-4-oxo-1,4-dihydro-5H-pyrazolo[3,4-d]pyrimidin-5-yl)acetamido)benzoyl)-L-glutamate (**6f**)*

Off-white powder; 66% yield; aqueous ethanol 50%; Melting point: 219-221 ºC; ^{1}H NMR (400 MHz, DMSO-*d*$_6$) δ 11.20 (s, 1H, CH_2CONH), 8.73 (ddd, *J* = 14.0, 7.4, 2.2 Hz, 1H, CON*H*CH), 8.11 – 8.07 (m, 2H, H-Ar), 7.92 – 7.88 (m, 2H, H-Ar), 7.75 (d, *J* = 8.7 Hz, 2H, H-Ar), 7.40 (t, *J* = 8.8 Hz, 2H, H-Ar), 5.06 (s, 2H, C*H*$_2$CONH), 4.50 – 4.39 (m, 1H, Cα-H), 4.17 – 3.97 (m, 4H, 2 x OC*H*$_2$CH$_3$), 2.59 (s, 6H, C3-CH$_3$ and C6-CH$_3$), 2.45 (td, *J* = 7.7, 2.1 Hz, 2H, Cγ-H), 2.09 (s, 1H, Cβ-Ha), 2.07 – 1.97 (m, 1H, Cβ-Hb), 1.18 (dt, *J* = 10.6, 7.1 Hz, 6H, 2 x OCH$_2$C*H*$_3$); ^{13}C NMR (101 MHz, DMSO-*d*$_6$) δ 173.20, 172.71, 172.36, 166.56, 166.40, 160.58, 158.24, 151.27, 146.49, 135.14, 128.99, 123.81, 123.72, 118.76, 116.60, 116.37, 103.26, 61.02, 60.41, 52.41, 47.01, 30.68, 26.15, 24.25, 14.55, 13.67; GC-MS m/z calcd for $[M]^+$ $C_{31}H_{33}FN_6O_7$: 620.23, found: 620.09; Anal. Calcd.: C, 59.99; H, 5.36; N, 13.54; Found: C, 60.21; H, 5.60; N, 13.78.

*7.8 Diethyl (4-(2-(1-(4-chlorophenyl)-3-methyl-4-oxo-1,4-dihydro-5H-pyrazolo[3,4-d]pyrimidin-5-yl)acetamido)benzoyl)-L-glutamate (**6g**)*

Off-white powder; 69% yield; abs. ethanol; Melting point: 210-212 ºC; ^{1}H NMR (400 MHz, DMSO-*d*$_6$) δ 10.89 (s, 1H, CH$_2$CON*H*), 8.66 (s, 1H, CON*H*CH), 8.50 (s, 1H, C6-H), 8.23 – 8.02 (m, 2H, H-Ar), 7.99 - 7.81 (m, 2H, H-Ar), 7.81 – 7.41 (m, 4H, H-Ar), 4.94 (s, 2H, C*H*$_2$CONH), 4.51 – 4.31 (s, 1H, Cα-H), 4.25 – 3.79 (m, 4H, 2 x OC*H*$_2$CH$_3$), 2.52 (s, 3H, C3-H), 2.27 – 1.84 (m, 4H, Cβ-H, Cγ-H), 1.30 – 0.99 (s, 6H, 2 x OCH$_2$C*H*$_3$); ^{13}C NMR (101 MHz, DMSO-*d*$_6$) δ 172.70, 172.35, 166.55, 166.31, 157.41, 152.88, 152.31, 146.89, 141.94, 137.46, 131.30, 129.73, 129.05, 128.94, 123.08, 118.71, 105.48, 61.03, 60.42, 52.49, 48.73, 30.67, 26.18, 14.55, 13.78; DEPT135 ^{13}C NMR (101 MHz, DMSO-*d*$_6$) δ 152.84, 129.68, 129.01, 123.05, 118.75, 61.02 (inverted), 60.40 (inverted), 52.51, 48.71 (inverted), 30.70 (inverted), 26.22 (inverted), 14.54, 13.75; GC-MS m/z calcd for $[M]^+$ $C_{30}H_{31}ClN_6O_7$: 622.20, found: 622.27; Anal. Calcd.: C, 57.83; H, 5.02; N, 13.49; Found: C, 58.11; H, 5.17; N, 13.68.

*7.9 Diethyl (4-(2-(1-(4-chlorophenyl)-3,6-dimethyl-4-oxo-1,4-dihydro-5H-pyrazolo[3,4-d]pyrimidin-5-yl)acetamido)benzoyl)-L-glutamate (**6h**)*

Off-white powder; 55% yield; aqueous ethanol 50%; Melting point: 222-224 ºC; ^{1}H NMR (400 MHz, DMSO-*d*$_6$) δ 10.77 (s, 1H, CH$_2$CON*H*), 8.65 (d, *J* = 7.4 Hz, 1H, CON*H*CH), 8.15 (d, *J* = 8.7 Hz, 2H, H-Ar), 7.89 (d, *J* = 8.3 Hz, 2H, H-Ar), 7.70 (d, *J* = 8.4 Hz, 2H, H-Ar), 7.63 (d, *J* = 8.9 Hz, 2H, H-Ar), 5.01 (s, 2H, C*H*$_2$CONH), 4.48 – 4.37 (m, 1H, Cα-H), 4.17 – 4.02 (m, 4H, 2 x OC*H*$_2$CH$_3$), 2.62 (s, 3H, C3-CH$_3$), 2.52 (s, 3H, C3-CH$_3$), 2.45 (t, *J* = 7.6 Hz, 2H, Cγ-H), 2.14 – 2.09 (m, 1H, Cβ-Ha), 2.06 – 1.97 (m, 1H, Cβ-Hb), 1.19 (dt, *J* = 10.1, 7.1 Hz, 6H, 2 x OCH$_2$C*H*$_3$); ^{13}C NMR (101 MHz, DMSO-*d*$_6$) δ 172.71, 172.36, 166.55, 166.33, 160.76, 158.18, 151.51, 146.83, 141.99, 137.62, 131.06, 129.68, 129.00, 128.89, 122.94, 118.77, 103.55, 61.02, 60.42, 52.50, 47.04, 30.67, 26.16, 24.30, 14.55, 13.70; Anal. Calcd. for $C_{31}H_{33}ClN_6O_7$ (637.0900): C, 58.44; H, 5.22; N, 13.19; Found: C, 58.62; H, 5.31; N, 13.45.

*7.10 Diethyl (4-(2-(1-(4-bromophenyl)-3-methyl-4-oxo-1,4-dihydro-5H-pyrazolo[3,4-d]pyrimidin-5-yl)acetamido)benzoyl)-L-glutamate (**6i**)*

White powder; 72% yield; abs. ethanol; Melting point: 214-216 ºC; ^{1}H NMR (400 MHz, DMSO-d_6) δ 10.77 (s, 1H, CH_2CONH), 8.67 (d, *J* = 7.4 Hz, 1H, CON*H*CH), 8.50 (s, 1H, C6-H), 8.07 (d, *J* = 8.9 Hz, 2H, H-Ar), 7.90 (d, *J* = 8.4 Hz, 2H, H-Ar), 7.76 (d, *J* = 8.9 Hz, 2H, H-Ar), 7.70 (d, *J* = 8.5 Hz, 2H, H-Ar), 4.94 (s, 2H, CH_2CONH), 4.44 (ddd, *J* = 9.6, 7.3, 5.3 Hz, 1H, Cα-H), 4.15 – 4.03 (m, 4H, 2 x OCH_2CH_3), 2.54 (s, 3H, C3-CH_3), 2.46 (t, *J* = 7.5 Hz, 2H, Cγ-H), 2.17 – 2.06 (m, 1H, Cβ-Ha), 2.02 (ddd, *J* = 13.6, 9.2, 6.7 Hz, 1H, Cβ-Hb), 1.19 (dt, *J* = 10.8, 7.1 Hz, 6H, 2 x OCH_2CH_3); ^{13}C NMR (101 MHz, DMSO-d_6) δ 172.69, 172.34, 166.55, 166.27, 157.41, 152.84, 152.33, 146.93, 141.86, 137.88, 132.63, 129.06, 129.02, 123.33, 119.61, 118.74, 105.53, 61.04, 60.42, 52.49, 48.70, 30.67, 26.20, 14.55, 13.78; GC-MS m/z calcd for $[M]^+$ $C_{30}H_{31}BrN_6O_7$: 666.14, found: 666.38; Anal. Calcd.: C, 53.98; H, 4.68; N, 12.59; Found: C, 54.17; H, 4.85; N, 12.75.

7.11 *Diethyl (4-(2-(1-(4-bromophenyl)-3,6-dimethyl-4-oxo-1,4-dihydro-5H-pyrazolo[3,4-d]pyrimidin-5-yl)acetamido)benzoyl)-L-glutamate (**6j**)*

Brownish white powder; 56% yield; aqueous ethanol 50%; Melting point: 232-234 ºC; ^{1}H NMR (400 MHz, DMSO-d_6) δ 10.80 (s, 1H, CH_2CONH), 8.67 (d, *J* = 7.4 Hz, 1H, CON*H*CH), 8.09 (d, *J* = 8.8 Hz, 2H, H-Ar), 7.90 (d, *J* = 8.4 Hz, 2H, H-Ar), 7.73 (dd, *J* = 16.9, 8.6 Hz, 4H, H-Ar), 5.01 (s, 2H, CH_2CONH), 4.43 (ddd, *J* = 9.7, 7.3, 5.2 Hz, 1H, Cα-H), 4.10 (ddd, *J* = 24.9, 10.6, 6.3 Hz, 4H, 2 x OCH_2CH_3), 2.62 (s, 3H, C6-CH_3), 2.52 (s, 3H, C3-CH_3), 2.46 (t, *J* = 7.5 Hz, 2H, Cγ-H), 2.15 – 2.08 (m, 1H, Cβ-Ha), 2.05 – 1.96 (m, 1H, Cβ-Hb), 1.19 (dt, *J* = 10.8, 7.1 Hz, 6H, 2 x OCH_2CH_3); ^{13}C NMR (101 MHz, DMSO-d_6) δ 172.69, 172.34, 166.55, 166.29, 160.74, 158.18, 151.53, 146.86, 141.88, 138.04, 132.57, 129.03, 128.99, 123.19, 119.36, 118.81, 103.59, 61.04, 60.42, 52.50, 47.04, 30.67, 26.20, 24.28, 14.54, 13.69; Anal. Calcd. for $C_{31}H_{33}BrN_6O_7$ (681.5440): C, 54.63; H, 4.88; N, 12.33; Found: C, 54.80; H, 5.09; N, 12.49.

7.12 *Diethyl (4-(2-(1-(4-bromophenyl)-3-(methylthio)-4-oxo-1,4-dihydro-5H-pyrazolo[3,4-d]pyrimidin-5-yl)acetamido)benzoyl)-L-glutamate (**6k**)*

White powder; 55% yield; abs. ethanol; Melting point: 176-178 ºC; ^{1}H NMR (400 MHz, DMSO-d_6) δ 10.77 (s, 1H, CH_2CONH), 8.66 (d, *J* = 7.4 Hz, 1H, CON*H*CH), 8.52 (s, 1H, C6-H), 8.08 (d, *J* = 8.8 Hz, 2H, H-Ar), 7.89 (d, *J* = 8.3 Hz, 2H, H-Ar), 7.78 (d, *J* = 7.5 Hz, 2H, H-Ar), 7.69 (d, *J* = 8.4 Hz, 2H, H-Ar), 4.92 (s, 2H, CH_2CONH), 4.43 (dq, *J* = 9.0, 5.6 Hz, 1H, Cα-H), 4.08 (dq, *J* = 24.9, 7.1 Hz, 4H, , 2 x OCH_2CH_3), 2.64 (s, 3H, SCH_3), 2.45 (t, *J* = 7.6 Hz, 2H, Cγ-H), 2.15 - 2.06 (m, 2H, Cβ-Ha), 2.15 - 2.06 (m, 1H, Cβ-Hb), 2.05 – 1.94 (m, 1H, $NHCHCH_2CH_2CO$), 1.18 (dt, *J* = 10.7, 7.2 Hz, 6H, 2 x OCH_2CH_3); ^{13}C NMR (101 MHz, DMSO-d_6) δ 172.69, 172.33, 166.55, 166.15, 156.42, 153.35, 153.09, 146.62, 141.83, 137.76, 132.66, 129.06, 129.03, 123.18, 119.68, 118.76, 105.15, 61.04, 60.42, 52.49, 48.75, 30.66, 26.19, 14.55, 13.23; GC-MS m/z calcd for $[M]^+$ $C_{30}H_{31}BrN_6O_7S$: 698.12, found: 698.34; Anal. Calcd.: C, 51.51; H, 4.47; N, 12.01; Found: C, 51.78; H, 4.60; N, 12.23.

7.13 *Diethyl (4-(2-(1-(4-bromophenyl)-6-methyl-3-(methylthio)-4-oxo-1,4-dihydro-5H-pyrazolo[3,4-d]pyrimidin-5-yl)acetamido)benzoyl)-L-glutamate (**6l**)*

White powder; 77% yield; aqueous ethanol 50%; Melting point: 249-252 ºC; ^{1}H NMR (400 MHz, DMSO-d_6) δ 10.77 (s, 1H, CH_2CONH), 8.66 (d, *J* = 7.4 Hz, 1H, CON*H*CH), 8.10 (d, *J* = 8.5 Hz, 2H, H-Ar), 7.89 (d, *J* = 8.3 Hz, 2H, H-Ar), 7.77 (d, *J* = 8.5 Hz, 2H, H-Ar), 7.69 (d, *J* = 8.4 Hz, 2H, H-Ar), 5.00 (s, 2H, CH_2CONH), 4.53 – 4.34 (m, 1H, Cα-H), 4.14 - 4.0 (m, 4H), 2.63 (s, 3H, SCH_3), 2.62 (s, 3H, C6-CH_3), 2.45 (t, *J* = 7.6 Hz, 2H, Cγ-H), 2.14 – 2.06 (m, 1H, Cβ-Ha), 2.05 – 1.92 (m, 1H, Cβ-Hb), 1.18 (dt, *J* = 10.0, 7.2 Hz, 6H, 2 x OCH_2CH_3); ^{13}C NMR

(101 MHz, DMSO-d_6) δ 172.69, 172.34, 166.53, 166.16, 161.43, 157.21, 152.26, 146.57, 141.83, 137.92, 132.63, 129.03, 123.09, 119.46, 118.83, 103.30, 61.03, 60.42, 52.49, 47.14, 30.67, 26.20, 24.29, 14.55, 13.14; GC-MS *m/z* calcd for $[M]^+$ $C_{31}H_{33}BrN_6O_7S$: 712.13, found: 712.04.; Anal. Calcd.: C, 52.18; H, 4.66; N, 11.78; Found: C, 52.40; H, 4.75; N, 12.04.

*7.14 (4-(2-(3-methyl-4-oxo-1-phenyl-1,4-dihydro-5H-pyrazolo[3,4-d]pyrimidin-5-yl)acetamido)benzoyl)-L-glutamic acid (**7a**)*

White powder; 38% yield; abs. ethanol; Melting point: 257-259 ºC; ^1H NMR (400 MHz, DMSO-d_6) δ 12.42 (s, 2H, 2 x COOH), 10.74 (s, 1H, CH_2CONH), 8.54 (d, *J* = 7.7 Hz, 1H, CON*H*CH), 8.47 (s, 1H, C6-H), 8.06 (d, *J* = 8.1 Hz, 2H, H-Ar), 7.90 (d, *J* = 8.4 Hz, 2H, H-Ar), 7.70 (d, *J* = 8.5 Hz, 2H, H-Ar), 7.58 (t, *J* = 7.8 Hz, 2H, H-Ar), 7.41 (t, *J* = 7.4 Hz, 1H, H-Ar), 4.93 (s, 2H, CH_2CONH), 4.47 – 4.36 (m, 1H, Cα-H), 2.56 (s, 3H, C3-CH_3), 2.38 (t, *J* = 7.5 Hz, 2H, Cγ-H), 2.11 (td, *J* = 10.7, 8.3, 4.6 Hz, 1H, Cβ-Ha), 2.04 – 1.92 (m, 1H, Cβ-Hb); ^{13}C NMR (101 MHz, DMSO-d_6) δ 174.37, 173.99, 166.43, 166.34, 157.53, 152.63, 152.19, 146.54, 141.78, 138.56, 129.73, 129.23, 129.03, 127.32, 121.90, 118.71, 105.28, 52.39, 48.67, 30.89, 26.40, 13.80; GC-MS m/z calcd for $[M]^+$ $C_{26}H_{24}N_6O_7$: 532.51, found: 532.85; Anal. Calcd.: C, 58.64; H, 4.54; N, 15.78; Found: C, 58.82; H, 4.71; N, 16.02.

*7.15 (4-(2-(3,6-dimethyl-4-oxo-1-phenyl-1,4-dihydro-5H-pyrazolo[3,4-d]pyrimidin-5-yl)acetamido)benzoyl)-L-glutamic acid (**7b**)*

Brownish white powder; 42% yield; abs. ethanol; Melting point: 236-238 ºC; ^1H NMR (400 MHz, DMSO-d_6) δ 12.33 (s, 2H, 2 x COOH), 10.69 (s, 1H, CH_2CONH), 8.46 (d, *J* = 7.6 Hz, 1H, CON*H*CH), 7.98 (d, *J* = 8.0 Hz, 2H, H-Ar), 7.81 (d, *J* = 8.4 Hz, 2H, H-Ar), 7.61 (d, *J* = 8.3 Hz, 2H, H-Ar), 7.47 (t, *J* = 7.8 Hz, 2H, H-Ar), 7.28 (t, *J* = 7.4 Hz, 1H, H-Ar), 4.92 (s, 2H, CH_2CONH), 4.32 (ddd, *J* = 10.2, 7.6, 4.8 Hz, 1H, Cα-H), 2.51 (s, 3H, C3-CH_3), 2.43 (s, 3H, C6-CH_3), 2.29 (t, *J* = 7.4 Hz, 2H, Cγ-H), 2.03 (dd, *J* = 10.9, 5.1 Hz, 1H, Cβ-Ha), 1.88 (ddd, *J* = 13.5, 9.6, 6.7 Hz, 1H, Cβ-Hb); ^{13}C NMR (101 MHz, DMSO-d_6) δ 174.37, 174.00, 166.43, 166.37, 160.45, 158.31, 151.38, 146.46, 141.81, 138.73, 129.68, 129.19, 128.99, 127.08, 121.73, 118.77, 103.37, 52.39, 47.03, 30.89, 26.39, 24.27, 13.72; GC-MS m/z calcd for $[M]^+$ $C_{27}H_{26}N_6O_7$: 546.19, found: 546.38; Anal. Calcd.: C, 59.34; H, 4.80; N, 15.38; Found: C, 59.62; H, 4.89; N, 15.57.

*7.16 Diethyl (4-(2-(((R)-5-(ethoxycarbonyl)-4-methyl-6-(p-tolyl)-1,6-dihydropyrimidin-2-yl)thio)acetamido)benzoyl)-L-glutamate (**9a**)*

White powder; 55% yield; aqueous ethanol 60%; Melting point: 98-100 ºC; ^1H NMR (400 MHz, DMSO-d_6) δ 10.40 (s, 1H, CH_2CONH), 9.78 (s, 1H, Py-NH), 8.63 (d, *J* = 7.4 Hz, 1H, CON*H*CH), 7.82 (d, *J* = 8.4 Hz, 2H, H-Ar), 7.49 (d, *J* = 8.3 Hz, 2H, H-Ar), 7.10 (d, *J* = 8.3 Hz, 2H, H-Ar), 6.99 (d, *J* = 7.8 Hz, 2H, H-Ar), 5.49 (s, 1H, Py-C4-H), 4.44 (dt, *J* = 9.0, 4.5 Hz, 1H, Cα-H), 4.10 – 3.99 (m, 8H, 3 x OCH_2CH_3 and SCH_2), 2.45 (d, *J* = 7.5 Hz, 2H, Cγ-H), 2.24 (s, 3H, Py-C6-CH_3), 2.21 (s, 3H, CH_3), 2.13 (q, *J* = 6.0 Hz, 1H, Cβ-Ha), 2.07 – 1.98 (m, 1H, Cβ-Hb), 1.15 (dt, *J* = 14.1, 5.1 Hz, 9H, 3 x OCH_2CH_3); ^{13}C NMR (101 MHz, DMSO-d_6) δ 172.70, 172.35, 167.29, 166.66, 166.58, 150.37, 146.00, 142.87, 142.10, 136.26, 129.48, 129.17, 128.78, 128.64, 127.02, 118.64, 99.31, 61.03, 60.41, 59.56, 59.12, 52.45, 35.32, 30.65, 26.21, 21.09, 17.95, 14.60, 14.53; GC-MS m/z calcd for $[M]^+$ $C_{33}H_{40}N_4O_8S$: 652.76, found: 652.85; Anal. Calcd.: C, 60.72; H, 6.18; N, 8.58; Found: C, 60.51; H, 6.31; N, 8.80.

*7.17 Diethyl (4-(2-(((R)-6-(4-chlorophenyl)-5-(ethoxycarbonyl)-4-methyl-1,6-dihydropyrimidin-2-yl)thio)acetamido)benzoyl)-L-glutamate (**9b**)*

White powder; 60% yield; aqueous ethanol 60%; Melting point: 114-116 ºC; ^{1}H NMR (400 MHz, DMSO-d_6) δ 10.41 (s, 1H, CH_2CONH), 9.87 (s, 1H, Py-NH), 8.63 (d, *J* = 7.3 Hz, 1H, CON*H*CH), 7.84 (d, *J* = 8.4 Hz, 2H, H-Ar), 7.53 (d, *J* = 8.3 Hz, 2H, H-Ar), 7.24 – 7.19 (m, 4H, H-Ar), 5.51 (s, 1H, Py-C4-H), 4.44 (ddd, *J* = 9.7, 7.2, 5.1 Hz, 1H, Cα-H), 4.13 – 3.97 (m, 8H, 3 x OCH_2CH_3 and SCH_2), 2.47 (t, *J* = 7.5 Hz, 2H, Cγ-H), 2.25 (s, 3H, Py-C6-CH_3), 2.16 – 2.08 (m, 1H, Cβ-Ha), 2.07 – 1.96 (m, 1H, Cβ-Hb), 1.21 – 1.12 (m, 9H, 3 x OCH_2CH_3); ^{13}C NMR (101 MHz, DMSO-d_6) δ 172.71, 172.35, 167.14, 166.55, 166.51, 150.76, 146.64, 144.58, 142.12, 131.74, 129.00, 128.92, 128.82, 128.67, 128.59, 128.55, 118.62, 98.63, 61.02, 60.41, 59.68, 58.76, 52.45, 35.37, 30.65, 26.21, 18.00, 14.60, 14.54; Anal. Calcd. for $C_{32}H_{37}ClN_4O_8S$ (673.1780): C, 57.10; H, 5.54; N, 8.32; Found: C, 57.38; H, 5.68; N, 8.54.

*7.18 Diethyl (4-(2-(((R)-6-(4-bromophenyl)-5-(ethoxycarbonyl)-4-methyl-1,6-dihydropyrimidin-2-yl)thio)acetamido)benzoyl)-L-glutamate (**9c**)*

White powder; 58% yield; aqueous ethanol 60%; Melting point: 145-147 ºC; ^{1}H NMR (400 MHz, DMSO-d_6) δ 10.40 (s, 1H, CH_2CONH), 9.87 (s, 1H, Py-NH), 8.63 (d, *J* = 7.5 Hz, 1H, CON*H*CH), 7.84 (d, *J* = 8.8 Hz, 2H, H-Ar), 7.53 (d, *J* = 8.3 Hz, 2H, H-Ar), 7.36 (d, *J* = 8.0 Hz, 2H, H-Ar), 7.15 (d, *J* = 8.3 Hz, 2H, H-Ar), 5.49 (s, 1H, Py-C4-H), 4.50 – 4.39 (m, 1H, Cα-H), 4.12 – 3.97 (m, 8H, 3 x OCH_2CH_3 and SCH_2), 2.50 – 2.40 (m, 2H, Cγ-H), 2.24 (s, 3H, Py-C6-CH_3), 2.16 – 2.10 (m, 1H, Cβ-Ha), 2.06 – 1.99 (m, 1H, Cβ-Hb), 1.22 – 1.13 (m, 9H, 3 x OCH_2CH_3); ^{13}C NMR (101 MHz, DMSO-d_6) δ 172.71, 172.35, 167.15, 166.56, 166.50, 150.76, 146.65, 144.99, 142.12, 131.47, 129.31, 128.83, 128.69, 120.25, 118.62, 98.56, 98.54, 61.03, 60.42, 59.68, 58.84, 52.44, 35.37, 30.66, 26.21, 18.00, 14.59, 14.55; DEPT135 ^{13}C NMR (101 MHz, DMSO-d_6) δ 131.48, 129.32, 128.83, 118.63, 61.02 (inverted), 60.41 (inverted), 59.67 (inverted), 58.86, 52.45, 35.38 (inverted), 30.67 (inverted), 26.23 (inverted), 18.00, 14.59, 14.54; GC-MS m/z calcd for $[M]^+$ $C_{32}H_{37}BrN_4O_8S$: 716.15, found: 716.99; Anal. Calcd.: C, 53.56; H, 5.20; N, 7.81; Found: C, 53.29; H, 5.39; N, 8.05.

8 NCI-60 Screening Methodology

8.1 NCI 60 Cell One-Dose Screen

General Description:

As of early 2007 all compounds submitted to the NCI 60 Cell screen are tested initially at a single high dose (10-5 M) in the full NCI 60 cell panel. Only compounds which satisfy pre-determined threshold inhibition criteria in a minimum number of cell lines will progress to the full 5-dose assay. The threshold inhibition criteria for progression to the 5-dose screen was selected to efficiently capture compounds with anti-proliferative activity based on careful analysis of historical DTP screening data. The threshold criteria may be updated as additional data becomes available.

8.2 NCI 60 Cell Five-Dose Screen

Compounds which exhibit significant growth inhibition in the One-Dose Screen are evaluated against the 60-cell panel at five concentration levels.

The human tumor cell lines of the cancer screening panel are grown in RPMI 1640 medium containing 5% fetal bovine serum and 2 mM L-glutamine. For a typical screening experiment, cells are inoculated into 96 well microtiter plates in 100 µL at plating densities ranging from 5,000 to 40,000 cells/well depending on the doubling time of individual cell lines. After cell

inoculation, the microtiter plates are incubated at 37° C, 5 % CO2, 95 % air and 100 % relative humidity for 24 h prior to addition of experimental drugs.

After 24 h, two plates of each cell line are fixed *in situ* with TCA, to represent a measurement of the cell population for each cell line at the time of drug addition (Tz). Experimental drugs are solubilized in dimethyl sulfoxide at 400-fold the desired final maximum test concentration and stored frozen prior to use. At the time of drug addition, an aliquot of frozen concentrate is thawed and diluted to twice the desired final maximum test concentration with complete medium containing 50 µg/ml gentamicin. Additional four, 10-fold or ½ log serial dilutions are made to provide a total of five drug concentrations plus control. Aliquots of 100 µl of these different drug dilutions are added to the appropriate microtiter wells already containing 100 µl of medium, resulting in the required final drug concentrations.

Following drug addition, the plates are incubated for an additional 48 h at 37°C, 5 % CO2, 95 % air, and 100 % relative humidity. For adherent cells, the assay is terminated by the addition of cold TCA. Cells are fixed *in situ* by the gentle addition of 50 µl of cold 50 % (w/v) TCA (final concentration, 10 % TCA) and incubated for 60 minutes at 4°C. The supernatant is discarded, and the plates are washed five times with tap water and air dried. Sulforhodamine B (SRB) solution (100 µl) at 0.4 % (w/v) in 1 % acetic acid is added to each well, and plates are incubated for 10 minutes at room temperature. After staining, unbound dye is removed by washing five times with 1 % acetic acid and the plates are air dried. Bound stain is subsequently solubilized with 10 mM trizma base, and the absorbance is read on an automated plate reader at a wavelength of 515 nm. For suspension cells, the methodology is the same except that the assay is terminated by fixing settled cells at the bottom of the wells by gently adding 50 µl of 80 % TCA (final concentration, 16 % TCA). Using the seven absorbance measurements [time zero, (Tz), control growth, (C), and test growth in the presence of drug at the five concentration levels (Ti)], the percentage growth is calculated at each of the drug concentrations levels. Percentage growth is calculated as:

[(Ti-Tz)/(C-Tz)] x 100 for concentrations for which Ti>/=Tz

[(Ti-Tz)/Tz] x 100 for concentrations for which Ti<Tz.

Three dose response parameters are calculated for each experimental agent. Growth inhibition of 50 % (GI50) is calculated from [(Ti-Tz)/(C-Tz)] x 100 = 50, which is the drug concentration resulting in a 50% reduction in the net protein increase (as measured by SRB staining) in control cells during the drug incubation. The drug concentration resulting in total growth inhibition (TGI) is calculated from Ti = Tz. The LC50 (concentration of drug resulting in a 50% reduction in the measured protein at the end of the drug treatment as compared to that at the beginning) indicating a net loss of cells following treatment is calculated from [(Ti-Tz)/Tz] x 100 = -50. Values are calculated for each of these three parameters if the level of activity is reached; however, if the effect is not reached or is exceeded, the value for that parameter is expressed as greater or less than the maximum or minimum concentration tested.
